# Micronutrient Requirements and Sharing Capabilities of the Human Gut Microbiome

**DOI:** 10.3389/fmicb.2019.01316

**Published:** 2019-06-12

**Authors:** Dmitry A. Rodionov, Aleksandr A. Arzamasov, Matvei S. Khoroshkin, Stanislav N. Iablokov, Semen A. Leyn, Scott N. Peterson, Pavel S. Novichkov, Andrei L. Osterman

**Affiliations:** ^1^Sanford Burnham Prebys Medical Discovery Institute, La Jolla, CA, United States; ^2^A.A. Kharkevich Institute for Information Transmission Problems, Russian Academy of Sciences, Moscow, Russia; ^3^Department of Physics, P.G. Demidov Yaroslavl State University, Yaroslavl, Russia; ^4^Lawrence Berkeley National Laboratory, Berkeley, CA, United States

**Keywords:** gut microbiome, vitamin metabolism, metagenomics, 16S, comparative genomics

## Abstract

The human gut microbiome harbors a diverse array of metabolic pathways contributing to its development and homeostasis via a complex web of diet-dependent metabolic interactions within the microbial community and host. Genomics-based reconstruction and predictive modeling of these interactions would provide a framework for diagnostics and treatment of dysbiosis-related syndromes via rational selection of therapeutic prebiotics and dietary nutrients. Of particular interest are micronutrients, such as B-group vitamins, precursors of indispensable metabolic cofactors, that are produced *de novo* by some gut bacteria (prototrophs) but must be provided exogenously in the diet for many other bacterial species (auxotrophs) as well as for the mammalian host. Cross-feeding of B vitamins between prototrophic and auxotrophic species is expected to strongly contribute to the homeostasis of microbial communities in the distal gut given the efficient absorption of dietary vitamins in the upper gastrointestinal tract. To confidently estimate the balance of microbiome micronutrient biosynthetic capabilities and requirements using available genomic data, we have performed a subsystems-based reconstruction of biogenesis, salvage and uptake for eight B vitamins (B1, B2, B3, B5, B6, B7, B9, and B12) and queuosine (essential factor in tRNA modification) over a reference set of 2,228 bacterial genomes representing 690 cultured species of the human gastrointestinal microbiota. This allowed us to classify the studied organisms with respect to their pathway variants and infer their prototrophic vs. auxotrophic phenotypes. In addition to canonical vitamin pathways, several conserved partial pathways were identified pointing to alternative routes of syntrophic metabolism and expanding a microbial vitamin “menu” by several pathway intermediates (vitamers) such as thiazole, quinolinate, dethiobiotin, pantoate. A cross-species comparison was applied to assess the extent of conservation of vitamin phenotypes at distinct taxonomic levels (from strains to families). The obtained reference collection combined with 16S rRNA gene-based phylogenetic profiles was used to deduce phenotype profiles of the human gut microbiota across in two large cohorts. This analysis provided the first estimate of B-vitamin requirements, production and sharing capabilities in the human gut microbiome establishing predictive phenotype profiling as a new approach to classification of microbiome samples. Future expansion of our reference genomic collection of metabolic phenotypes will allow further improvement in coverage and accuracy of predictive phenotype profiling of the human microbiome.

## Introduction

The human intestinal microbiota is host to trillions of microbes representing thousands of different species and strains and harboring over ten million genes that are organized into complex metabolic and transcriptional networks (Human Microbiome Project Consortium, 2012; Li et al., 2014). These networks are driving numerous metabolic interactions within the microbial community and with the human host in the context of highly variable dietary supply of nutrients (Yatsunenko et al., 2012; David et al., 2014; Zhang et al., 2014; Wu et al., 2015; Blanton et al., 2016; Hibberd et al., 2017; Sheflin et al., 2017). Mapping and predictive modeling of these networks would significantly impact our understanding of associations between the microbiota composition, human health and a large spectrum of human disease states (Subramanian et al., 2014; Zeller et al., 2014; Kostic et al., 2015; Ejtahed et al., 2016; Imhann et al., 2018). Elucidation of metabolic capabilities and nutrient requirements of gut microbial communities is expected to open new opportunities for diagnostics, prevention and treatment of dysbiosis-related syndromes via rational and personalized selection of probiotics, prebiotics and dietary nutrients. Metabolism of micronutrients that can be produced *de novo* by some but not all gut bacteria, such as B-group vitamins, represents a particularly interesting case as it has the potential to combine metabolic interactions, that may be competitive and cooperative.

Six out of eight B-vitamins analyzed in this study, B1 (thiamin), B2 (riboflavin), B3 (niacin), B5 (pantothenate), B9 (folate) and B6 (pyridoxine), are common biosynthetic precursors of major metabolic cofactors, TPP, FMN/FAD, NAD(P), CoA, THF, and PLP, respectively, essential in all microbes as well as in the mammalian host (McCormick, 2003). Vitamin B7 (biotin) serves as an essential carboxylation/decarboxylation cofactor upon covalent attachment to biotin carboxyl carrier protein (BCCP) playing an important role in lipogenesis, carbohydrate and amino acid metabolism (Marquet, 2010). Vitamin B12 (cobalamin), a precursor of the B12 coenzyme family including cyanocobalamin, methylcobalamin, and adenosylcobalamin (AdoCbl), is essential for all animals and many, but not all bacterial species (Degnan et al., 2014; Danchin and Braham, 2017). B12 is synthesized by bacteria in two phases via: (i) either anaerobic or aerobic upstream coring ring synthesis pathway including pre- or post-synthesis incorporation of a cobalt ion; and (ii) a universal downstream pathway that involves adenylation and attachment of aminopropanol and nucleotide components (Supplementary Figure S1). Another dietary micronutrient included in our analysis, queuine (Q), is a precursor of a sugar nucleotide queuosine, an essential factor of tRNA modification in both prokaryotes and eukaryotes (Fergus et al., 2015). Availability of these micronutrients in the growth media can influence the structure of various microbial communities (Sanudo-Wilhelmy et al., 2014). On the other hand, recent studies of self-sustaining microbial communities in abiotic environments confirmed the importance of syntrophic metabolism and revealed potential mechanisms for community-wide B-vitamins exchange (Romine et al., 2017). Cross-feeding of these micronutrients between producers and non-producers may also play an important role in the human gut microbiome (HGM) given the efficient absorption of dietary vitamins in the upper gastrointestinal tract.

Previously, we combined genomic and functional context analysis in SEED genome integration and analysis platform to identify novel gene families (enzymes, transporters, transcriptional regulators) and pathway variants involved in microbial B-vitamin metabolism (Rodionov et al., 2002a,b, 2003, 2009, 2017; Vitreschak et al., 2002; Osterman et al., 2010; Rodionova et al., 2017). The earlier SEED subsystems-based analysis revealed a comparable representation and mosaic distribution of B vitamin producers and non-producers within a smaller set of 256 HGM genomes (Magnusdottir et al., 2015), supporting the micronutrient sharing hypothesis.

In the current study, we have focused on a detailed reconstruction of all aspects of biogenesis of nine micronutrients (eight B-vitamins and queuosine) including inference of salvage pathways for non-canonical vitamers and specificity for transport systems on a greatly expanded set of reference genomes. This allowed us to establish genomic signatures for numerous functional pathway variants and to classify all studied species by their simplified *binary phenotypes*, as either having the capability to *de novo* synthesize a given cofactor (*prototrophy*) or being strictly dependent on salvage of its precursor(s) from exogenous sources (*auxotrophy*). Binarization of metabolic phenotypes allows us to develop a computational approach to estimate and compare fractional representation of auxotrophs/prototrophs in publicly available HGM samples. The proposed novel metric, *Community Phenotype Index* (CPI), provides a probabilistic estimate of fractional representation of organisms (on a scale 0 – 100%) with a particular vitamin production capability in a metagenomic sample.

A predictive phylotype-to-phenotype profiling approach established in this study can be extended to a broader range of metabolic phenotypes and applied for comparative analysis of general metabolic capabilities vs. nutrient requirements of gut microbial communities as a function of various factors defining their healthy vs. pathological development (Gehrig et al., 2019). The observed correlations and dependencies are expected to provide guidelines for rational development of therapeutic foods and nutrient supplementation.

## Materials and Methods

### Reference Set of HGM Genomes

To build a comprehensive reference collection of genomes representing diverse bacterial HGM species we utilized the following approach. First, we obtained a list of 194 public HGM genomes collected by the MetaHIT consortium in 2010 (Qin et al., 2010). This list included 151 bacterial genomes sequenced by the Human Microbiome Project (HMP), 17 genomes sequenced by MetaHIT and 26 genomes collected from Genbank. The list was combined with 450 HGM genomes from the Human Microbiome Reference Genome Database in 2012^1^ (Human Microbiome Project Consortium, 2012). Finally, we analyzed the collection of ∼1,000 cultured HGM species published in Rajilic-Stojanovic and de Vos (2014). By mapping the HGM species onto the PATRIC genomic database^2^ (Wattam et al., 2017) we selected 2,228 bacterial strains represented by either complete or high-quality draft genomes (Supplementary Table S1). Phylogenetic trees of analyzed species were generated using concatenated alignments of 11 ribosomal proteins (L5, L6, L9, L10, L15, L20, S2, S4, S5, S6, S8). Ribosomal protein sequences were aligned using MUSCLE (Edgar, 2004). The maximum likelihood phylogenetic tree was constructed using RAxML version 8 (Stamatakis, 2015) and visualized via iTOL (Letunic and Bork, 2016).

### *In silico* Metabolic Reconstruction and Phenotype Prediction

For genomic reconstruction and prediction of metabolic phenotypes for eight B-vitamins and queuosine across the entire set of 2,228 reference genomes, we extended a subsystems-based approach implemented in the SEED platform^3^ (Overbeek et al., 2005, 2014) (see Supplementary Figure S2A for workflow overview). For each of the nine studied vitamins/cofactors, all known and inferred components (enzymes, transporters and transcriptional regulators) of biosynthetic and salvage pathways (Supplementary Figure S1) were captured in a respective mcSEED (microbial community SEED) subsystem propagated to all selected HGM genomes (Supplementary Table S2). *In silico* metabolic reconstructions in mcSEED subsystems were based on functional gene annotation using homology-based methods and three genome context techniques: (i) clustering of genes on the chromosome (operons), (ii) co-regulation of genes by a common regulator or a riboswitch, and (iii) co-occurrence of genes in a set of related genomes. Transcriptional regulons for transcription factors (TFs) (BirA, BioQ, NrtR, NiaR, PdxR) and riboswicthes (TPP, FMN, THF, B12) as captured in the RegPrecise database (Novichkov et al., 2013) were used to disambiguate paralogs with related but distinct functions (most importantly, transporters).

Many vitamin biosynthesis pathways contain alternative biochemical modules (routes) implemented by different subsets of enzymes, as well as diverse vitamin transporters. For all identified pathway variants, we established *genomic signatures* represented by a subset of functional roles (signature genes) that are required for *de novo* vitamin/cofactor biosynthesis and/or vitamin salvage (Table 1). These were further translated to *phenotype rules* underlying the assignment of a prototrophic or auxotrophic phenotype for each respective vitamin/cofactor. For the purposes of further quantitative analysis, all identified pathway variants (Supplementary Table S2) are translated to simplified numeric *binary phenotypes* values corresponding to prototrophy (“1”) or auxotrophy (“0”). These values, combined together for all 2,228 reference genomes, comprise a *binary phenotype matrix* (BPM), which was used to assess microbiome-wide biosynthetic capabilities and requirements for nine analyzed micronutrients (Supplementary Table S1).

**Table 1:** *In silico* reconstruction and phenotype prediction for vitamin/cofactor metabolism in a collection of 2,228 HGM reference genomes.

Vitamin [Vitamers]^1^	Cofac-tor^2^	Pathways signature ^3^	Bas. Path. Var.^4^	BP^5^	No. Gen.^6^	Growth requirements ^7^	Transporters for vitamins or vitamers
B1: Thiamine [HET, HMP]	TPP	(ThiF), (ThiS), ThiH/ThiO, ThiG, ThiC, ThiD, ThiE, ThiL/ThiN	P1	1	975	–	B1: ThiT/ThiBPQ/PnuT/ThiV/ThiXYZ2/YkoEDC2 HMP: CytX/ThiXYZ/YkoEDC HET: ThiW/ThiU
		(ThiF), (ThiS), ThiG, ThiC, ThiD, ThiE, ThiL/ThiN	P1^∗^	1	89	(Ig source?)	
		Thi4, ThiC, ThiD, ThiE, ThiL/ThiN	P2	1	45	–	
		(ThiF), (ThiS), ThiH/ThiO, ThiG, ThiD, ThiE, ThiL/ThiN	Ah	0	199	B1; HMP	
		ThiC, ThiD, ThiE, ThiM, ThiL/ThiN	Az	0	114	B1; HET	
		ThiD, ThiE, ThiM, ThiL/ThiN	Ahz	0	452	B1; (HMP+HET)	
		ThiL/ThiN	A	0	354	B1	
B2: Riboflavin	FMN, FAD	(RibA), RibB, RibD, RibH, RibE, RibF	P	1	1644	–	B2: RibU/PnuX/ImpX/RibN/RfnT/RibZ/RibXY/RfnT
		RibF	A	0	584	B2	
B3: Niacin (Nicotinate or Nicotinamide) [Qn, Nr]	NAD, NADP	NadA, NadB/NadB2, NadC, NadD/NadM, NadE	P1	1	1170	–	B3: NiaP/NiaX/NiaY Nr: PnuC
		NadA, NadC, NadD/NadM, NadE	P1^∗^	1	34	(missing NadB?)	
		Tdo, (Kfa), Kmo, Kyn, (Had), NadC, NadD/NadM, NadE	P2	1	12	–	
		PncB/NadV, (PncA), NadD/NadM, NadE	A	0	895	B3	
		PncB, (PncA), NadC, NadD/NadM, NadE	Aq	0	86	B3; Qn	
		NadR	Ar	0	31	Nr	
B5: Pantothenate [Pne, Pnt]	CoA	PanD/PanP, PanB, (PanE/PanG), PanC, CoaA/CoaX/CoaW, CoaB, CoaC, CoaD, CoaE	P	1	1168	–	B5: PanT/PanF Pnt: PanS
		PanB, (PanE/PanG), PanC, CoaA/CoaX/CoaW, CoaB, CoaC, CoaD, CoaE	P^∗^	1	95	(β-ala source?)	
		PanD/PanP, PanC, CoaA/CoaX/CoaW, CoaB, CoaC, CoaD, CoaE	Apt	0	39	B5; Pnt	
		PanC, CoaA/CoaX/CoaW, CoaB, CoaC, CoaD, CoaE	Apt^∗^	0	17	B5; Pnt (b-ala source?)	
		CoaA/CoaX/CoaW, CoaB, CoaC, CoaD, CoaE	A	0	793	B5	
		CoaA/CoaX/CoaW, CoaD, CoaE	Apn	0	91	Pantetheine	
		–	A^∗^	0	25	CoA uptake	
B6: Pyridoxine	PLP, PMP	PdxS, (PdxT)	P1	1	862	–	B6: PdxU/PdxU2
		PdxJ, (PdxA), (PdxH/PdxO)	P2	1	711	–	
		PdxK/PdxK2	A	0	541	B6	
		–	A^∗^	0	114	B6 (missing PdxK?)	
B7: Biotin [Dtb, KAPA, DAPA]	Biotin-(BCCP)	BioF, BioA, BioB, BioD, BioC, (BioG/BioH/BioZ/BioV), BirA	P1	1	797	–	B7: BioY/YigM
		BioF, BioA, BioB, BioD, BioW, BirA	P2	1	246	–	
		BioF, BioA, BioB, BioD, BirA	P^∗^	1	74	(pimeloyl source?)	
		BioA, BioB, BioD, BirA	A3	0	68	B7; Dtb; DAPA; KAPA	
		BioD, BioB, BirA	A2	0	38	B7; Dtb; DAPA	
		BioB, BirA	A1	0	169	B7; Dtb	
		BirA	A	0	836	B7	
B9: Folate	THF-(Glu)_n_	FolE1/FolE2, (FolQ/FolQ1/FolQ2), (FolB/FolB2), FolK, FolP, FolC, PabC, (PabAB), FolA/FolA2/FolM	P	1	1471	–	B9: FolT
		FolE1/FolE2, (FolQ/FolQ1/FolQ2), (FolB/FolB2), FolK, FolP, FolC, FolA/FolA2/FolM	P^∗^	1	415	(pABA source?)	
		FolA/FolA2/FolM	A	0	342	B9	
B12: Cobalamin [Cbi, Cbr, Ba]	Ado-B12	CbiK/CbiX/CbiX2, CbiL, CbiH, CbiF, CbiG, CbiD, CbiJ, CbiT, CbiE, CbiC, CbiA, Co transporter, CbiP, CbiB, CobU, CobS, CobC/CblZ, CobT, CobD, BtuR/PduO	P1	1	628	–	B12: CbrUVT/BtuBCDF
		ChlID, CobN, CbiL, CobG, CbiH, CbiF, CobF, CbiJ, CbiT, CbiE, CbiC, CbiA, Co transporter, CbiP, CbiB, CobU, CobS, CobC/CblZ, CobT, CobD, BtuR/PduO	P2	1	97	–	
		CbiA, CbiP, CbiB, CobU, CobS, CobC/CblZ, CobT, CobD, BtuR/PduO	Aba	0	32	B12; Cbi; Cbr; Ba	
		CbiP, CbiB, CobU, CobS, CobC/CblZ, CobT, CobD, BtuR/PduO	Acbr	0	43	B12; Cbi; Cbr	
		CobU, CobS, CobC/CblZ, CobT, CobD, BtuR/PduO	Acbi	0	193	B12; Cbi	
		BtuR/PduO	A	0	1235	B12	
Q: Queuosine [preQ1, preQ0, CDG]	Q-(tRNA)	QueA, QueG/QueH, QueC, QueF, GCYHI1/GCYHI2, (QueD), QueE, qTGT	P	1	1109	–	Q: QueT/QrtTUVW/YhhQ
		QueA, QueG/QueH, QueC, QueF, qTGT	Ac	0	62	Q; preQ1; preQ0; CDG	
		QueA, QueG/QueH, QueF, qTGT	Ao	0	42	Q; preQ1; preQ0	
		QueA, QueG/QueH, qTGT	Ap	0	601	Q; preQ1	
		qTGT	Aq	0	297	Q	
		–	A	0	117	Not used	


*^*1*^Abbreviations for vitamers and precursors: HMP, 4-Amino-5-phydroxymethyl-2-methylpyrimidine; HET, 5-(2-Hydroxyethyl)-4-methyltiazole; Ig, iminoglycine; Qn, quinolinate; Nr, *N*-ribosyl-nicotinamide; Pne, pantetheine; Pnt, pantoate; β-ala, β-alanine; Dtb, dethiobiotin; KAPA, 7-Keto-8-aminopelargonic acid; DAPA, 7,8-Diaminopelargonic acid; pABA, p-aminobenzoate; Cbi, cobinamide; Cbr, cobyrinate diamide; Ba, cobyrinate; Q, queuine; preQ1, 7-aminomethyl-7-deazaguanine; preQ0, 7-cyano-7-deazaguanine; CDG, 7-carboxy-7-deazaguanine. ^*2*^Abbreviations for cofactors: TPP, thiamine pyrophosphate; FMN, flavin mononucleotide; FAD, flavin adenylyl dinucleotide; NAD, nicotinamide adenylyl dinucleotide; NADP, nicotinamide adenylyl dinucleotide phosphate; CoA, Coenzyme A; (BCCP), biotin carboxyl carrier protein; THF-(Glu)n, (polyglutamyl) tetrahydrofolate; Ado-B12, adenosyl cobalamine; Q-(tRNA), modified tRNA. ^*3*^Special characters used in pathway signatures: Parentheses denote functional roles that are NOT required to be present corresponding to enzymes that were not detected in all prototrophs. ’/’ denotes alternative enzymes with the same functional role (at least one of them is required to be present). Enzymes in red denote universal enzymes that are present in both auxotrophs and prototrophs. ^*4*^Basic Pathway Variants. Asterisk denotes incomplete pathways with one or two essential enzymes missing. ^*5*^Binary Phenotypes. ^*6*^Number of genomes possessing a basic pathway variant. ^*7*^’–’ denotes no growth requirement in predicted prototrophs; comments in parentheses describe missing biosynthetic reactions/enzymes.*

### Microbiome-Wide Phenotype Profiling From 16S rRNA Gene Data

The overall workflow for predictive phenotype profiling is provided in Supplementary Figure S2B. The 16S rRNA gene sequences of the V3–V5 region for 313 stool samples were obtained from HMP website^4^ (Human Microbiome Project Consortium, 2012). We also analyzed a large dataset of stool samples obtained by the American Gut Project (AGP) (McDonald et al., 2018). The V4 region sequencing results for 12,828 AGP samples were obtained from European Nucleotide Archive at EBI (project PRJEB11419). First, we filtered both datasets by read number and length distribution. For HMP, we included samples containing more than 5,000 reads with minimal length 250 nt. For AGP dataset we filtered out samples containing short reads (<150 nt) and less than 10,000 reads per sample. For AGP samples containing a significant fraction of microbial blooms, which are common due to room-temperature storage of samples additional filtering was applied (Amir et al., 2017). As a result, we retained 2,863 AGP and 245 HMP samples for further analysis.

The amplicon sequencing data from HMP and AGP projects were analyzed using the QIIME version 2 (Caporaso et al., 2010). Raw demultiplexed reads were quality filtered, denoised and clustered into Operational Taxonomic Units (OTUs) with representative sequences and calculated read counts (abundances) using the DADA2 plugin with default parameters (p-trunc-len = 250; max-ee = 2.0). As a result, 4,335 and 42,595 OTUs were generated for HMP and AGP datasets, respectively. After filtering OTUs with low abundance, the resulting sets contained 1,298 OTUs for HMP and 3,360 OTUs for the AGP dataset, at that average percentage of removed reads per sample was ∼1.5%. Taxonomic classification of the obtained representative sequences was performed using the NCBI BLAST ToolKit with two reference 16S rRNA gene databases: (i) RDP (Cole et al., 2014, database release 11.5 with taxonomies updated to be consistent with the NCBI Taxonomy database), and (ii) NCBI (version of December 2018 containing 20,792 sequences). A heuristic approach was used to select a species-level consensus taxonomy from a subset of the top blast hits (ordered by identity percent) derived from both reference databases. If species-level consensus assignment was not obtained, then we used the QIIME2-provided feature-classifier plugin with three alternative classifiers (consensus-blast, consensus-vsearch and naive Bayes). Outputs of all three methods were compared and taxonomic assignments with the highest confidence level (equal or exceeding 0.8) were selected. In the case of low confidence levels obtained at the species level or other ambiguities, we repeated the whole procedure to assign consensus taxonomy at the genus level and further at higher taxonomic ranks.

Read counts for each OTU taxonomically assigned by the QIIME2 pipeline were weighted (renormalized) by 16S rRNA gene copy number to account for its variability between different species and to minimize the strongest biases in estimated values of their relative abundances. For this purpose we used the pan-taxa statistics for the rRNA gene copy number provided by the rrnDB database (Stoddard et al., 2015). For each OTU, this number was estimated as a simple mean of respective values provided by rrnDB for the best matching taxonomic group, at species, genus or family level of taxonomic assignment. The obtained renormalized OTU table, along with calculated BPM values, was used as input for predictive phenotype profiling as outlined below.

To assess vitamin production capabilities and requirements for selected HMP and AGP samples, we utilized a beta-version of Phenobiome Profiler tool (PhenoBiome Inc., Walnut Creek, CA, United States^5^). This tool uses BPM and OTU tables to approximate microbiome-wide metabolic phenotypes while minimizing limitations arising from: (i) imprecise and incomplete correspondence of OTUs to reference genomes; (ii) intrinsic heterogeneity of phenotypes within mapped taxonomic groups. To reach a reasonable compromise between coverage and precision, we use a *hierarchical averaging approach*. Briefly, in the first step, OTU taxonomic assignments are mapped onto the reference genome collection at three taxonomic levels (species, genus, and family). For the purpose of further averaging and probabilistic phenotype assignment, every mapped OTU is assigned a mapping weight (*w*) reflecting representation of corresponding genomes in the collection. Thus, for an OTU precisely mapped at the species level, equal weights are assigned to genomes of all strains/isolates of this species that are present in the collection. For OTUs that could not be mapped at the species level, phenotype averaging is performed at the genus level assigning equal weights to all available species within a genus. A similar approach to averaging/weighting is applied to OTUs mapped only at the family level. OTUs that do not map at the family level (typically ≤ 5–7% by abundance) were excluded from phenotype prediction. As a result, a Community Phenotype Matrix (CPM) is computed where an approximate (averaged) phenotype value for every mapped OTU is calculated from respective binary phenotype values (*p*) multiplied by respective weights (*w*) of each genome (*m*). Each value in CPM reflects a probabilistic relative contribution of each OTU (*i*) to the community-wide phenotype:

$$P_{i}=\sum_{m}w_{i,m}p_{m}$$

At the next step, a Community Phenotype Index (CPI, %) for each phenotype in a given sample is then calculated as the total of all respective CPM values (*P*) multiplied by the relative abundances (*A*) of all individual OTUs:

$$\mathrm{CPI}=\sum_{i}A_{i}P_{i}$$

CPI provides a probabilistic estimate of fractional representation (from 0 to 100%) of the analyzed phenotype, in this case a particular micronutrient production capability or prototrophy. Computing CPI for auxotrophy, which is more convenient for assessing micronutrient requirements, is performed the same way but replacing all binary phenotype values (*P*) in BPM by (1-*P*). A prediction error for CPI values (reflecting imprecise mapping and phenotype microheterogeneity) was calculated as:

$$\sigma =\sqrt{\sum_{i}A_{i}^{2}(1-P_{i})P_{i}}$$

The above described phylogeny-based mapping of the obtained taxonomic profiles to the reference collection of genomes with reconstructed BPM yielded on average ∼77% coverage of mapped OTUs by relative abundance at the level of species for either HMP or AGP datasets. At the genus level, the average coverage increased to 96% for HMP and 91% for AGP datasets. Finally, the addition of OTUs mapped only at the family level further increased the overall coverage of mapping to our current reference HGM collection up to 99 and 97% across all samples from HMP and AGP datasets, respectively.

To analyze the distribution of various combinations of vitamin phenotypes in individual taxa we considered possible combinations of individual binary phenotypes as ordered nine-digit strings, termed *phenotypic barcodes.* For instance, the ‘111111101’ barcode corresponds to a prototroph for all vitamins except B12 (multi-prototroph), while ‘010000000’ is an auxotroph for all vitamins except B2 (mono-prototroph). For each sample, we calculated relative contribution of each of 512 possible vitamin barcodes (*P*_obs_), using *binary phenotypes* for individual reference genomes from BPM and previously described OTU mapping weights. The same hierarchical averaging/weighting approach was used for calculation of the relative contribution of vitamin barcodes, where each barcode is treated as an individual feature (composite phenotype). To facilitate further manipulations we switched from barcodes to their cumulative representation, termed *vitamin prototrophy rank* (VPR). For instance, the ‘111111101’ and ‘000000010’ barcodes correspond to ranks 8 and 1, respectively. We further predicted a theoretical distribution for VPRs in each sample assuming that vitamin binary phenotypes in all organisms are independent of each other (the null hypothesis). Stated this way, the null hypothesis follows the Poisson binomial distribution for the expected frequencies (*P*_exp_) of VPRs in a sample, with CPIs acting as independent trials probabilities. To compare the observed vs. expected VPR distributions we computed the ratio of their corresponding probabilities in each sample (*P*_obs_/*P*_exp_) and presented these ratios in logarithmic scale.

### Comparison of Phenotype Predictions With PICRUSt2

The Phylogenetic Investigation of Communities by Reconstruction of Unobserved States (PICRUSt) pipeline allows one to predict the following functional information for 16S metagenomic samples: (i) functional gene content based on KEGG database annotations for reference genomes (Kanehisa et al., 2014); and (ii) metabolic pathway abundances using the pathway rules from MetCyc database (Caspi et al., 2012) and MinPath (Minimal set of Pathways) tool (Ye and Doak, 2009). The latest version of the software (PICRUSt2) was installed in a conda environment under Linux CentOS 7 as specified in the GitHub Wiki manual^6^. To predict MetCyc/MinPath-based abundances of vitamin biosynthetic pathways in the HMP and AGP samples (provided as OTU abundance tables and files with representative sequences), the PICRUSt2 pipeline was used with default parameters. We further used PICRUSt2 to predict phenotype abundances in the same samples using the BPM for vitamin biosynthesis pathways in 2,228 reference genomes. First, we prepared the custom traits table for 2,581 leaves of the PICRUSt2 reference tree that overlapped by NCBI TaxID with the reference HGM genomes analyzed in this work. We then used the PICRUSt2 pipeline with BPM-based custom trait table provided as an input. As the input BPM describes capabilities of reference species to produce vitamins, we did not need to run the MinPath pathway abundance prediction. All pathway and phenotype abundances obtained by PICRUSt2 were normalized by a number of reads in each sample Scattered plots were produced in R using ggplot2 package.

## Results and Discussion

### Genomic Signatures of Vitamin/Cofactor Metabolism

We began this study by selection of the reference collection of bacterial genomes representing the human gastrointestinal microbiota (see Materials and Methods). The selected set included 2,228 genomes representing eleven phyla, 42 orders, 93 families, 226 genera, and 690 species, as well as 173 genomes that have no taxonomically defined species names (Supplementary Table S1). The largest number of reference genomes in this set belong to the Firmicutes (1046 genomes), Proteobacteria (588 genomes), Actinobacteria (311 genomes) and Bacteroidetes (205 genomes) phyla. The Fusobacteria and Tenericutes phyla are represented by 41 and 25 genomes, respectively. The remaining five phyla (Verrucomicrobia, Synergistetes, Spirochaetes, Lentisphaerae, and Planctomycetes) contain from one to five reference genomes.

**FIGURE 1:**
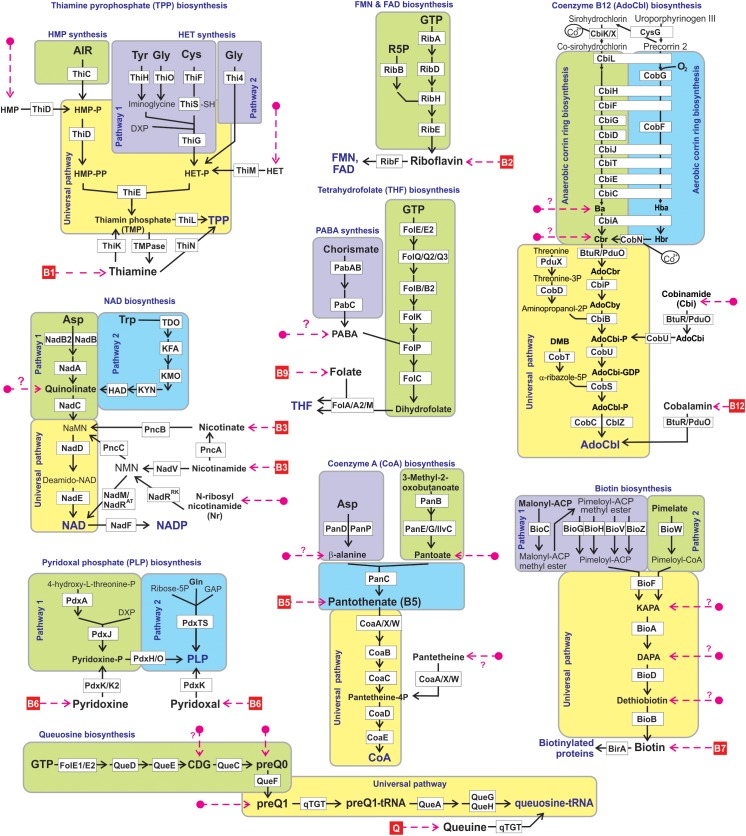
Reconstructed vitamin/cofactor biosynthesis and salvage pathways in HGM genomes. Eight B-vitamins, and queuine (Q) are shown in red boxes. Alternative and universal biosynthetic pathways are marked in blue text and highlighted in colored blocks. Biosynthetic reactions and vitamin/vitamer uptake are depicted by solid black and red dashed lines, respectively. Enzymes are shown by white boxes. The detailed information of enzyme commission (EC) numbers, functional annotations, metabolite abbreviations, and transporter names are provided in Supplementary Figure S1.

We used a subsystems-based comparative genomics approach (Overbeek et al., 2005; Osterman et al., 2010) to reconstruct biosynthetic pathways for nine universally essential cofactors along with biogenesis, salvage and transport systems for their respective metabolic precursors (eight B-group vitamins and queuosine). The overall workflow for metabolic pathway reconstruction and phenotype assignments is provided in Supplementary Figure S2A. The analyzed functional roles include 127 distinct enzymes involved in 9 vitamin biosynthesis/salvage pathways, and 83 transporters (or their components) involved in salvage of exogenous vitamins or their metabolic precursors (Figure 1). Detailed pathway diagrams capturing variations in vitamin and cofactor biosynthetic and salvage pathways at the level of individual enzymes (non-orthologous replacements) and topologies (alternative biochemical routes) are presented in Supplementary Figure S1. From the obtained genomic distribution of vitamin/cofactor biosynthetic enzymes and transporters we deduced *phenotype rules* (generalized genomic signatures, as captured in Table 1) and then assigned individual pathway variants (Ye et al., 2005) to each analyzed genome (Supplementary Table S2). Phenotype rules describe sets of genes whose presence or absence in the analyzed genomes permit confident distinction between metabolic pathway variants and prediction of vitamin transport capabilities. For each vitamin/cofactor, all metabolic pathway variants were subdivided into two major categories: (i) *prototrophic* (variant code “P”) that are capable of *de novo* cofactor synthesis, and (ii) *auxotrophic* (variant code “A”) that are dependent on the uptake and salvage of a respective vitamin or an alternative metabolic precursor from an exogenous source. Distinct P-variants were assigned to prototrophs with alternative *de novo* biosynthetic routes. Thus, variations in B1/TPP biogenesis include two alternative routes for the synthesis of a hydroxyethylthiazole (HET) moiety: a common bacterial variant P1 (ThiFGHS signature) and a eukaryotic-like variant P2 (Thi4 signature). Likewise, two alternative routes to synthesize the quinolinate precursor of NAD comprise the most common bacterial variant P1 (NadAB) and a relatively rare eukaryotic-like variant P2 (Tdo-Kmo-Kyn). Similar variations occur in PLP synthesis (PdxAJ vs. PdxST routes) and in the synthesis of pimeloyl-CoA precursor of biotin (via BioC vs. BioW). B12 is synthesized via either anaerobic or aerobic pathway for corin ring biosynthesis that are characterized by either early or late insertion of cobalt via specific chelatases (CbiK/X vs. CobN) and pathway-specific enzymes (CbiDG vs. CobFG).

Many bacterial genomes that completely lack the *de novo* biosynthetic machinery but typically have vitamin uptake, salvage and downstream biotransformation pathways yielding physiologically active cofactors are generally classified as auxotrophic A-variants. Despite substantial variations in salvage of cofactor precursors (see below), this classification is strengthened by indisputable universal essentiality of these cofactors (with the exception of B12 as discussed below). Downstream pathways common for both P- and A-variants that include from one to five conserved enzymatic steps are characteristic of all analyzed cofactors, except PLP (marked red in Table 1). For the latter, *de novo* and salvage routes are topologically non-overlapping (Figure 1). The only other exception is A^∗^-variant of Coenzyme A biosynthesis (Table 1) in parasitic species of Mollicutes (such as *Mycoplasma/Ureaplasma*) due to their unique capability to uptake a mature cofactor.

Signature salvage enzymes (those that are not involved in *de novo* or downstream pathways) are characteristic of vitamins B1 (ThiK, ThiM, and ThiN), B3 (PncA, PncB and, more rarely, NadV) and B6 (PdxK/K2). Other vitamins and metabolic precursors can feed directly into respective cofactor biosynthetic pathways upon uptake from the media via specific transporter systems. In this study, we have performed a comprehensive mapping of all known and predicted components of salvage enzymes and transporters (see below), which in many cases provided important details on metabolic requirements and capabilities of respective species. However, the presence of particular salvage genes is not required for the general inference of auxotrophy (A-variants). Thus, PdxK/K2 kinase was missing in 116 B6 auxotrophs, suggesting existence of yet unknown alternative forms of this essential salvage enzyme. Such requirement would be particularly unsustainable with respect to uptake transporters that represent a substantial bioinformatic challenge due to their evolutionary plasticity (poorly resolved paralogs) and incomplete knowledge (missing genes).

It is important to note that salvage machinery is often, but not always, present in species with complete P-variants, which allows them to implement an *opportunistic* lifestyle switching from energetically expensive *de novo* synthesis to a more economic salvage depending on the availability of vitamins. Therefore, we have included such capabilities in our overall reconstruction (Supplementary Table S2), despite a caveat of presently incomplete and imprecise knowledge of uptake transporters. It is also recognized that salvage enzymes are often involved in cofactor recycling within the cell, as best studied for the case of NAD(P) cofactors (Figure 1) (Sorci et al., 2010). Moreover, it is tempting to speculate that at least some of the vitamin transporters may contribute to a hypothesized B-vitamin sharing in microbial communities (Romine et al., 2017) (as further discussed below).

Finally, taking into account that the auxotrophy is inferred based mostly on “negative” evidence, the absence of biosynthetic capabilities, we recognize that, at least in principle, the existence of yet unknown and radically different *de novo* cofactor biosynthetic routes in rare bacterial species cannot be excluded. However, our current analysis of vitamin subsystems in thousands of sequenced bacterial genomes strongly argues against this theoretical possibility. Instead, some species harbor “incomplete” variants of known pathways with one or two “missing genes” (see the next section). It is important to emphasize that the variety of incomplete pathway variants and their representation in the entire reference collection are relatively small confirming that our current understanding and genomic reconstruction of bacterial of vitamin/cofactor biosynthetic machinery are nearly complete.

### Incomplete Pathway Variants and Salvage of Alternative Vitamers

Distinguishing between incomplete P-variants with “missing genes” (Osterman and Overbeek, 2003) (variant code P^∗^), which reflect yet unknown non-orthologous gene displacements or alternative biochemical routes, and non-canonical A-variants presents a particular bioinformatic challenge, requiring a detailed case-by-case analysis (Table 1) (note that genome sequencing or gene calling gaps do not affect this analysis as pathway variants are only considered when detected in multiple genomes). Thus, 89 genomes including 62 *Bifidobacterium* strains possess an incomplete B1 pathway with missing iminoglycine synthase (ThiO or ThiH), however, all other B1 biosynthetic enzymes are present (Figure 1). Based on these considerations, these species were tentatively assigned as prototrophs with yet unknown enzyme/route supplying the iminoglycine precursor (P^∗^-variant). In the B3 pathway, NadB (or an alternative enzyme NadB2) catalyzing the synthesis of iminoaspartate from aspartate is missing in 34 predicted B3 prototrophs (P^∗^ including 17 strains of *Helicobacter pylori* and 8 strains of *Micrococcus luteus*, suggesting the presence of a yet unknown alternative iminoaspartate synthesis enzyme in these species. In the B5 pathway, we identified 95 strains with missing aspartate decarboxylase (PanD or PanP) that possess all other *de novo* B5 synthesis enzymes, suggesting the existence of yet uncharacterized alternative enzyme(s) or biochemical route(s) for β-alanine synthesis in these bacteria. Indeed, alternative routes generating β-alanine unrelated to cofactor metabolism (e.g., via alanine racemase or catabolism of pyrimidines) are known. Moreover, β-alanine can be theoretically salvaged, if available, from the growth media. Based on these considerations, we classify the respective pathway variants as P^∗^-type prototrophs rather than auxotrophs salvaging non-canonical vitamers (discussed below).

In the B7 pathway, 74 species from diverse phyla lack the upstream enzymes involved in the synthesis of pimeloyl precursor but possess all downstream biosynthetic enzymes required for assembly of the fused heterocyclic rings of biotin (BioF, BioA, BioD, and BioB). Given the known variability of pathways and isozymes involved in the upstream biotin biosynthesis, we propose the existence of yet uncharacterized enzymes for pimeloyl precursor synthesis in these species. Finally, in the B9 pathway, 454 strains lack the complete pathway for the synthesis of para-aminobenzoate (pABA) precursor, while all other folate biosynthetic enzymes are present in their genomes. There are two potential explanations for these incomplete B9 pathways: (i) presence of yet unknown pABA synthesis routes/enzymes, and/or (ii) salvage of pABA from the diet or other community members. In agreement with the latter hypothesis, it is known that *Lactobacillus* strains having this pathway variant can produce folate only when pABA is added to the medium (Santos et al., 2008; Wegkamp et al., 2010). We chose to classify this variant as P^∗^ despite its similarity with salvage of alternative vitamers in A-variants discussed below, mainly because pABA, a known intermediate in the synthesis of other essential metabolites (e.g., aromatic amino acids), may not be considered a true B9 vitamer. At the same time, a number of other identified incomplete pathway variants in metabolism of B1/TPP, B3/NAD, B5/CoA, B7, B12, and queuosine were classified as *bona fide* auxotrophic A-variants with a potential to salvage alternative vitamers, non-canonical metabolic precursors of respective cofactors (Table 1).

The largest variety of such variants is observed in the biogenesis of *thiamine pyrophosphate (TPP) cofactor*, which is synthesized via coupling of the phosphorylated hydroxymethylpyrimidine (HMP-PP) and thiazole (HET-P) moieties (Figure 1). Nearly half of the analyzed genomes contain complete signatures for the *de novo* biosynthesis of both HMP-PP and HET-P biosynthesis (B1 prototrophs). Auxotrophic variants comprising another half were subdivided into four major sub-variants with distinct biosynthetic/salvage capabilities. The first group of auxotrophs (452 genomes) is unable to synthesize either B1 precursor but possess the ThiM and ThiD kinases, which can convert salvaged HET and HMP precursors into TPP (Ahz-variant in Table 1). Two other groups encode partial TPP biosynthetic pathways: (i) HMP auxotrophs (199 genomes) are capable of synthesizing only HET-P *de novo* and use ThiD kinase to generate HMP-PP from salvaged HMP precursor (Ah-variant in Table 1); (ii) conversely, HET auxotrophs (114 genomes) have only HMP-PP biosynthetic capabilities and generate HET-P via uptake of exogenous HET and its phosphorylation by ThiM kinase (Az-variant in Table 1). Importantly, in all these variants the ability to utilize alternative B1 vitamers or combination thereof is in addition and not instead of a common B1 salvage capability. The remaining group of B1 auxotrophs (354 genomes) can only salvage vitamin B1 but lack the machinery to salvage HET or HMP and combine them into TMP intermediate (A-variant in Table 1).

Biogenesis of *NAD(P) redox cofactors* is also associated with a variety of salvage/recycling pathways from one or both forms of vitamin B3: (i) nicotinic acid (or niacin), the most common form, via PncB salvage enzyme; and (ii) nicotinamide, via one of the three alternative routes, via PncA-PncB or, more rarely in bacteria, via NadV-PncC or NadV-NadM route bypassing otherwise nearly universal downstream enzymes NadD-NadE (Figure 1). Among B3 auxotrophs (1012 strains), we found 86 strains representing diverse taxa such as *Campylobacter, Corynebacterium, Lactobacillus*, and *Streptococcus* that contain a truncated *de novo* NAD biosynthetic pathway variant (Aq-variant in Table 1) comprised of a single enzyme NadC but none of the upstream enzymes involved in quinolinate production. This genomic signature suggests that, in addition to canonical B3 salvage, these species can also salvage quinolinate as previously demonstrated for *Streptococcus pyogenes*, (Sorci et al., 2013). Notably, quinolinate is a common intermediate of NAD biosynthesis not only in bacteria but in some host tissues that synthesize NAD via aerobic degradation of tryptophan (kynurenine pathway). Yet another rare salvage pathway is a two-step conversion of nicotinamide riboside (Nr) to NAD via bifunctional NadR enzyme. This pathway is the only route of NAD biogenesis in *Haemophilus influenza* and related species from the Pasteurellales order that should be considered Nr auxotrophs rather than B3 auxotrophs (Ar-variant in Table 1). However, in a limited number of NadR-containing HGM genomes, it is present in addition to (not instead of) B3 salvage pathways.

A five-step universal downstream pathway (Figure 1) of *Coenzyme A (CoA)* synthesis from pantothenate (vitamin B5) is present (with some non-orthologous variations in the first step) in both, prototrophs (1,263 genomes, 57%) and most, but not all predicted B5 auxotrophs (874 strains, 39%). In addition, 91 strains (mostly *Bifidobacterium* and diverse Clostridiales) lacking *de novo* synthesis have an incomplete universal pathway variant with missing CoaBC bifunctional enzyme catalyzing the conversion of phosphopantothenate to phosphopantetheine. We propose that these bacteria are dependent on an earlier hypothesized “pantetheine shunt,” a salvage of an alternative vitamer pantetheine (CoA degradation product) via a secondary activity of pantothenate kinase converting it directly to phosphopantetheine, thus by-passing both steps requiring a missing CoaBC enzyme (Ye et al., 2005; Osterman, 2009). Indeed, an additional pantetheine kinase activity was experimentally demonstrated for selected pantothenate kinases (Strauss et al., 2010). Notably, this genomic pattern (Apn-variant in Table 1) also cannot be classified as genuine B5 auxotrophy as respective species would not be able to synthesize CoA from pantothenate. Among B5 auxotrophs with incomplete *de novo* pathways are two variants lacking pantoate biosynthesis: (i) Apt-variant in 40 genomes that contain two *de novo* enzymes, PanD and PanC; (ii) Apt^∗^-variant in 16 genomes that retain only one *de novo* enzyme, PanC. Similar to an Aq-variant in NAD biogenesis, this genomic signature suggests the possibility of salvaging pantoate (with known or unknown source of β-alanine, respectively) in addition to vitamin B5.

More than 80% of identified *B7 auxotrophs* (836 genomes) lack all four enzymes responsible for the conversion of pimeloyl-CoA precursor to biotin (Figure 1). The remaining auxotrophs possessing incomplete biosynthetic pathways were classified into three variants (Table 1) characterized by the presence of: (i) only the last enzyme, BioB (A1-variant); (ii) the last two enzymes, BioB and BioD (A2-variant); and (iii) the last three enzymes, BioA, BioD, and BioB (A3-variant). Several considerations suggest that the sustainability of these variants is due to salvage of all three pathway intermediates, namely dethiobiotin, 7-keto-8-aminopelargonic acid (KAPA) and 7,8-diaminopelargonic acid (DAPA) as alternative vitamers, rather than due to the existence of yet unknown alternative enzymes or biosynthetic routes. *First*, the fact that only upstream (but not midstream or downstream) truncations are observed makes this interpretation more consistent with pathway topology. *Second*, a sporadic phylogenetic distribution of these variants among diverse Actinobacteria, Proteobacteria and Firmicutes genomes is more consistent with gene loss rather than non-orthologous replacement.

In contrast to other B-vitamin related cofactors, various *derivatives of B12* are not universally essential in bacteria. While most bacteria have B12-dependent methionine synthase and ribonucleotide reductase, these enzymes are often dispensable due to the presence of alternative B12-independent enzymes and metabolic routes (Rodionov et al., 2003). This points to a distinction between beneficial but not mandatory salvage of B12 by respective A-variants (Table 1) and *life-or-death* requirements of the exogenous supply of metabolic precursors of all other analyzed cofactors. Nevertheless, the effect of B12 availability on fitness of some gut-colonizing *Bacteroides* spp. was reported (Degnan et al., 2014) providing the first experimental evidence of vitamin exchange in gut microbial consortia.

As in the case of biotin, more than 80% of B12 auxotrophs (1235 strains) lack all enzymes of anaerobic or aerobic upstream corin ring synthesis, as well as most downstream enzymes, except BtuR/PduO family adenosyltransferase converting vitamin B12 (cobalamin) to an active coenzyme B12 (adenosylcobalamin). Likewise and following the same reasoning, the three observed incomplete pathway variants (Aba, Acbr, and Acbi, Table 1) appear to point to salvage of alternative B12 vitamers, cobinamide (Cbi), cobyrinate diamide (Cbr) and cobyrinic acid (Ba), respectively (Figure 1).

Our current knowledge of salvage pathways related to *Queuosine*, an essential micronutrient, which, despite many similar metabolic features, is not considered a B-vitamin. A fully prototrophic P-variant, for the *de novo* synthesis of queuosine-tRNA from GTP is present in nearly half of the analyzed genomes (Table 1). A minimal salvage route (Aq-variant) containing only one conserved downstream enzyme, qTGT tranglycosylase, which can generate the same final product directly from the salvaged queuine (Q) precursor, occurs in nearly 300 genomes. However, the most frequent incomplete variant (Aq in 601 genomes, see Table 1) likely entails salvage of preQ1 precursor (see Figure 1). Two other variants of incomplete pathways (Ac and Ao) identified in 62 and 42 genomes, respectively, point to two other pathway intermediates, 7-carboxy-7-deazaguanine (CDG) and 7-cyano-7-deazaguanine (preQ0), as alternative salvageable vitamers. Finally, 117 strains including *Mycoplasma/Ureaplasma* and many *Lactobacillus* spp. lack all queuosine biosynthetic enzymes including qTGT. However, unlike in the case of A^∗^-variant in CoA-related auxotrophs, this entirely “void” genomic signature likely reflects a complete loss of Q-modified tRNAs, at least in parasitic Mollicutes (De Crecy-Lagard et al., 2007).

Overall, the current analysis of incomplete pathways allowed us to reconcile most of the gaps and inconsistencies in the *in silico* reconstruction of vitamin/cofactor biogenesis and salvage. It confirmed a rather comprehensive understanding of these aspects of metabolism across the entire set of reference HGM genomes setting the stage for the further microbiome-wide predictive phenotype profiling (as described in further sections). Of no less importance, it revealed a set of well-defined open problems, such as missing genes and yet unknown biochemical routes, a subject of bioinformatics-driven gene and pathway discovery. Additionally, this analysis tentatively implicated several vitamers as additional or alternative metabolic precursors of respective cofactors as a subject of non-canonical salvage, at least in the species carrying particular partial pathways. These metabolic precursors along with canonical B-vitamins are a likely subject of cross-feeding between donors (at least some prototrophs) and acceptor (auxotrophs) mediated by a variety of specialized transport systems.

### Genomic Distribution of Vitamin Transport Systems

A paramount importance of transporters for vitamin uptake and salvage notwithstanding, especially in auxotrophs, we typically did not include them in gene signatures defining major phenotype rules (for the reasons discussed above). On the other hand, specificity assignment of candidate transporters can be achieved with higher accuracy in the genomic and functional context of auxotrophs (as constrained by their well-defined metabolic requirements) and then conservatively projected to prototrophs. Vitamin/vitamer transport systems that are present in many prototrophs can contribute to their opportunistic (energy-saving) lifestyle and, potentially, to cross-feeding as discussed below. All identified and tentatively assigned transport systems are captured in respective subsystems (Supplementary Table S2) and corresponding pathway diagrams (Supplementary Figure S1). In this brief overview we highlight only some of them focusing on tentatively identified transport systems for alternative vitamers implicated by the analysis of incomplete salvage pathways in the previous section.

Thus, the analyzed distribution of potential uptake transporters for B1 vitamers allowed us to establish the presence of candidate HMP transporters in most HMP auxotrophs (97%), while the HET transporter ThiW was identified in only 64% of HET auxotrophs. In the group of dual HMP/HET auxotrophs, both HET and HMP transporters are present in 259 genomes (57%), and additional ∼100 genomes from this group contain one of the two transporter types. Incomplete knowledge of transporters for thiamine precursors notwithstanding, this analysis suggests that salvage of B1 and its vitamers plays a prominent role in metabolic interaction within gut microbial communities. These transporters are also present in many genomes with P-variants providing the respective species with additional salvaging and, potentially, cross-feeding capabilities.

Incomplete knowledge of transport systems and mechanisms is particularly obvious in otherwise well-studied B3/NAD metabolism. Thus, despite many attempts, no committed B3 transporter has been yet identified even in *Escherichia coli* K12, which is well-known to have a robust nicotinate and nicotinamide salvage capability via PncA-PncB pathway seamlessly compensating for gene deletions in its *de novo* NadB-NadA-NadC pathway (Rowe et al., 1985). Indeed, the presence of B3 transporters from three known families (NiaP, NiaX, and NiaY) were confidently detected only in 25% of all B3 auxotrophs in our reference HGM genome collection (mostly in various Firmicutes but also in some Actinobacteria and Enterobacterales). No specific transporter has been yet identified in the genomes with quinolinate salvage pathway. On the other hand, a committed Nr transporter, PnuC, is consistently present in 31 analyzed genomes from the Pasteurellales order that is solely dependent on the Nr salvage pathway.

Uptake of vitamin B5 in bacteria is mediated by either PanF or PanT transporters, whereas PanS was characterized as a pantoate transporter in *Salmonella* (Ernst and Downs, 2015). Orthologs of known B5 transporters were identified in 75% of all B5 auxotrophs but only in 12% of ∼90 pantetheine auxotrophs pointing to the incomplete knowledge of respective transport systems. Orthologs of PanS transporter were identified in 153 genomes including 35 auxotrophs from the Clostridiales, Tissierellales and Veillonellales orders of Firmicutes. Each of these auxotrophs as well as five prototrophs contain the conserved *panD-panC*-*panS* operon encoding the complete pantoate salvage pathway pointing to the relevance of this B5 vitamer salvage (and, potentially, cross-feeding) in HGM.

The majority of B7 auxotrophs (78%) contain the Energy-Coupling Factor (ECF) family BioY transporters. An even higher fraction of auxotrophs with incomplete biotin salvage pathways (240 out of 275, 87%) also harbor one or two copies of BioY-family transporters. In addition, a secondary transporter YigM enabling an optional B7 salvage in prototrophic *E. coli* and many other enterobacteria was found in 13 genomes of ε-proteobacteria from the Campylobacterales order that are characterized by either A1 or A3 incomplete pathway variants. These observations suggest that both types of biotin transporters may have a wider specificity for at least some B7 vitamers. So, for the closest B7 precursor, dethiobiotin, this conjecture is supported by their uptake competition observed in *E. coli* (Prakash and Eisenberg, 1974). The reported excretion of B7 vitamers by prototrophic strains of enterobacteria (Ohsugi et al., 1990) supports the hypothesis about their potential role in cross-feeding in microbial communities.

Orthologs and paralogs of known B12 uptake transporters, namely BtuFCD(B) of ATP-binding Cassette (ABC) family and CbrT of the ECF family, were found in one or several copies in nearly half of A-variants including 268 genomes with incomplete pathways suggesting their involvement in the uptake of alternative B12 vitamers. Indeed, representatives of both B12 transporter families were experimentally shown to import both B12 and Cbi (Kenley et al., 1978; Butzin et al., 2013; Santos et al., 2018; Wexler et al., 2018). Therefore, identification of transport systems for B12 and its metabolic precursors in numerous HGM genomes remains an open problem.

The ECF-family preQ1 transporters QueT and QrtT have previously been predicted in diverse bacterial species (Rodionov et al., 2009) and recently validated experimentally in *C. difficile* (de Crecy-Lagard, personal communication). Besides, a previously uncharacterized transporter family YhhQ (COG1738) was implicated in the import of queuosine precursors (Zallot et al., 2017; de Crecy-Lagard, personal communication). Representatives of these three families of transporters are widely distributed among the analyzed genomes including 72% of Q auxotrophs and 95% of preQ1/preQ0/CDG auxotrophs (Supplementary Table S2) supporting a potential physiological relevance of HGM-wide salvage and sharing of Q vitamers.

Metabolite cross-feeding in microbial communities, which, according to some reports, extends beyond micronutrients and includes some amino acids (Mee et al., 2014), may be mediated by at least two distinct supply mechanisms: (i) “passive” mechanism, via partial cell lysis; and (ii) “active” mechanism, via specific or/and general efflux transport systems. Although, at first glance, the passive mechanism, (which, for HGM communities, may also include lysis of the host epithelial cells) appears more straightforward, it is tempting to hypothesize the co-existence of a more symbiotic active mechanism of vitamin sharing. Moreover, while all genomes contain numerous relatively non-specific efflux transport systems, which theoretically may contribute to vitamin/vitamers excretion (e.g., in case of their excessive accumulation), the existence and exact nature of such systems is a subject of experimental rather than bioinformatic analysis. Therefore, in our current analysis, we set out to explore another theoretical possibility that active and specific vitamin excretion can be mediated by at least some families of vitamin uptake transporters working “in reverse.” Such hypothesis emphasizing secondary active transporters or active facilitators (e.g., permeases from Major Facilitator Superfamily or MFS family) and channel-type facilitators (Shi, 2013; Saier et al., 2014) as more natural candidates for bi-directional transport as compared to primary active vitamin transporters (such as ABC or ECF family transporters), was previously examined in the context of environmental microbial consortia (Romine et al., 2017).

**FIGURE 2:**
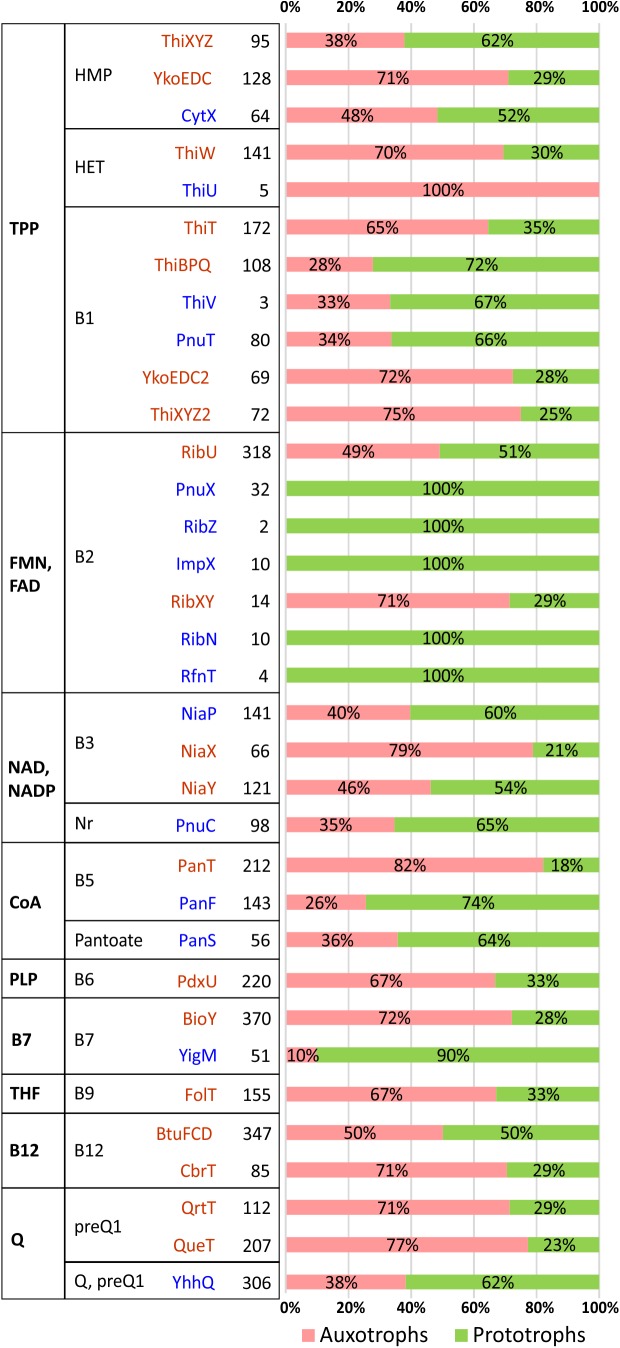
Distribution of vitamin/vitamer transporters in HGM species. Transporters are grouped by a cofactor **(first column)** and vitamin/vitamer **(second column)**. Primary active transporters from the ABC or ECF family are shown in dark red, while secondary active transporters/facilitators (e.g., permeases from MFS family) are in blue. The total number of species possessing a specific transporter is shown in bold with relative contribution of corresponding vitamin auxotrophs/prototrophs shown as a pink/green bar.

It is important to emphasize that while our knowledge of vitamin uptake transporters is incomplete, the knowledge of vitamin efflux is nearly non-existent. Some anecdotal evidence implicating vitamin transporters is available for industrial vitamin producers such as for riboflavin-producing microbial strains (Hemberger et al., 2011; McAnulty and Wood, 2014). For a more systematic bioinformatic analysis, we surveyed distribution and co-occurrence of various families of vitamin transporters and predicted metabolic phenotypes across bacterial species from our collection (Figure 2).

Despite a predominantly mosaic distribution, some potentially interesting trends were also observed. Thus, facilitator-class riboflavin transporters (PnuX, RibZ, ImpX, RibN, RfnT) were observed only in B2 prototrophs, whereas the primary active transporters, RibU (ECF family) and RibXY (ABC superfamily) are present in both, prototrophs and auxotrophs. Among similar, albeit less contrasting trends are: (i) the presence of the biotin permease, YigM in the majority of B7 prototrophs contrasting with BioY (ECF family) preferential occurrence in auxotrophs; (ii) more frequent occurrence of pantothenate facilitator transporter PanF in B5 prototrophs vs. the opposing trend of PanT (ECF family) to occur mostly in B5 auxotrophs; (iii) similar co-occurrence trends for thiamine permease PnuT vs. ThiT (ECF family). The observed trends, with the caveats pertaining to incomplete data, statistical significance and possible alternative interpretations, are consistent with a possible role of at least some secondary transporters in the excretion and community-wide sharing of respective vitamins/vitamers. If experimentally confirmed, these trends may provide presently unknown genomic signatures distinguishing potential vitamin donors from other prototrophs with opportunistic salvaging rather than sharing capabilities.

### Comparison of Predicted B-Vitamin Phenotypes With Published Experimental Data

To assess robustness of the predicted vitamin auxotrophic and prototrophic phenotypes, we searched published experimental data on nutritional requirements and B-vitamin production capabilities of human gut bacteria. Analysis of minimal media growth requirements for seven B-vitamins (except B12, see below) in 30 bacterial strains revealed that 75 out of 89 experimentally determined phenotypes (85%) matched the predicted vitamin auxotrophies (Supplementary Table S3A). Approximately half of inconsistencies between predicted and experimental phenotypes can be reconciled considering the salvage of alternative metabolic precursors. Thus, five species including *Lactobacillus plantarum, Clostridium sporogenes, Clostridium tyrobutyricum, Clostridium botulinum* A str. Hall, and *Veillonella parvula* required folate for growth. According to our reconstruction, all these species were assigned a special B9 phenotype variant (P^∗^), which corresponds to a conditional prototrophy in the presence (but not in the absence) of the pABA precursor (Supplementary Table S2G). Likewise, *Clostridium difficile* and *Megasphaera elsdenii* experimentally established as B5 auxotrophs were also assigned a conditional B prototrophy (P^∗^) phenotype (Supplementary Table S2F) due to the unknown source of β-alanine, that in the case of these two species, apparently has to be salvaged exogenous sources. The remaining inconsistencies (∼8%) may originate from a variety of factors including strain-specific phenotype variations (see the next section).

In contrast to other B-vitamins, B12 is not essential for growth of some bacterial strains that either lack B12-dependent enzymes or possess alternative B12-independent enzymes that can substitute their B12-dependent functional analogs (Rodionov et al., 2003). Indeed, the analysis of published experimental data confirms that B12 is not essential for growth of 17 auxotrophic species (Supplementary Table S3B). These include 9 *Staphylococcus* spp. that lack any B12-dependent enzyme and *Bacillus cereus* that has a single B12-dependent enzyme, the methionine synthase MetH, and its B12-independent analog MetE (Zhang et al., 2009). In contrast, vitamin B12 is required for growth of 20 other species, most of which lack *de novo* B12 biosynthetic genes (Supplementary Table S3B). However, six B12-requiring strains including four species from the *Bacteroidales* order, *Collinsella aerofaciens* and *Clostridium scindens* possess a complete set of B12 synthesis genes. One plausible explanation of these inconsistent B12 phenotypes might be the absence of upstream genes required for the biosynthesis of preccorin-2, a common precursor of B12 and heme (Roper et al., 2000), that were not included in our B12 phenotype analysis. Such species might be classified as preccorin-2 (rather than B12) auxotrophs.

We further analyzed experimentally characterized B-vitamin production capabilities of 24 members of the gut microbiota (Supplementary Table S3C). All 50 experimentally established phenotypes are fully consistent with the predicted vitamin prototrophy phenotypes. Interestingly, all members of the *Lactobacillus* genus as well as two *bifidobacteria* are able to synthesize B9 only in the presence of pABA precursor, which is in agreement with the absence of pABA biosynthesis genes in these genomes. Overall, the examined experimental data on B-vitamin requirements and production are in good agreement with our *in silico* reconstruction and prediction of B-vitamin phenotypes.

### Binary Vitamin Phenotypes: Phylogenetic Distribution and Variability

To enable a computational microbiome-wide phenotype assessment and comparison between different HGM samples, we have introduced a concept of *digital binary phenotypes* where a numeric value of a particular vitamin phenotype in a reference genome can be either “1” (prototrophy) or “0” (auxotrophy). For the purpose of this analysis, we have converted all the detailed variant codes (Supplementary Table S2) to a simplified binary form assigning the value of “1” to all P-variants and “0” to all A-variants. The obtained values for each of the nine phenotypes across 2,228 reference HGM genomes were combined into a Binary Phenotype Matrix (BPM) (Supplementary Table S1). This simplified representation allows us to address several important questions.

**FIGURE 3:**
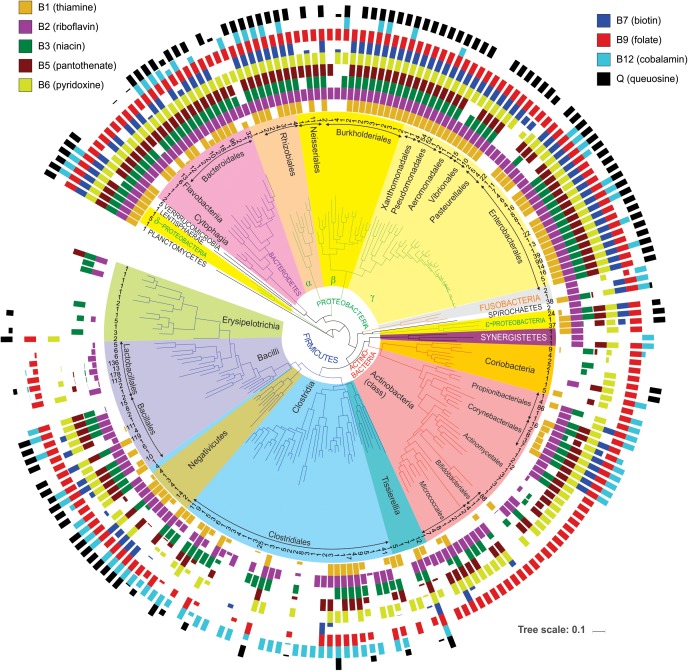
Distribution of vitamin producers among analyzed HGM strains. The phylogenetic tree of HGM genera was obtained from the larger tree constructed by RAxML based on concatenated sequences of ribosomal proteins from the analyzed HGM species. Number of analyzed strains per genus is shown in the inner circle; higher-level taxonomic groups such as orders, classes, and phyla are highlighted inside the tree. Colored bars show average vitamin production phenotypes (prototrophy) of each genus. Empty bars correspond to auxotrophic phenotypes.

First, it provides a simple way to assess a phylogenetic distribution of micronutrient requirements and production capabilities across a broad range of HGM genomes. For this purpose, we used BPM to calculate *averaged vitamin prototrophy phenotype values* at various taxonomic ranks: species, genus, family, order, class, and phylum (Supplementary Table S4). These averaged values vary on the scale from 0 to 1 (0–100%) providing an estimate of probability (frequency) of an organism in a group to be a prototroph. The obtained distribution of predicted vitamin prototrophy across 690 species was visualized on the phylogenetic tree of HGM species for 230 genera (Figure 3), also the percentages of vitamin producers were calculated for each analyzed phylum (Supplementary Figure S3). The Proteobacteria and Bacteroidetes phyla contain mostly prototrophs that are capable of synthesizing all vitamins, excluding cobalamin (B12), which is synthesized only by 30 and 42% representatives of these phyla in the reference collection, respectively. The majority of representatives of the Fusobacteria phylum (except *Cetobacterium* and *Leptotrichia*) are capable of synthesizing all vitamins except pantothenate (B5) and queuosine. The Verrucomicrobia phylum represented by five *Akkermansia* strains, as well as a single HGM strain from the Planctomycetes phylum, have biosynthetic capabilities for all vitamins except B12. The Lentisphaerae phylum represented by a single HGM species, *Victivalis vadensis*, produces all vitamins except B5. Both HGM species from the Spirochaetes phylum are auxotrophic for all vitamins except riboflavin (B2). The *Ureaplasma* and *Mycoplasma* strains from the Tenericutes phylum are auxotrophic for all nine micronutrients. The Firmicutes, Actinobacteria and Synergistetes phyla contain the largest number of auxotrophs for the majority of vitamins. Among Actinobacteria, vitamin B12 is synthesized by species from three orders (Propionibacterales, Corynebacterales, Coriobacteriales), whereas among Firmicutes the majority of B12 producers belong to the Clostridiales, Selenomonadales and Veillonellales orders. Finally, queuosine is not synthesized by most Actinobacteria except two Coriobacteria species, whereas among Firmicutes it is mostly synthesized by Bacillales and Negativicutes. Overall, this analysis reveals general correlational trends between phylogeny (even at higher taxonomic ranks) and vitamin phenotypes, but also points to substantial phenotype variations as visualized by fractional averaged phenotype values (between 0 and 1).

Estimating intraspecies as well as interspecies variations of binary vitamin phenotypes is essential to assess boundaries of confident phylogeny-based phenotype projection for the purpose of microbiome-wide predictive phenotype profiling of HGM samples (see next section). To assess these variations within species or at higher taxonomic levels we used two metrics: (i) *number of variable phenotypes* (NVP, ranges from 0 to 9 for nine phenotypes); and (ii) *overall phenotype variability score* calculated as a sum of variances for each vitamin phenotype (OPVS, ranges between 0 and 4.5 for nine phenotypes) (Supplementary Table S4A). The highest individual vitamin variability score 0.5 corresponds to a case when a species is represented by an equal number of vitamin auxotrophic and prototrophic strains.

**FIGURE 4:**
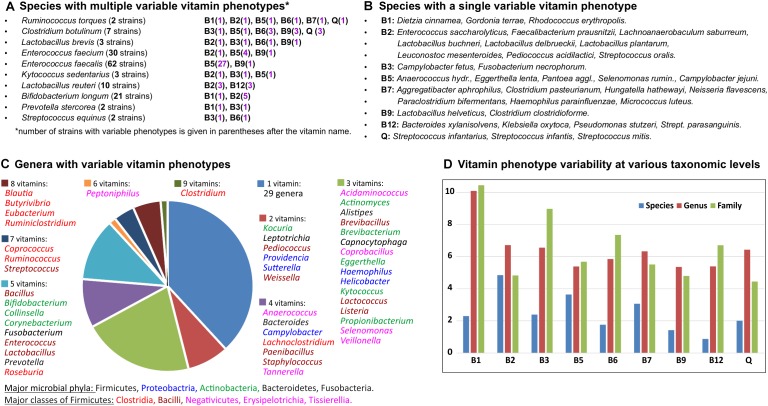
Inter-and intra-species variability of binary vitamin production phenotypes in HGM genomes. **(A)** Species with multiple variable vitamin phenotypes. **(B)** Species with a single variable vitamin phenotype. **(C)** Genera with variable vitamin phenotypes. **(D)** Vitamin phenotype variability at various taxonomic levels.

Overall, 282 out of 694 analyzed HGM species are represented by more than one strain, including 34 species with 10 or more strains (Supplementary Table S1). Of those, 45 species (∼15%) have at least one variable vitamin phenotype (Figure 4A,B) including 10 species characterized by more than one variable phenotype (NVP > 1). *Ruminococcus torques* represented by two strains in the analyzed set of genomes shows the highest variability of vitamin phenotypes (NVP = 6; OPVS = 3), suggesting that these strains could be incorrectly classified. Indeed, based on phenotypic, biochemical, phylogenetic and genomic evidence one of these strains has recently been reclassified as *Mediterraneibacter torques* ATCC 27756 (Togo et al., 2018). From an evolutionary perspective, the observed cases of intraspecies variability of vitamin phenotypes can be explained by either loss or acquisition of vitamin biosynthesis genes, which are often co-localized into gene clusters on the chromosome. For example, comparative analysis of 62 strains of *Enterococcus faecalis* revealed that 27 strains presumably have lost the *panDBGC* gene locus encoding all essential enzymes from the *de novo* pantothenate biosynthesis pathway (Supplementary Table S2). Four out of five strains of *Faecalibacterium prausnitzii* and three out of five strains of *Lactobacillus reuteri* possess the riboflavin biosynthesis operon *ribADHBE*, while their closely related strains apparently have lost the B2 biosynthetic gene clusters. In contrast, one out of five strains of *Streptococcus parasanguinis* likely has acquired a contiguous gene locus encoding 20 enzymes required for B12 biosynthesis (Supplementary Table S2).

The same metrics were used to assess interspecies variations and variations at higher taxonomic levels (Supplementary Table S4). At the genus level, 139 out of 230 analyzed genera are represented by more than one species, and 76 of them (55%) demonstrated various degrees of phenotype variability. Genera with the highest NVP and OPVS values belong to the Clostridia, Bacilli and Tissierellia classes from the Firmicutes phylum, as well as some genera from the Actinobacteria and Proteobacteria phyla (Figure 4C). Variability of vitamin phenotypes gradually increases at higher taxonomic ranks. At the family level, 82 out of 96 families are represented by more than one genus, and 61 of them (74%) exhibit various degrees of phenotype variability. Interestingly, the top four families with the greatest variability scores, namely *Eubacteriaceae, Ruminococcaceae, Clostridiaceae*, and *Lachnospiraceae*, belong to the Clostridia class further emphasizing its contribution to vitamin phenotype variability in the gut microbiome. At the order level, 30 out of 36 taxa represented by more than one genome (83%) demonstrate highly variable vitamin phenotypes. At the phylum and class levels, Actinobacteria and Firmicutes (including Clostridia, Bacilli and Tissierellia) demonstrate the highest degree of phenotype variability (OPVS > 2) with 8 and 9 variable phenotypes, respectively.

We also calculated vitamin-specific variability scores that take into account variability of phenotypes in all taxa at a given taxonomic rank (Figure 4D). At the species level, biosynthesis of vitamin B2 (riboflavin) and B12 (cobalamin) are the least and most variable phenotypes, respectively. Vitamin B1 (thiamine) shows the highest variability scores both at the genus and family levels.

Based on this analysis, we conclude that an accurate phylogeny-based vitamin phenotype projection from reference genomes to OTUs or phylotypes mapped by 16S rRNA gene profiling of HGM samples, is generally impossible at the taxonomic level higher than a family. Moreover, the projection at lower levels, of families, genera and even species may be affected by varying phenotype heterogeneity within respective groups. Therefore, to optimize the accuracy and coverage of microbiome-wide phenotype predictions, we have to account for phenotype heterogeneity characteristic of each phylotype/OTU (see next section).

### Microbiome-Wide Predictive Vitamin/Cofactor Phenotype Profiling

We applied the obtained BPM that captures vitamin/cofactor production capabilities and requirements across a collection of curated reference genomes in a simplified digital form for prediction of community-wide phenotypes from HGM 16S samples profiling data. This analysis is performed in two stages, each enabling a particular type of community phenotype profiling (Supplementary Figure S2B).

The goal of the *first stage* is to tentatively assign prototrophy/auxotrophy phenotypes to all OTUs (or phylotypes) identified in a sample (above a certain abundance threshold) that are subsequently confidently mapped to our reference collection at least at the family level. As discussed in the previous section, unlike the case of reference genomes, some of these assignments (especially those mapped at higher phylogenetic level) are bound to have fractional values (between 0 and 1). In the current implementation of the Phenotype Profiler pipeline (by Phenobiome Inc.), this is achieved by OTU taxonomic assignment mapping to reference genomes using a hierarchical averaging approach (see Materials and Methods for computational details). A final product at this stage of the analysis is a Community Phenotype Matrix (CPM) containing nine columns with respective weighted average phenotype values computed for all mapped OTUs. Similar to BPM, each line in CPM is a nine-digit string reflecting a metabolic potential, biosynthetic capabilities (prototrophy) vs. micronutrient requirements (auxotrophy = 1-prototrophy) of each OTU.

The main product of the *second stage* is a value, which is obtained via transformation of the OTU-abundance-phenotype table into a string of nine values reflecting fractional representation (%) of nine prototrophy phenotypes in the analyzed HGM sample. For each phenotype, this value, termed CPI, is computed as a sum of probabilistic phenotypes in each of the nine columns weighted (multiplied) by relative abundance of respective OTUs. A nine-value CPI string reflects the vitamin prototrophy/auxotrophy phenotype representation extended from an individual OTU to the entire community. Using CPI strings as simplified microbiome-wide metrics of vitamin/cofactor metabolic capabilities and requirements, enables a comparative analysis of multiple samples in model studies [as applied in the accompanying publication (Sharma et al., in review)] or across large datasets, as illustrated here by the analysis of HMP and AGP collections.

**FIGURE 5:**
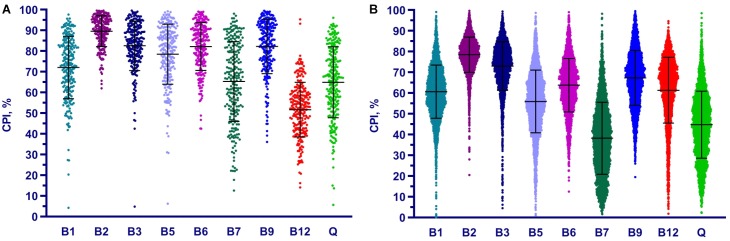
Distribution of Community Phenotype Indices for B and Q vitamins in HGM samples from HMP **(A)** and AGP **(B)** datasets. CPIs are calculated based on taxonomic assignments before 16S count renormalization.

We applied the Phenotype Profiler pipeline to the comparative analysis of two large HGM 16S rRNA datasets from the HMP and AGP projects containing 245 and 2,863 samples, respectively. Both datasets were pre-processed using the QIIME2 pipeline and the obtained OTUs were taxonomically assigned using our custom heuristic-based procedure (see Materials and Methods). The OTU-abundance values were further renormalized to take into account substantial variations in 16S rRNA gene copy number between different species of HGM bacteria (Supplementary Table S5 and Supplementary Figure S4). CPI values and respective prediction errors were calculated for each vitamin (B1, B2, B3, B5, B6, B7, B9, B12, and Q) and each sample from the HMP and AGP datasets using both, original phylogenetic profiles and renormalized by 16S rRNA gene copy number (Supplementary Table S6). The prediction errors for individual CPI values were in the range 1–7% for HMP and 2–5% for AGP showing only a moderate impact of imprecise phenotype mapping and phenotype microheterogeneity. The distribution of CPI values for B-vitamins and Q across all samples in the two analyzed datasets is illustrated here for the original taxonomic profiles (Figure 5). The impact of renormalization by 16S rRNA gene copy number is shown in a Supplementary Figure S5. In both datasets, CPI values are significantly variable between samples suggesting distinct micronutrient requirements of the respective gut communities. Although the obtained vitamin phenotype profiles for HMP and AGP datasets differ substantially we also noted common trends for certain vitamins (see Figure 5 and Table 2 for mean CPI values). Thus, both datasets show the highest mean CPI values for B2, B3, B6, and B9. The HMP dataset is characterized by the lowest mean CPI value for B12. In contrast, vitamin B7 and Q demonstrate the lowest average CPIs in the AGP dataset.

**Table 2:** Mean CPI values for B-vitamins and Q in HMP and AGP datasets before and after renormalization of taxonomic profiles by 16S gene counts.

Dataset	B1	B2	B3	B5	B6	B7	B9	B12	Q
HMP (original)	72.1	89.7	82.6	78.5	82.2	65.3	82.3	51.7	64.9
HMP (renormalized)	66.4	90.4	81.8	79.1	81.8	59.9	82.2	43.9	58.2
AGP (original)	60.7	78.6	73.1	56.0	63.9	38.3	67.4	61.4	44.8
AGP (renormalized)	58.9	78.9	73.7	57.9	65.4	35.6	68.4	56.8	42.4


Overall, the phenotype profiling approach proposed in this study complements the established taxonomic profiling approach commonly used for description and comparative analysis of microbial communities. Thus, vitamin-specific CPIs provide useful metrics to compare and contrast micronutrient requirements of HGM communities, while the community-wide distribution of phenotype strings points to major vitamin acceptors and potential donors in these communities.

### Lifestyle Preferences in the Human Gut Microbial Communities

A more granular analysis of phenotype profiles of numerous HGM samples allowed us to implicate specific taxa providing major contributions to predominantly prototrophic vs. auxotrophic phenotypes in gut microbial communities. To compare fitness (by relative abundance) of various combinations of vitamin phenotypes in individual species from HGM samples representing different “lifestyles” (e.g., multi-prototrophy vs. multi-auxotrophy lifestyles), we calculated relative contribution of ordered nine-digit strings of individual binary phenotypes (as described in Materials and Methods). As a first approximation of such lifestyles, we introduced a simple metric, *vitamin prototrophy rank* (VPR), ranging from 0 (complete auxotrophy) to 9 (complete prototrophy) with respect to all nine analyzed micronutrients. Intermediate VPR values, 1 through 8, are assigned to groups of species showing prototrophy with respect to 1 through 8 (out of 9) micronutrients. For each sample in the analyzed HMP and AGP communities, we calculated two values: (i) the observed VPR frequencies (*P*_obs_), and (ii) the expected VPR probabilities obtained in the assumption of independence of individual phenotypes in each organism (*P*_exp_) (Supplementary Figure S6).

**FIGURE 6:**
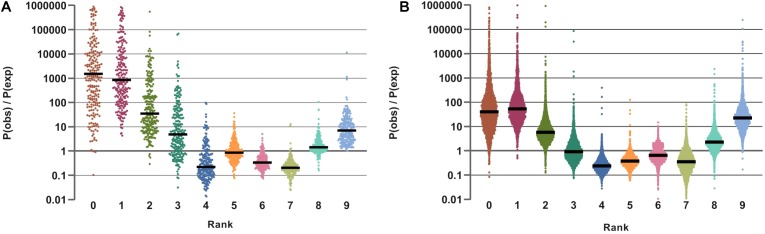
Distribution of ratio of observed and expected probabilities for Vitamin Prototrophy Ranks in HGM samples from HMP **(A)** and AGP **(B)** datasets. Distributions are presented on a logarithmic scale.

The results of this analysis are illustrated by ratios of the observed over expected frequency of nine aggregated lifestyles across all samples in HMP and AGP datasets (Figure 6). Despite differences in absolute frequencies of distinct VPRs (such as a clear prevalence of multi-prototrophs with VPR = 8 or 9, see Supplementary Figure S6A), the comparison of relative frequencies (*P*_obs_/*P*_exp_) reveals enrichment for both extreme lifestyles, multi-prototrophy and multi-auxotrophy (VPR ≤ 3). This trend, which is observed in both datasets, may reflect fundamental properties of organization of syntrophic microbial communities where species with an extreme “parasitic” lifestyle are not only tolerated but gain certain selective advantages as long as multi-prototrophic donors of micronutrients are sufficiently abundant. Major genera contributing to the extreme prototrophic, auxotrophic and intermediate lifestyles are enlisted in Table 3. The *Bacteroides* spp. are major contributors to a multi-prototrophic lifestyle in both HMP and AGP datasets. Among major multi-auxotrophs, *Faecalibacterium* spp. are common in both datasets representing the most successful B-vitamin acceptors in microbial gut communities. It is tempting to consider major taxa representing multi-prototrophic lifestyles as potential vitamin donors enabling sustainability of numerous auxotrophic acceptors. Identity of such donors and genomic features distinguishing *sharing* from *non-sharing* prototrophs (e.g., a particular class of efflux transporters or/and regulators) remain an important direction of future research, which would impact our understanding of metabolic interactions in microbial communities.

**Table 3:** Taxonomic genera mostly contributing to VPR ranks in HGM samples from the HMP and AGP datasets.

Ranks	HMP^∗^	AGP^∗^
7–9	*Bacteroides* (61%), *Alistipes* (15%), *Parabacteroides* (5%), *Paraprevotella* (3%)	*Bacteroides* (44%), *Ruminococcus* (8%), *Akkermansia* (5%), *Anaerostipes* (4%) *Parabacteroides* (4%)
4–6	*Alistipes* (28%), *Blautia* (10%), *Roseburia* (8%), *Eubacterium* (7%), *Prevotella* (5%)	Alistipes (16%), *Roseburia* (14%), *Blautia* (13%), *Prevotella* (9%), *Bifidobacterium* (7%)
0–3	*Faecalibacterium* (17%), *Oscillibacter* (13%), *Lactobacillus* (8%), *Parasutterella* (5%)	*Faecalibacterium* (22%), *Lachnoclostridium* (7%), *Oscillospira* (5%), *Lactobacillus* (4%)


*^∗^Average contribution of a genus to a subset of VPR ranks (by abundance) is given in parentheses.*

### Comparison of Phenotype Profiling and Pathway Abundance Approach

**FIGURE 7:**
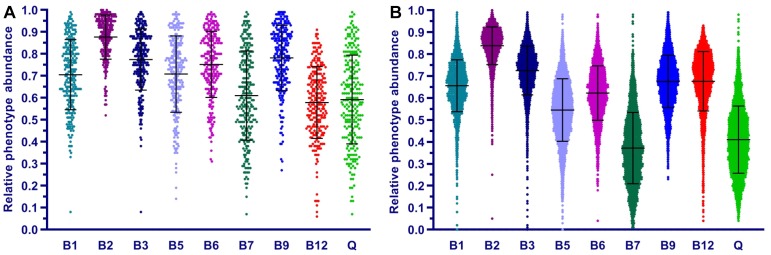
Distribution of relative phenotype abundance for B and Q vitamins in HGM samples from HMP **(A)** and AGP **(B)** datasets. Relative phenotype abundances are calculated using PICRUSt based on external traits from BPM for 2,228 reference genomes obtained in this study.

Of several approaches to functional description of microbial communities, a combination of PICRUSt [an ancestral-state reconstruction algorithm (Langille et al., 2013)] with the MinPath tool (Ye and Doak, 2009) and MetaCyc pathways collection (Caspi et al., 2012) implemented in the PICRUSt2 pipeline enables a similar analysis. Indeed, this analysis yields pathway abundances, which, at least for the case of eight B-vitamins, may be directly compared to respective CPI values. For the purpose of such comparison, we used our BPM for 2,228 reference HGM genomes as an input trait table to predict *vitamin phenotype abundances* in HGM samples from HMP and AGP datasets by using PICRUSt algorithm (Supplementary Table S7). The obtained PICRUSt-based relative phenotype abundances (termed this way by analogy with pathway abundances) are essentially equivalent to CPIs, and their distributions show similar trends (compare Figure 7 and Figure 5). Thus, we consider a combination of BPM with PICRUSt-based phenotype abundances as a potential alternative approach to community phenotype profiling.

We further compared the PICRUSt-based phenotype abundance profiles with vitamin biosynthesis pathway abundances computed for the same HMP and AGP metagenomic datasets using the default PICRUSt2/MinPath approach (see Materials and Methods). We selected a subset of metabolic pathways in MetaCyc database that most closely describe the reconstructed in this work *de novo* vitamin biosynthetic capabilities. For B3, B7, and B12 we included in the analysis two alternative biosynthetic pathways captured in MetaCyc, and their abundances were summed up to get cumulative values for each vitamin (Table 4). The obtained MinPath/MetaCyc pathway abundances (Supplementary Table S7) were compared with the BPM-based phenotype abundances for each vitamin pathway (except B12) and dataset (Supplementary Figure S7). For most compared B-vitamins, the observed correlation coefficients exceeded 0.75 (with the exception of B12, where the predicted MinPath/MetaCyc-based pathway abundances of both anaerobic and aerobic pathways were too low reflecting some technical problem, and vitamin B6, see below). The best correlation was observed for B3, B5, and B9 in both HMP and AGP datasets (Table 4). A weaker correlation for vitamin B6 is potentially explained by the absence of the alternative pathway 2 variant in MetaCyc database, while according to our analysis this pathway is very common among HGM bacteria. Likewise, a relatively weak correlation for vitamin B1 can be explained by the absence in MetaCyc collection of the alternative pathway 2 for biosynthesis of the HET moiety.

**Table 4:** Correlation coefficients for comparison of phenotype abundances produced by PICRUSt2 with binary phenotype and MetaCyc pipelines.

Vitamin	AGP	HMP	MetaCyc pathway(s)
B1	0.62	0.88	Superpathway of thiamin diphosphate biosynthesis I
B2	0.79	0.87	Flavin biosynthesis I (bacteria and plants)
B3	0.84	0.89	NAD biosynthesis I (from aspartate); NAD biosynthesis II (from tryptophan)
B5	0.86	0.94	Pantothenate and coenzyme A biosynthesis I
B6	0.57	0.62	Pyridoxal 5^′^-phosphate biosynthesis I
B7	0.82	0.76	Biotin biosynthesis I; biotin biosynthesis II
B9	0.89	0.93	Superpathway of tetrahydrofolate biosynthesis
B12^∗^	–	–	Adenosylcobalamin biosynthesis II (late cobalt incorporation); adenosylcobalamin biosynthesis I (early cobalt insertion)
Q	0.78	0.91	preQ0 biosynthesis


*^∗^Both anaerobic (early cobalt insertion) and aerobic (late cobalt incorporation) pathways of B12 biosynthesis received very low or zero abundances in most analyzed HGM samples using MinPath/MetaCyc approach, thus we did not compare them with predicted phenotype abundances for vitamin B12.*

## Conclusion and Future Perspectives

By applying the subsystem-based genomic reconstruction, we have analyzed pathways for biosynthesis, salvage and uptake of eight B-group vitamins (and queuosine) for HGM bacteria represented by a diverse reference set of 2,228 genomes. Overall, nine reconstructed metabolic subsystems include over two hundred functional roles encoded by distinct protein families (Figure 1 and Supplementary Figure S1). Specific combinations of the inferred components of biosynthetic pathways and transporters for vitamins and precursors provide genomic signatures, which allowed us to classify all organisms in the collection with respect to their biosynthetic and uptake capabilities (pathway variants) and predict their prototrophic vs. auxotrophic phenotypes (Table 1). The obtained results further supported the importance of vitamin cross-feeding and pointed to specific families of transporters potentially contributing to this type of metabolic interactions in HGM communities (Figure 2). We detected subsets of auxotrophs encoding partially truncated pathway variants implicating some precursors and derivatives of canonical vitamins (such as thiazole, quinolinate, dethiobiotin, and pantoate) as alternative vitamers potentially broadening the vitamin exchange “market” in HGM consortia (Table 1). Robustness of the predicted nutritional requirements and vitamin production capabilities is supported by the observed consistency between the *in silico* phenotype predictions and published experimental data.

To enable quantitative comparative analysis of community-wide phenotype profiles, we have converted all detailed pathway variant codes to a simplified form of *digital binary phenotypes* with 1 or 0 values corresponding to prototrophy and auxotrophy, respectively. These values computed for each of the nine vitamin phenotypes across 2,228 HGM genomes capture our reference collection in a compact form of a Binary Phenotype Matrix (BPM). Overall, auxotrophic phenotypes are very common in HGM species, and only a small subset of microorganisms can synthesize all vitamins further supporting the micronutrient sharing hypothesis (Figure 3). The analysis of phylogenetic distribution of phenotypes within the entire BPM reveals substantial intraspecies and interspecies variations of micronutrient requirements and production capabilities (Figure 4). It also allowed us to establish taxonomic boundaries of phenotype conservation, which is essential for phylogeny-based projection of phenotypes from reference genomes to a variety of phylotypes (OTUs) comprising HGM samples. This analysis shows that most vitamin phenotypes are largely conserved at the level of species although some variations between strains and isolates are observed and need to be accounted for by phenotype projection tools. While the phenotype heterogeneity is gradually increasing at higher taxonomic levels, reasonably accurate probabilistic phenotype prediction (weighted for the observed frequency of alternative phenotypes within given taxa) is still feasible at the genus and, to a lesser extent, at the family (but not higher) level. Quite obviously, a well-anticipated growth in the number of sequenced and analyzed reference genomes as well as the increased accuracy of phylogenetic mapping (e.g., by using longer 16S rRNA gene sequences or/and additional phylogenetic signatures) will improve accuracy of phylogeny-based phenotype assignments and provide better estimates of prediction confidence.

Combining the BPM with phylogenetic profiles of HGM samples (e.g., obtained by 16S profiling) enables a new computational approach to *in silico* phylotype-to-phenotype predictive profiling. Application of this approach to the analysis of a broad range of HGM samples from HMP and AGP data sets yielded a comprehensive coverage (in most cases > 95% by relative abundance of mapped OTUs) and high confidence (estimated average error < 10%) of microbiome-wide phenotype predictions. The output of this analysis, in the simplest aggregated form of Community Phenotype Indexes (CPIs), provides an estimate for a fractional representation (relative abundance, from 0 to 100%) of auxotrophy vs. prototrophy for all nine analyzed phenotypes in each analyzed HGM sample. Substantial variations of CPI values are observed between individual samples as well as between these two data sets (Figure 5) potentially reflecting some technical differences between HMP and AGP sample and data acquisition pipelines. Indeed, we have compared the average Shannon alpha diversities between HMP and AGP datasets (Supplementary Table S6) and found that the HMP dataset is characterized by relatively lower diversity (4.3 for HMP vs. 5.2 for AGP, see Supplementary Figure S4C). Quite likely this difference in diversity may at least partially account for the observed phenotypic differences between these datasets. Nevertheless, some common trends are also observed providing the first estimate of micronutrient requirements (auxotrophy) as well as production/sharing (prototrophy) capabilities of HGM communities. Thus, despite the aforementioned high frequency of auxotrophic phenotypes in reference genomes, in real-life HGM samples, the abundance-weighted prototrophy is a dominant phenotype with mean CPI values above 50–60% for most (but not all) vitamins. This analysis confirmed a substantial level of auxotrophy and, thus, a requirement for the exogenous supply of the entire set of nine analyzed micronutrients (eight B vitamins and queuosine), as a characteristic feature of HGM communities across a broad range of samples.

*Overall*, in addition to further supporting a hypothesized role of syntrophic micronutrient metabolism in HGM communities, a comparative phenotype profiling approach established in this study has provided a computational framework for the experimental testing of this hypothesis (Sharma et al., in review). This approach is extending methodology of a functional description of microbial communities, which, presently, is not as established as phylogenetic profiling methods. A side-by-side comparison of our phenotype-based approach with the most advanced existing approach, PICRUSt2, predicting pathway abundance via a combination of MinPath algorithm with MetaCyc pathways, revealed a generally good agreement (Table 4) for most (but not all) vitamin phenotypes, as well as substantial differences, which could be partially explained by the differences in the applied rules and in the extent of gene/pathway curation.

An anticipated further advancement of microbiome genomics, most importantly: (i) deeper coverage of relevant microbial communities by complete reference genomes and (ii) increased coverage and resolution of phylogenetic profiling (e.g., via better amplicon-based or shotgun metagenomics methodology), is expected to substantially improve the accuracy of predictive phenotype profiling of complex microbial communities. This approach can be further expanded via inclusion of additional digitized phenotypes covering other nutrient requirements (e.g., amino acids), utilization capabilities (e.g., carbohydrates) (Yatsunenko et al., 2012; David et al., 2014; Zhang et al., 2014; Wu et al., 2015; Blanton et al., 2016; Hibberd et al., 2017; Sheflin et al., 2017; Gehrig et al., 2019), catabolic end-products (e.g., production of short-chain fatty acids, other physiologically active metabolites) as well as some non-metabolic phenotypes (antibiotic resistance, virulence, etc.) and by taking into consideration other characteristics of metagenomic samples (diversity, advanced metadata). Future practical applications employing comparative analyses of phenotype profiles across numerous HGM samples may include diagnostics (classification and correlations with patients’ data) and prevention/treatment of disbyosis-related syndromes via rational and personalized selection of probiotics and nutritional supplements.

## Author Contributions

AO and DR conceived and designed the research project. MK and AA performed the comparative genomic analysis, reconstruction of pathways and subsystem encoding. AA compared the obtained phenotypes to experimentally published data. DR provided the quality control of the obtained data. SI, MK, and SL analyzed metabolic phenotypes in the 16S datasets. DR, AA, PN, SP, and AO wrote the manuscript. All authors read and approved the final manuscript.

## Conflict of Interest Statement

The authors declare that the research was conducted in the absence of any commercial or financial relationships that could be construed as a potential conflict of interest.

## Footnotes

1 https://hmpdacc.org/hmp/HMRGD/

2 https://www.patricbrc.org

3 http://www.theseed.org

4 www.hmpdacc.org

5 www.phenobiome.com

6 https://github.com/picrust/picrust2/wiki

## Abbreviations

BPM: Binary Phenotype Matrix
CPI: Community Phenotype Index
CPM: Community Phenotype Matrix
ECF: Energy-Coupling Factor
HGM: Human Gut microbiota/Microbiome
MFS: Major Facilitator Superfamily
NVP: Number of Variable Phenotypes
OPV: Overall Phenotype Variability score
OTU: Operational Taxonomic Unit
TF: Transcription factor
VPR: Vitamin Prototrophy Rank
