# Supplementary material for: Micronutrient Requirements and Sharing Capabilities of the Human Gut Microbiome
Source: Front Microbiol. 2019 Jun 12;10:1316. doi: 10.3389/fmicb.2019.01316 (PMC6593275; doi:10.3389/fmicb.2019.01316)
Supplement: Supplementary file 1 [file Image_1.pdf]

Figure S1. (A) Thiamin (vitamin B1) biosynthesis and salvage

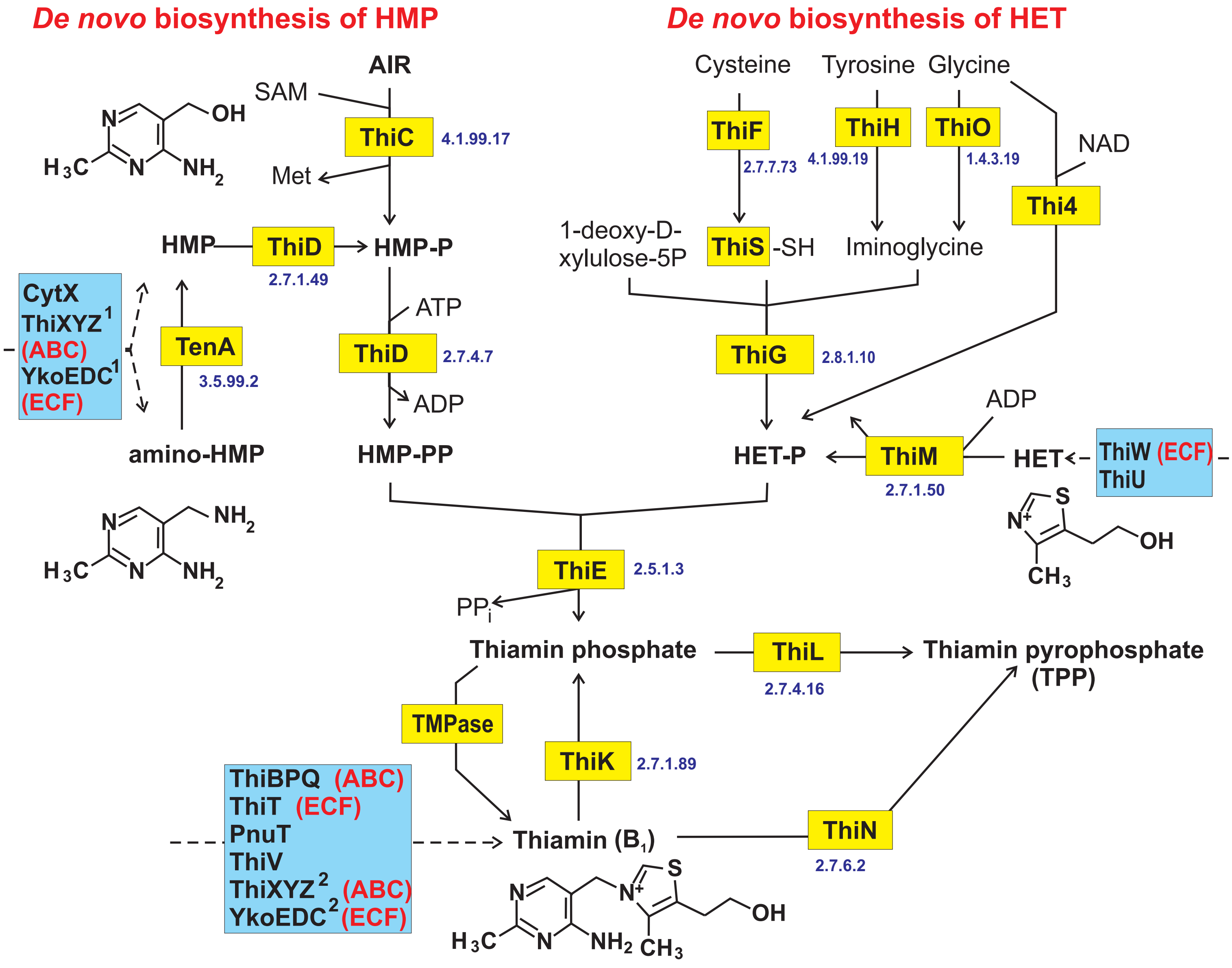

Thiamine biosynthesis / salvage enzymes

ThiS	Sulfur carrier protein ThiS
ThiF	Sulfur carrier protein ThiS adenylyltransferase (EC 2.7.7.73)
ThiH	2-iminoacetate synthase (ThiH) (EC 4.1.99.19)
ThiO	Glycine oxidase ThiO (EC 1.4.3.19)
ThiG	Thiazole synthase (EC 2.8.1.10)
Thi4	Thiazole biosynthetic enzyme Thi4
ThiC	Phosphomethylpyrimidine synthase ThiC (EC 4.1.99.17)
ThiE	Thiamin-phosphate pyrophosphorylase (EC 2.5.1.3)
ThiM	Hydroxyethylthiazole kinase (EC 2.7.1.50)
ThiD	Hydroxymethylpyrimidine phosphate kinase ThiD (EC 2.7.4.7)
ThiD2	Hypothetical alternative hydroxymethylpyrimidine phosphate kinase ThiD (EC 2.7.4.7)
TenA	Thiaminase II (EC 3.5.99.2) involved in salvage of thiamin pyrimidine moiety

Figure S1. (A) Thiamin (vitamin B1) biosynthesis and salvage

Transporters for thiamine and thiamine precursors

ThiY	Hydroxymethylpyrimidine ABC transporter, substrate -binding component
ThiX	Hydroxymethylpyrimidine ABC transporter, transmembrane component
ThiZ	Hydroxymethylpyrimidine ABC transporter, ATPase component
YkoE	Substrate-specific component YkoE of thiamin-regulated ECF transporter for HydroxyMethylPyrimidine
YkoC	Transmembrane component YkoC of energizing module of thiamin -regulated ECF transporter for HydroxyMethylPyrimidine
YkoD	ATPase component YkoD of energizing module of thiamin -regulated ECF transporter for HydroxyMethylPyrimidine
YkoDd	Duplicated ATPase component YkoD of energizing module of thiamin -regulated ECF transporter for HydroxyMethylPyrimidine
YkoF	Putative HMP/thiamine-binding protein YkoF
CytX	Predicted hydroxymethylpyrimidine transporter CytX
ThiW	Substrate-specific component ThiW of predicted thiazole ECF transporter
ThiU	Predicted thiazole transporter ThiU
ThiT	Substrate-specific component ThiT of thiamin ECF transporter
ThiB	Thiamin ABC transporter, substrate -binding component
ThiP	Thiamin ABC transporter, transmembrane component
ThiQ	Thiamin ABC transporter, ATPase component
ThiV	Predicted thiamin transporter ThiV, SSS family
PnuT	Predicted thiamin transporter PnuT
Omr1	Thiamin-regulated outer membrane receptor Omr1
ThiZ2	Thiamin ABC transporter ThiZ, ATPase component
ThiY2	Thiamin ABC transporter ThiY, substrate -binding component
ThiX2	Thiamin ABC transporter ThiX, transmembrane component
COG0011	Cytoplasmic thiamin-binding component of thiamin ABC transporter, COG0011 family
YkoE2	Substrate-specific component YkoE of thiamin-regulated ECF transporter for Thiamin
YkoD2	ATPase component YkoD of energizing module of thiamin-regulated ECF transporter for Thiamin
YkoDd2	Duplicated ATPase component YkoD of energizing module of thiamin -regulated ECF transporter for Thiamin
YkoC2	Transmembrane component YkoC of energizing module of thiamin-regulated ECF transporter for Thiamin

Downstream thiamine metabolism enzymes

ThiN	Thiamin pyrophosphokinase (EC 2.7.6.2)
ThiL	Thiamine-monophosphate kinase (EC 2.7.4.16)
ThiK	Thiamine kinase (EC 2.7.1.89)
ThiK2	Putative choline kinase, PnuC-associated, THI-regulated
ThiK3	Putative thiamine kinase, YkoEDC-associated, THI-regulated
TMPase	ThMPase type I predicted

Abbreviations for metabolites:

AIR, Aminoimidazole ribotide;
HET, 5-(2-Hydroxyethyl)-4-methyltiazole;
HMP, 4-Amino-5-phydroxymethyl-2-methylpyrimidine

Figure S1. (B) Riboflavin (vitamin B2) biosynthesis and salvage

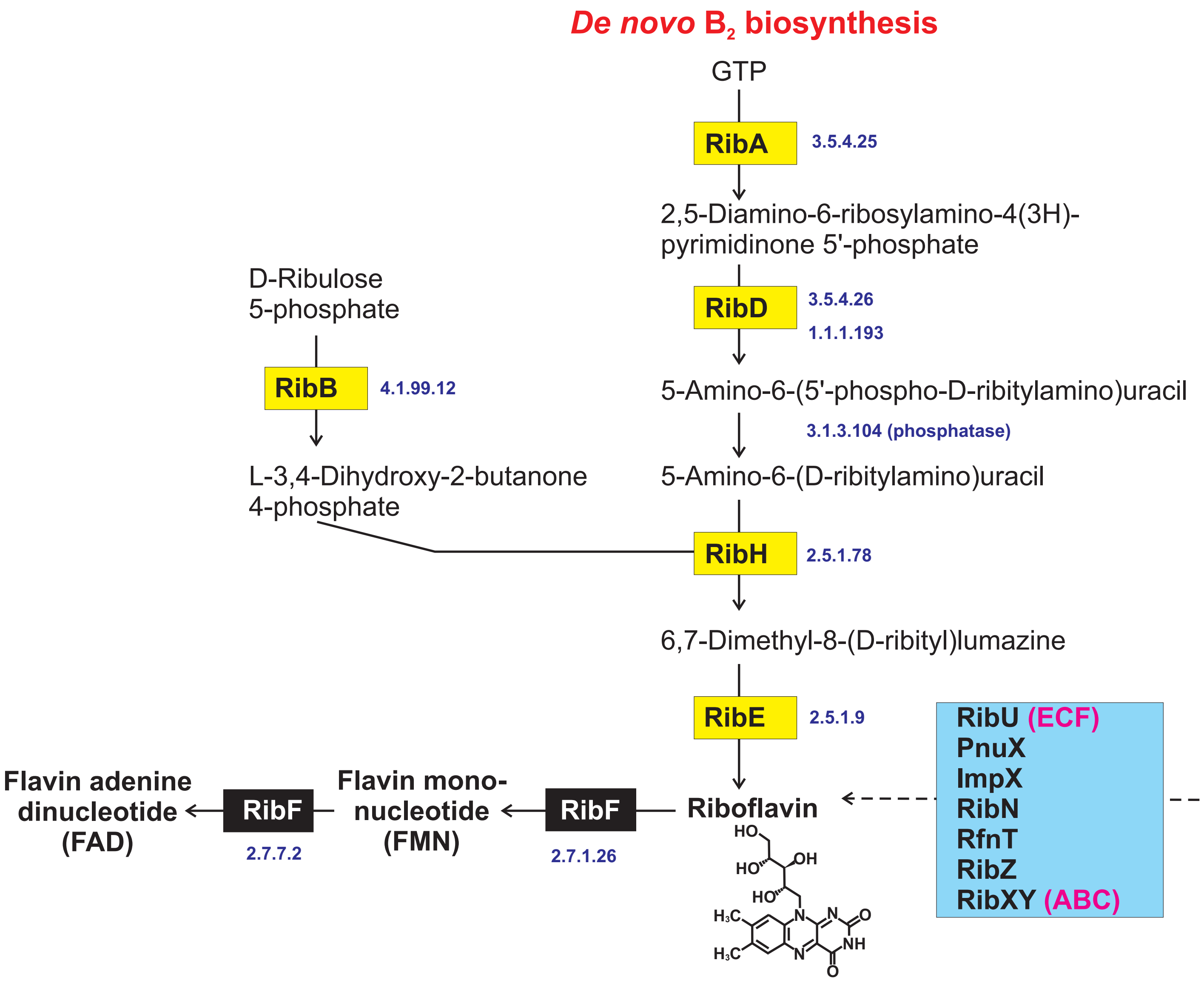

Riboflavin biosynthesis / salvage enzymes

RibA	GTP cyclohydrolase II (EC 3.5.4.25)
RibD	Diaminohydroxyphosphoribosylaminopyrimidine deaminase (EC 3.5.4.26) / 5-amino-6-(5-phosphoribosylamino)uracil reductase (EC 1.1.1.193)
RibH	6,7-dimethyl-8-ribityllumazine synthase (EC 2.5.1.78)
RibB	3,4-dihydroxy-2-butanone 4-phosphate synthase (EC 4.1.99.12)
RibE	Riboflavin synthase eubacterial/eukaryotic (EC 2.5.1.9)

Downstream riboflavin cofactor biosynthesis enzymes

RibF	Riboflavin kinase (EC 2.7.1.26) / / FMN adenylyltransferase (EC 2.7.7.2)

Figure S1. (B) Riboflavin (vitamin B2) biosynthesis and salvage

Transporters for riboflavin

RibU	Substrate-specific component RibU of riboflavin ECF transporter
PnuX	Riboflavin transporter PnuX
RibZ	Riboflavin transporter, COG2814 family
ImpX	Predicted riboflavin transporter, EamA family
RibX	Predicted riboflavin ABC transporter, transmembrane component
RibY	Predicted riboflavin ABC transporter, substrate -binding component
RibN	Riboflavin transporter RibN
RfnT	Riboflavin transporter RfnT, MFS family
RfuA	Riboflavin ABC transporter, periplasmic ribofl avin-binding protein RfuA
RfuB	Riboflavin ABC transporter, ATP -binding protein RfuB
RfuC	Riboflavin ABC transporter, permease protein RfuC
RfuD	Riboflavin ABC transporter, permease protein RfuD

Figure S1. (C) NAD biosynthesis and Niacin (vitamin B3) salvage

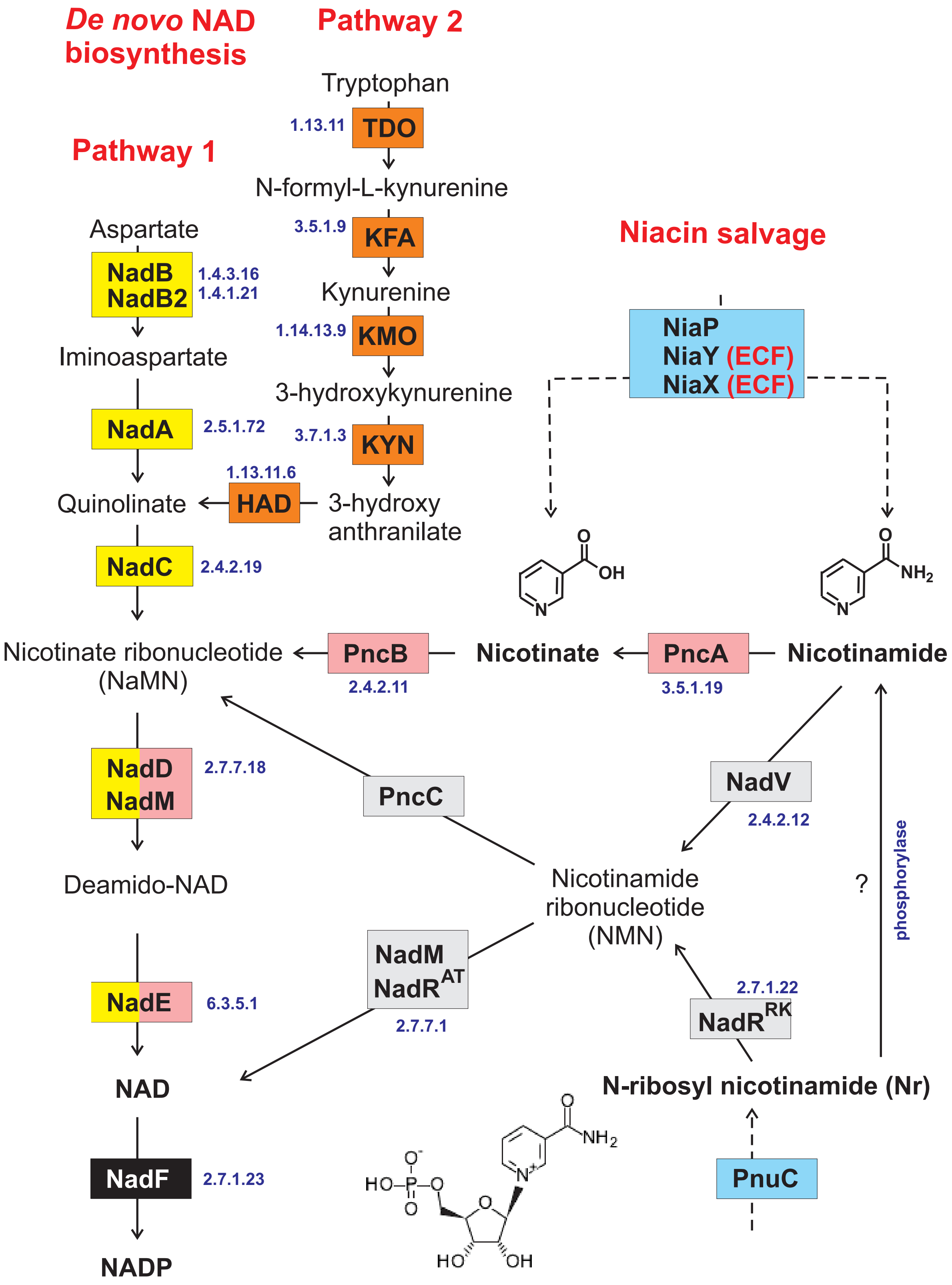

Figure S1. (C) NAD biosynthesis and Niacin (vitamin B3) salvage

NAD biosynthesis / Niacin salvage enzymes

NadB	L-aspartate oxidase (EC 1.4.3.16)
NadB2	L-Aspartate dehydrogenase (EC 1.4.1.21)
NadA	Quinolinate synthetase (EC 2.5.1.72)
NadC	Quinolinate phosphoribosyltransferase [decarboxylating] (EC 2.4.2.19)
NadD	Nicotinate-nucleotide adenylyltransferase (EC 2.7.7.18)
NadE	NAD synthetase (EC 6.3.1.5)
PncB1	Nicotinate phosphoribosyltransferase (EC 2.4.2.11)
PncB2	Nicotinate phosphoribosyltransferase (EC 6.3.4.21)
PncA	Nicotinamidase (EC 3.5.1.19)
NadV	Nicotinamide phosphoribosyltransferase (EC 2.4.2.12)
NadM	Nicotinamide phosphoribosyltransferase (EC 2.4.2.12)
NadR(ad)	Nicotinamide-nucleotide adenylyltransferase, NadR family (EC 2.7.7.1)
NadR(kin)	Ribosylnicotinamide kinase (EC 2.7.1.22)
TDO	Tryptophan 2,3-dioxygenase (EC 1.13.11.11)
KFA	Kynurenine formamidase, bacterial (EC 3.5.1.9)
KMO	Kynurenine 3-monooxygenase (EC 1.14.13.9)
KYN	Kynureninase (EC 3.7.1.3)
HAD	3-hydroxyanthranilate 3,4-dioxygenase (EC 1.13.11.6)

Transporters for Niacin and NAD precursors

NiaP	Niacin transporter NiaP
NiaX	Substrate-specific component NiaX of predicted niacin ECF transporter
NiaY	Predicted nicotinate-regulated transporter BH3254
PnuC	Ribosyl nicotinamide transporter, PnuC-like

Transcriptional regulators of Niacin and NAD metabolism genes

NrtR	Nudix-related transcriptional regulator NrtR
NiaR	Transcriptional repressor for NAD biosynthesis in gram-positives

Figure S1. (D) Pantothenate (vitamin B5) biosynthesis and salvage

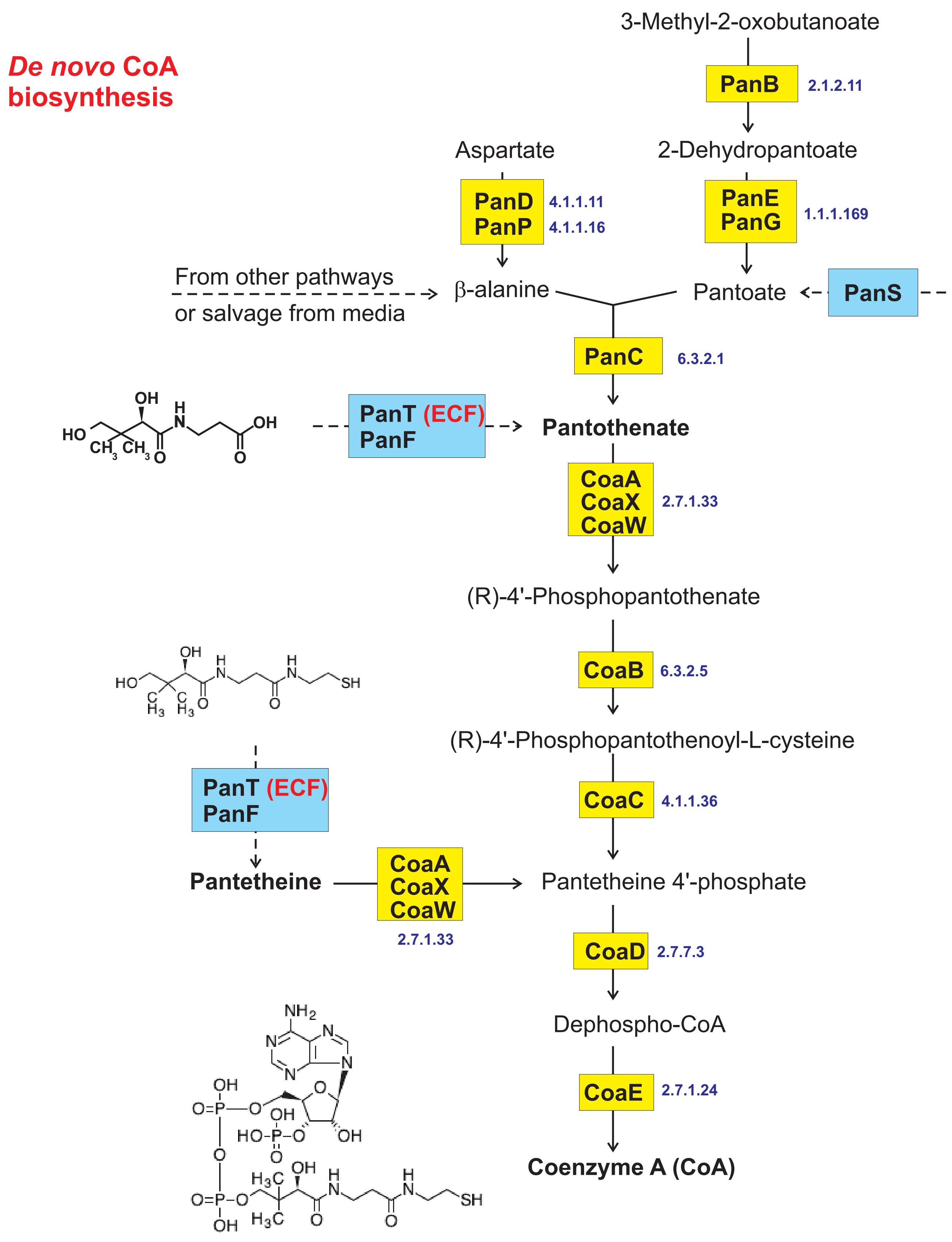

Figure S1. (D) Pantothenate (vitamin B5) biosynthesis and salvage

Pantothenate / CoA biosynthesis enzymes

PanD	Aspartate 1-decarboxylase (EC 4.1.1.11)
PanP	PLP-dependent aspartate 1-decarboxylase, PanP (EC 4.1.1.15)
PanB	3-methyl-2-oxobutanoate hydroxymethyltransferase (EC 2.1.2.11)
PanG	Ketopantoate reductase PanG (EC 1.1.1.169)
PanE	2-dehydropantoate 2-reductase (EC 1.1.1.169)
IlvC	Ketol-acid reductoisomerase (EC 1.1.1.86)
PanC	Pantoate--beta-alanine ligase (EC 6.3.2.1)
CoaA	Pantothenate kinase (EC 2.7.1.33)
CoaX	Pantothenate kinase type III, CoaX-like (EC 2.7.1.33)
CoaW	Pantothenate kinase type II, eukaryotic (EC 2.7.1.33)
CoaB	Phosphopantothenoylcysteine synthetase (EC 6.3.2.5)
CoaC	Phosphopantothenoylcysteine decarboxylase (EC 4.1.1.36)
CoaD	Phosphopantetheine adenylyltransferase (EC 2.7.7.3)
CoaE	Dephospho-CoA kinase (EC 2.7.1.24)

Transporters for Pantothenate and CoA precursors

PanT	Substrate-specific component PanT of predicted pantothenate ECF transporter
PanF	Pantothenate:Na+ symporter (TC 2.A.21.1.1)
PanS	Pantothenate precursors transporter PanS

Figure S1. (E) Pyridoxin (vitamin B6) biosynthesis and salvage

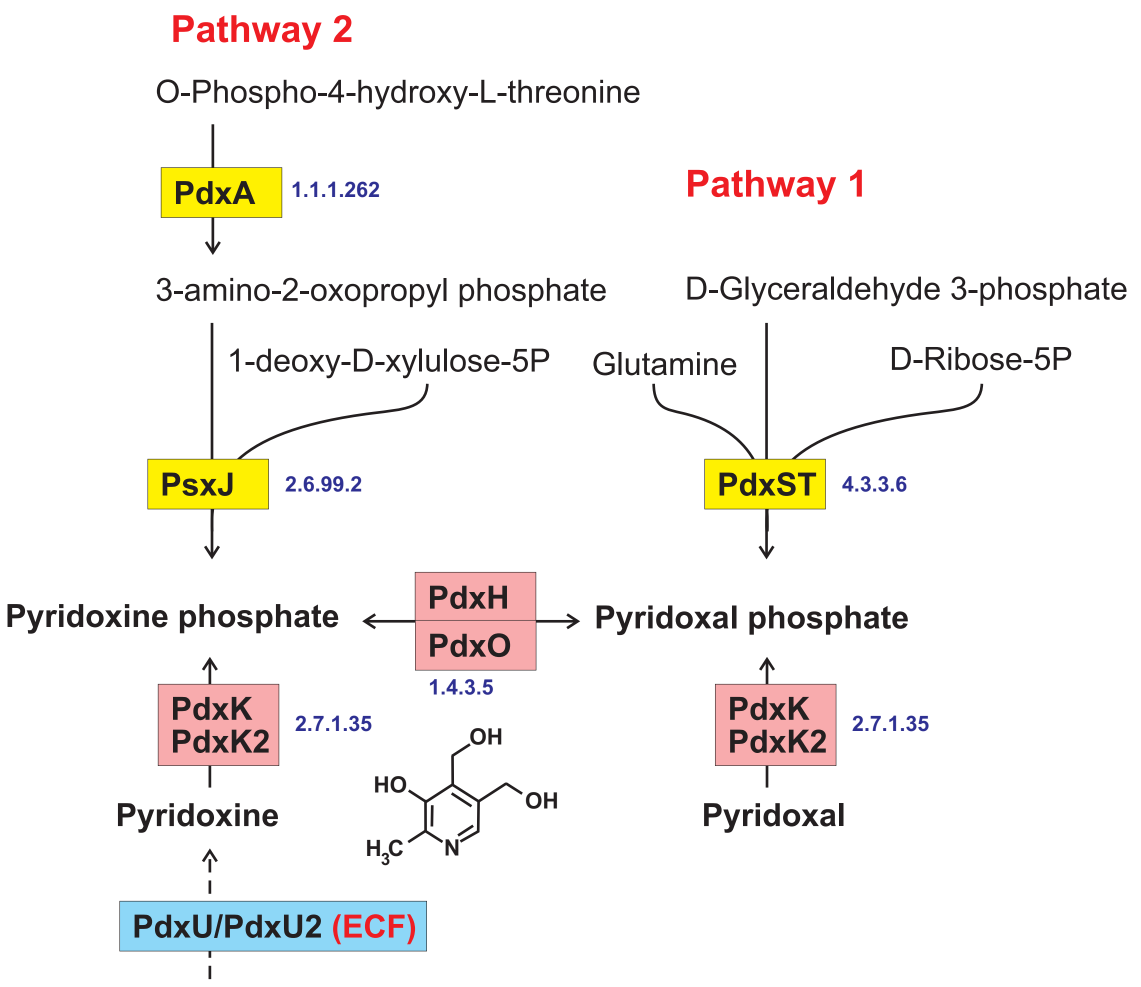

Pyridoxine / Pyridoxal phosphate biosynthesis enzymes

PdxS	Pyridoxal 5'-phosphate synthase (glutamine hydrolyzing), synthase subunit (EC 4.3.3.6)
PdxT	Pyridoxal 5'-phosphate synthase (glutamine hydrolyzing), glutaminase subunit (EC 4.3.3.6)
PdxA	4-hydroxythreonine-4-phosphate dehydrogenase (EC 1.1.1.262)
PdxJ	Pyridoxine 5'-phosphate synthase (EC 2.6.99.2)
PdxK	Pyridoxal kinase (EC 2.7.1.35)
PdxK2	Novel pyridoxal kinase, thiD family (EC 2. 7.1.35)

Pyridoxine transporters

PdxU2	Substrate-specific component PdxU2 of predicted pyridoxin-related ECF transporter
PdxU	Substrate-specific component PdxU of predicted pyridoxine ECF transporter

Transcriptional regulators for Pyridoxine metabolism

PdxR	Predicted transcriptional regulator of pyridoxine metabolism

Figure S1. (F) Biotin (vitamin B7) biosynthesis and salvage

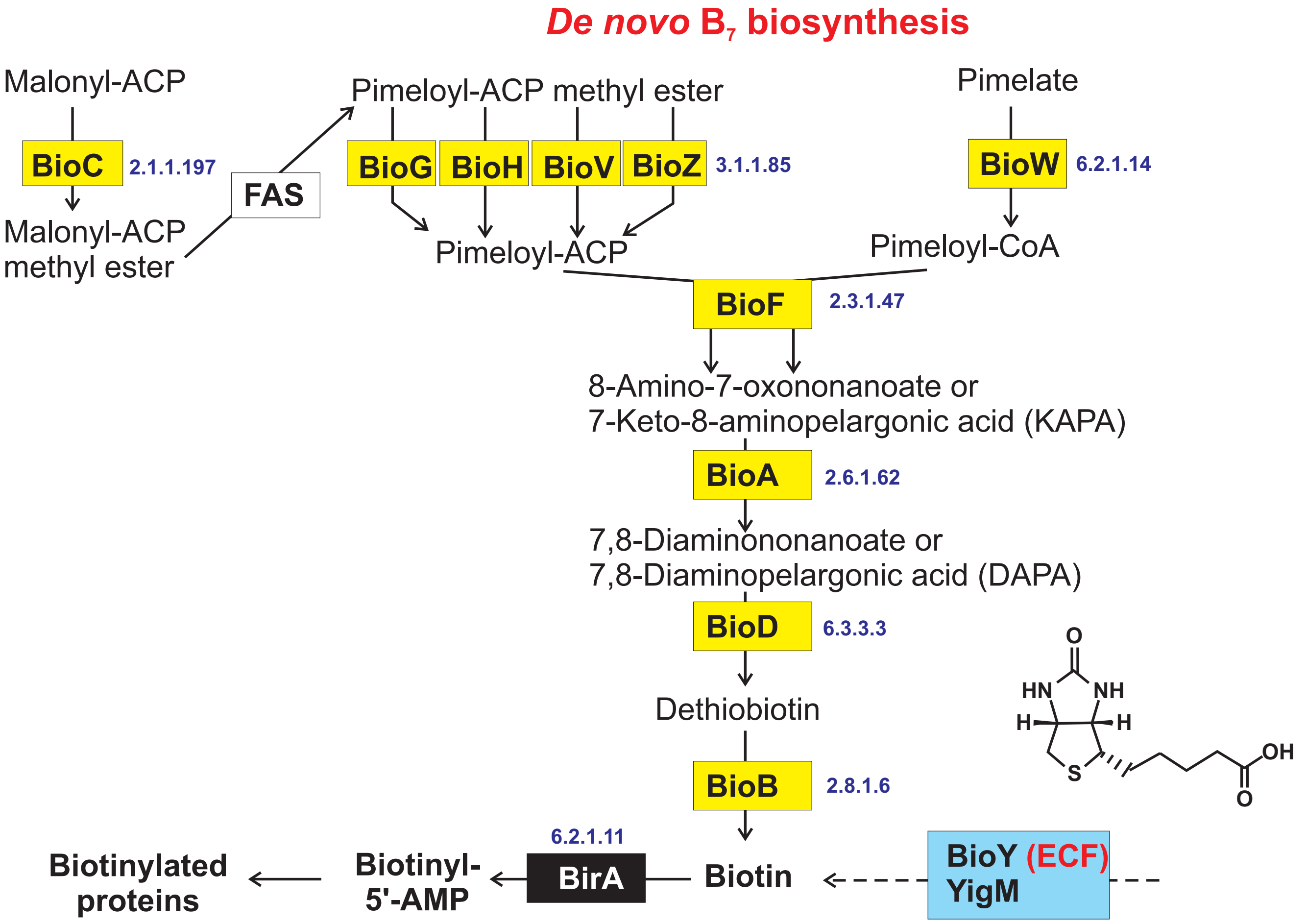

Biotin biosynthesis enzymes

BioF	8-amino-7-oxononanoate synthase (EC 2.3.1.47)
BioA	Adenosylmethionine-8-amino-7-oxononanoate aminotransferase (EC 2.6.1.62)
BioD	Dethiobiotin synthetase BioD (EC 6.3.3.3)
BioB	Biotin synthase (EC 2.8.1.6)
BioC	Malonyl-[acyl-carrier protein] O-methyltransferase (EC 2.1.1.197)
BioG	Pimeloyl-[acyl-carrier protein] methyl ester esterase BioG (EC 3.1.1.85)
BioH	Pimeloyl-[acyl-carrier protein] methyl ester esterase BioH (EC 3.1.1.85)
BioZ	Pimeloyl-[acyl-carrier protein] methyl ester esterase BioZ (EC 3.1.1.85)
BioV	Pimeloyl-[acyl-carrier protein] methyl ester esterase BioV (EC 3.1.1.85)
BioW	Pimeloyl-CoA synthase (EC 6.2.1.14)
BirA	Biotin--protein ligase (EC 6.3.4.9)(EC 6.3.4.10)(EC 6.3.4.11)(EC 6.3.4.15)

Biotin transporters

BioY	Substrate-specific component BioY of biotin ECF transporter
BioN	Transmembrane component BioN of energizing module of biotin ECF transporter
BioM	ATPase component BioM of energizing module of biotin ECF transporter
YigM	Biotin transporter YigM, drug/metabolite transporter (DMT) superfamily

Figure S1. (G) Folate (vitamin B9) biosynthesis and salvage

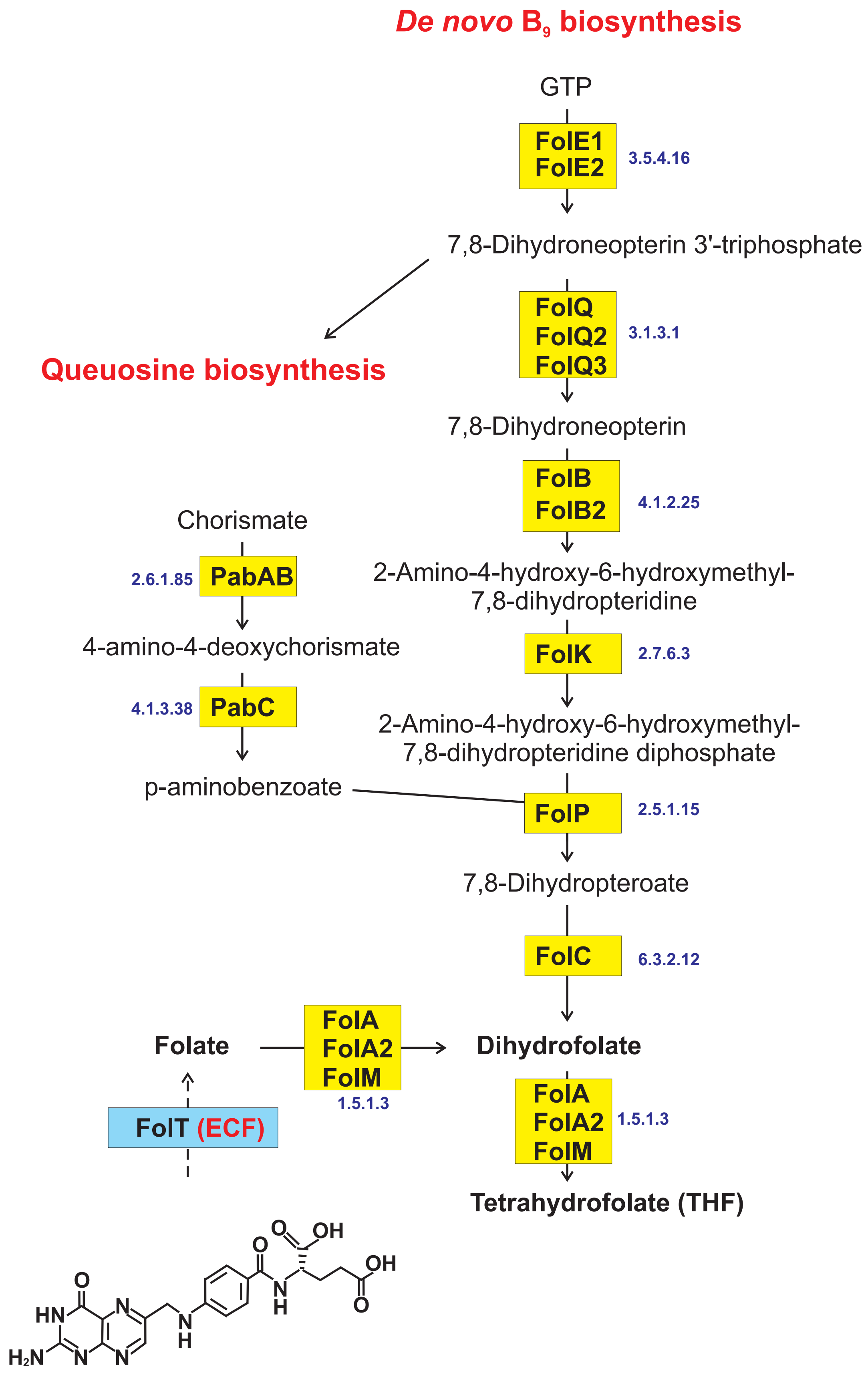

Figure S1. (G) Folate (vitamin B9) biosynthesis and salvage

Folate and Tetrahydrofolate biosynthesis enzymes

FolE1	GTP cyclohydrolase I (EC 3.5.4.16) type 1
FolE2	GTP cyclohydrolase I (EC 3.5.4.16) type 2
FolQ	Dihydroneopterin triphosphate pyrophosphohydolase
FolQ2	Dihydroneopterin triphosphate pyrophosphohydolase type 2
FolQ3	Putative DHNTP pyrophosphatase
FolB	Dihydroneopterin aldolase (EC 4.1.2.25)
FolB2	Folate biosynthesis protein PTPS-III, catalyzes a reaction that bypasses dihydroneopterin aldolase (FolB)
FolK	2-amino-4-hydroxy-6-hydroxymethyldihydropteridine pyrophosphokinase (EC 2.7.6.3)
FolP	Dihydropteroate synthase (EC 2.5.1.15)
FolA	Dihydrofolate reductase (EC 1.5.1.3)
FolM	Alternative dihydrofolate reductase 1
FolA2	Alternative dihydrofolate reductase 2
FolA3	Alternative dihydrofolate reductase 3
FolC	Dihydrofolate synthase (EC 6.3.2.12)
PabAa	Para-aminobenzoate synthase, aminase component (EC 2.6.1.85)
PabAb	Para-aminobenzoate synthase, amidotransferase component (EC 2.6.1.85)
PabAc	Aminodeoxychorismate lyase (EC 4.1.3.38)

Folate transporters

FolT	Substrate-specific component FolT of folate ECF transporter

Figure S1. (H) Cobalamin (vitamin B12) biosynthesis and salvage

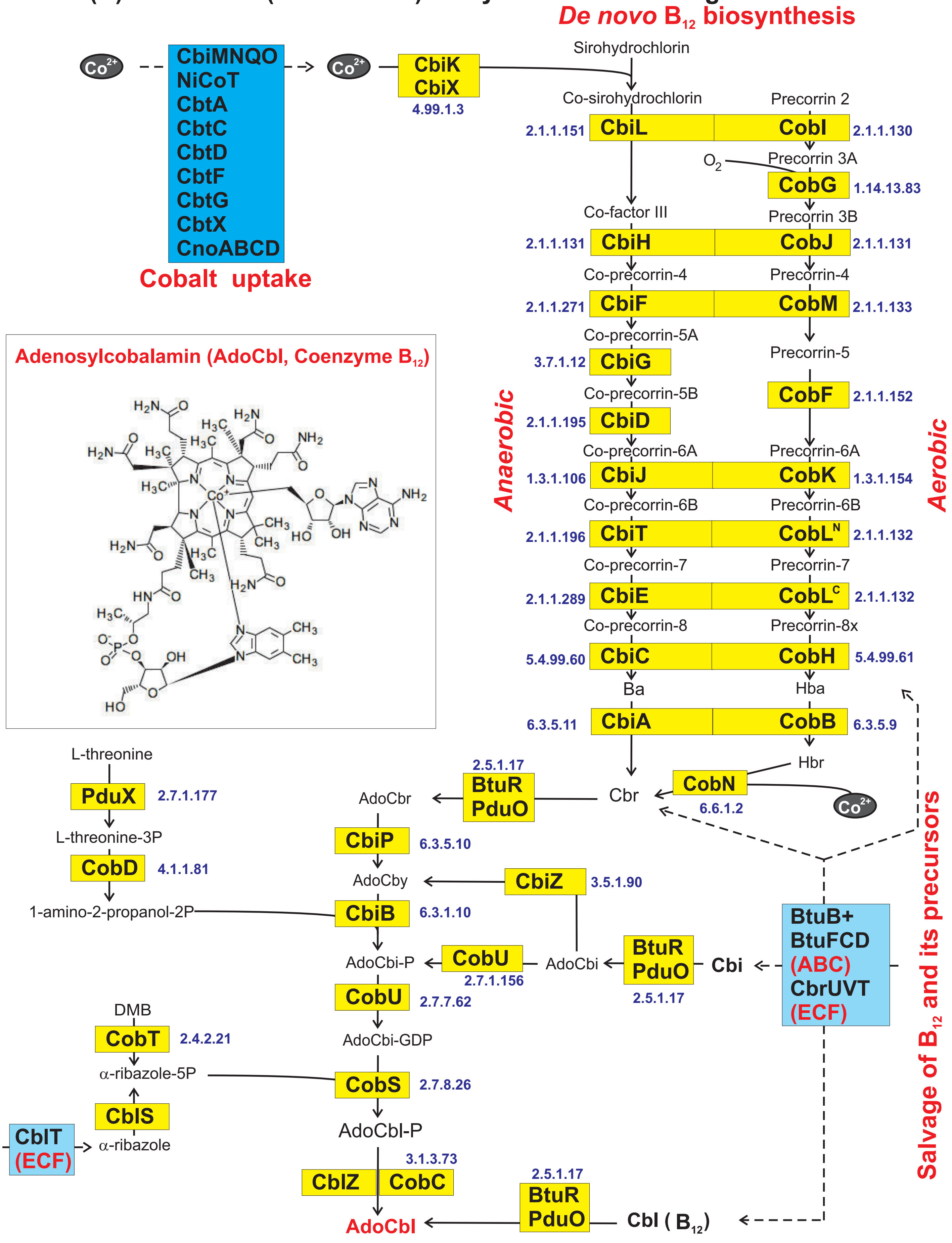

AdoCbl, Adenosylcobalamin; Cbl, Cobalamin; AdoCbi, Adenosylcobinamide; Cbi, Cobinamide; AdoCby, Adenosylcobyric acid; Cby, Cobyric acid; AdoCbr, Adenosylcobyrinate a,c-diamide; Cbr, Cobyrinate a,c-diamide; Ba, Cobyrinic acid; Hba, Hydrogenobyrinic acid; Hbr, Hydrogenobyrinic acid a,c-diamide; DMB, Dimethylbenzimidazole.

Figure S1. (H) Cobalamin (vitamin B12) biosynthesis and salvage

B12 biosynthesis / salvage enzymes

CbiX	Sirohydrochlorin cobaltochelatase (EC 4.99.1.3)
CbiX2	Sirohydrochlorin cobaltochelatase CbiX(long) (EC 4.99.1.3)
CbiK	Sirohydrochlorin cobaltochelatase CbiK (EC 4.99.1.3)
ChlI	ChlI component of cobalt chelatase involved in B12 biosynthesis
ChlD	ChlD component of cobalt chelatase involved in B12 biosynthesis
CobN	Aerobic cobaltochelatase CobN subunit (EC 6.6.1.2)
CbiL	Cobalt-precorrin-2 C(20)-methyltransferase (EC 2.1.1.151)
CbiH	Cobalt-precorrin-3 C(17)-methyltransferase (EC 2.1.1.272)
CbiF	Cobalt-precorrin-4 C(11)-methyltransferase (EC 2.1.1.271)
CbiD	Cobalt-precorrin-5B (C1)-methyltransferase (EC 2.1.1.195)
CbiT	Cobalt-precorrin-6B C15-methyltransferase [decarboxylating] (EC 2.1.1.196)
CbiE	Cobalt-precorrin-7 (C5)-methyltransferase (EC 2.1.1.289)
CbiG	Cobalt-precorrin 5A hydrolase (EC 3.7.1.12)
CbiJ	Cobalt-precorrin-6A reductase (EC 1.3.1.106)
CbiC	Cobalt-precorrin-8 methylmutase (EC 5.4.99.60)
CobG	Precorrin-3B synthase
CobF	Precorrin-6A synthase (deacetylating) (EC 2.1.1.152)
CbiA	Cobyrinic acid a,c-diamide synthetase (EC 6.3.5.11)
CbiP	Cobyric acid synthase (EC 6.3.5.10)
CbiB	Adenosylcobinamide-phosphate synthase (EC 6.3.1.10)
CobU	Adenosylcobinamide-phosphate guanylyltransferase (EC 2.7.7.62)
CobS	Cobalamin synthase (EC 2.7.8.26)
CobC	Alpha-ribazole-5'-phosphate phosphatase (EC 3.1.3.73)
PduX	L-threonine kinase in B12 biosynthesis (EC 2.7.1.177)
CobD	L-threonine 3-O-phosphate decarboxylase (EC 4.1.1.81)
CobT	Nicotinate-nucleotide--dimethylbenzimidazole phosphoribosyltransferase (EC 2.4.2.21)
BtuR	Cob(I)alamin adenosyltransferase (EC 2.5.1.17)
PduO	ATP:Cob(I)alamin adenosyltransferase (EC 2.5.1.17)
CblZ	CblZ, a non-orthologous displasment for Alpha-ribazole-5'-phosphate phosphatase

Transporters for B12 and precursors

BtuD	Vitamin B12 ABC transporter, ATPase component BtuD
BtuF	Vitamin B12 ABC transporter, B12 -binding component BtuF
BtuC	Vitamin B12 ABC transporter, permease component BtuC
BtuB	Outer membrane vitamin B12 receptor BtuB
CbrU	Duplicated ATPase component CbrU of energizing module of predicted cobalamin ECF transporter
CbrV	Transmembrane component CbrV of energizing module o f predicted cobalamin ECF transporter
CbrT	Substrate-specific component CbrT of predicted cobalamin ECF transporter

Figure S1. (H) Cobalamin (vitamin B12) biosynthesis and salvage

Transporters for Cobalt (required for B12 biosynthesis)

CbiQ	Transmembrane component CbiQ of energizing module of cobalt ECF transporter
CbiO	ATPase component CbiO of energizing module of cobalt ECF transporter
CbiN	Additional substrate-specific component CbiN of cobalt ECF transporter
NiCOT	HoxN/HupN/NixA family cobalt transporter
HupE	HupE-UreJ family cobalt transporter
CbtX	Predicted cobalt transporter in sulfate-reducing delta-proteobacteria
CbtA	Predicted cobalt transporter CbtA
CbtC	Predicted cobalt transporter CbtC
CbtD	Predicted cobalt transporter in Bacteroides_Porphyromonas
CbtF	Predicted cobalt ABC transporter periplasmic component
CbtX	Predicted cobalt transporter in sulfate-reducing delta-proteobacteria
CnoA	Predicted cobalt ABC transporter, periplasmic substrate -binding component CnoA
CnoB	Predicted cobalt ABC transporter, permease protein CnoB
CnoC	Predicted cobalt ABC transporter, permease protein CnoC
CnoD	Predicted cobalt ABC transporter, ATP -binding protein CnoD

Salvage of dimethylbenzimidazole precursor

CblT	Substrate-specific component CblT of predicted B12-regulated ECF transporter for dimethylbenzimidazole
CblS	Predicted alpha-ribazole-5-phosphate synthase CblS for cobalamin biosynthesis

Figure S1. (I) Queuosine biosynthesis and salvage

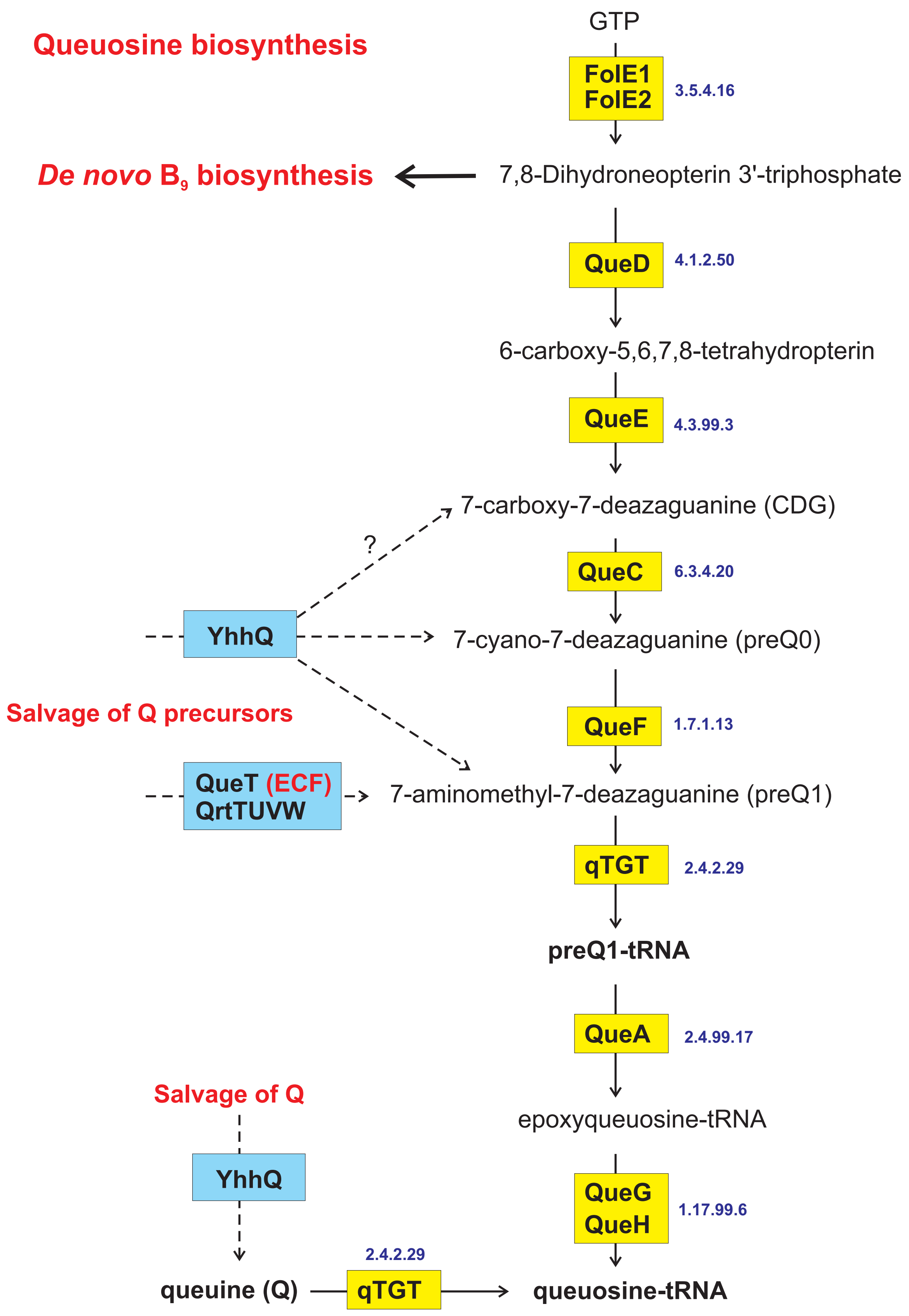

Figure S1. (I) Queuosine biosynthesis and salvage

Q biosynthesis / salvage enzymes

GCYHI1	GTP cyclohydrolase I (EC 3.5.4.16) type 1
GCYHI2	GTP cyclohydrolase I (EC 3.5.4.16) type 2
QueD	6-carboxy-5,6,7,8-tetrahydropterin synthase (EC 4.1.2.50)
QueE	7-carboxy-7-deazaguanine synthase (EC 4.3.99.3)
QueC	7-cyano-7-deazaguanine synthase (EC 6.3.4.20)
QueF	NADPH-dependent 7-cyano-7-deazaguanine reductase (EC 1.7.1.13)
qTGT	Queuine tRNA-ribosyltransferase (EC 2.4.2.29)
QueA	S-adenosylmethionine:tRNA ribosyltransferase-isomerase (EC 2.4.99.17)
QueG	Epoxyqueuosine reductase (EC 1.17.99.6) QueG
QueH	Epoxyqueuosine reductase (EC 1.17.99.6) QueH

Transporters for Q precursors

QrtT	Substrate-specific component STY3230 of queuosine-regulated ECF transporter
QrtU	Transmembrane component STY3231 of energizing module of queuosine-regulated ECF transporter
QrtV	ATPase component STY3232 of energizing module of queuosine-regulated ECF transporter
QrtW	ATPase component STY3233 of energizing module of queuosine-regulated ECF transporter
QueT	Substrate-specific component QueT (COG4708) of predicted queuosine-regulated ECF transporter
YhhQ	Putative preQ0 transporter YhhQ
