# Supplementary material for: Micronutrient Requirements and Sharing Capabilities of the Human Gut Microbiome
Source: Front Microbiol. 2019 Jun 12;10:1316. doi: 10.3389/fmicb.2019.01316 (PMC6593275; doi:10.3389/fmicb.2019.01316)
Supplement: Supplementary file 2 [file Image_2.pdf]

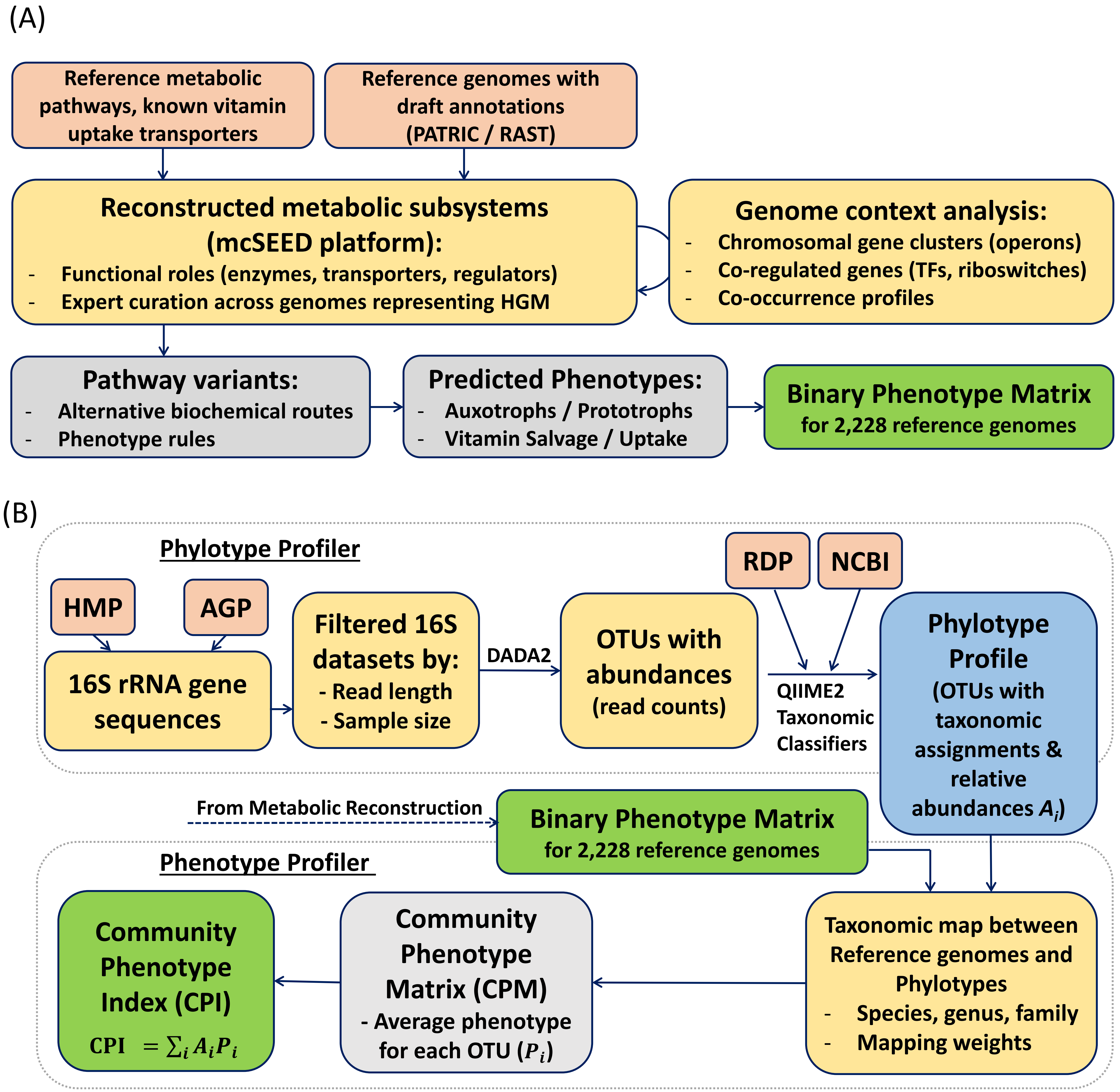

Figure S2. Workflows used for (A) comparative genomic reconstruction of metabolic pathways and phenotype assignments in reference genomes, and (B) predictive phenotype profiling of microbial communities derived from 16S samples.
