# Supplementary material for: Micronutrient Requirements and Sharing Capabilities of the Human Gut Microbiome
Source: Front Microbiol. 2019 Jun 12;10:1316. doi: 10.3389/fmicb.2019.01316 (PMC6593275; doi:10.3389/fmicb.2019.01316)
Supplement: Supplementary file 3 [file Image_3.pdf]

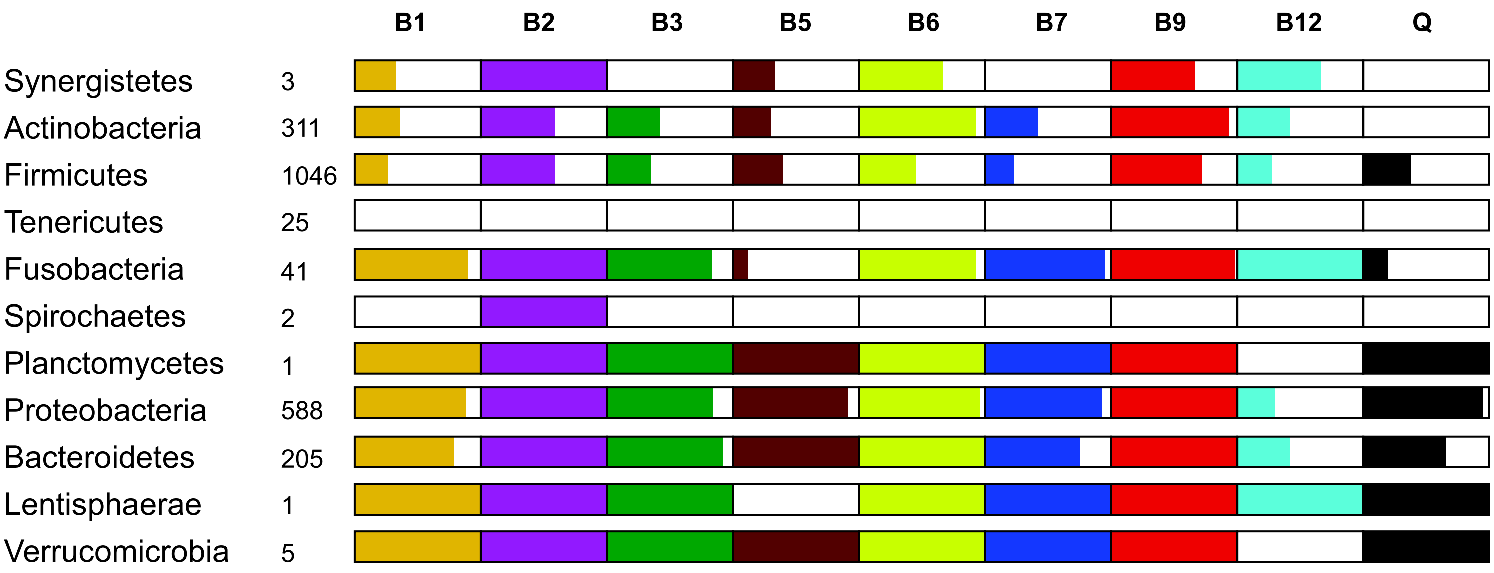

Figure S3. Distribution of vitamin producers among major phyla of analyzed HGM strains.
Colored bars show the percentage of vitamin producers in each phylum.
The number of analyzed strains is given next to each phylum name.
