# Supplementary material for: Micronutrient Requirements and Sharing Capabilities of the Human Gut Microbiome
Source: Front Microbiol. 2019 Jun 12;10:1316. doi: 10.3389/fmicb.2019.01316 (PMC6593275; doi:10.3389/fmicb.2019.01316)
Supplement: Supplementary file 4 [file Image_4.pdf]

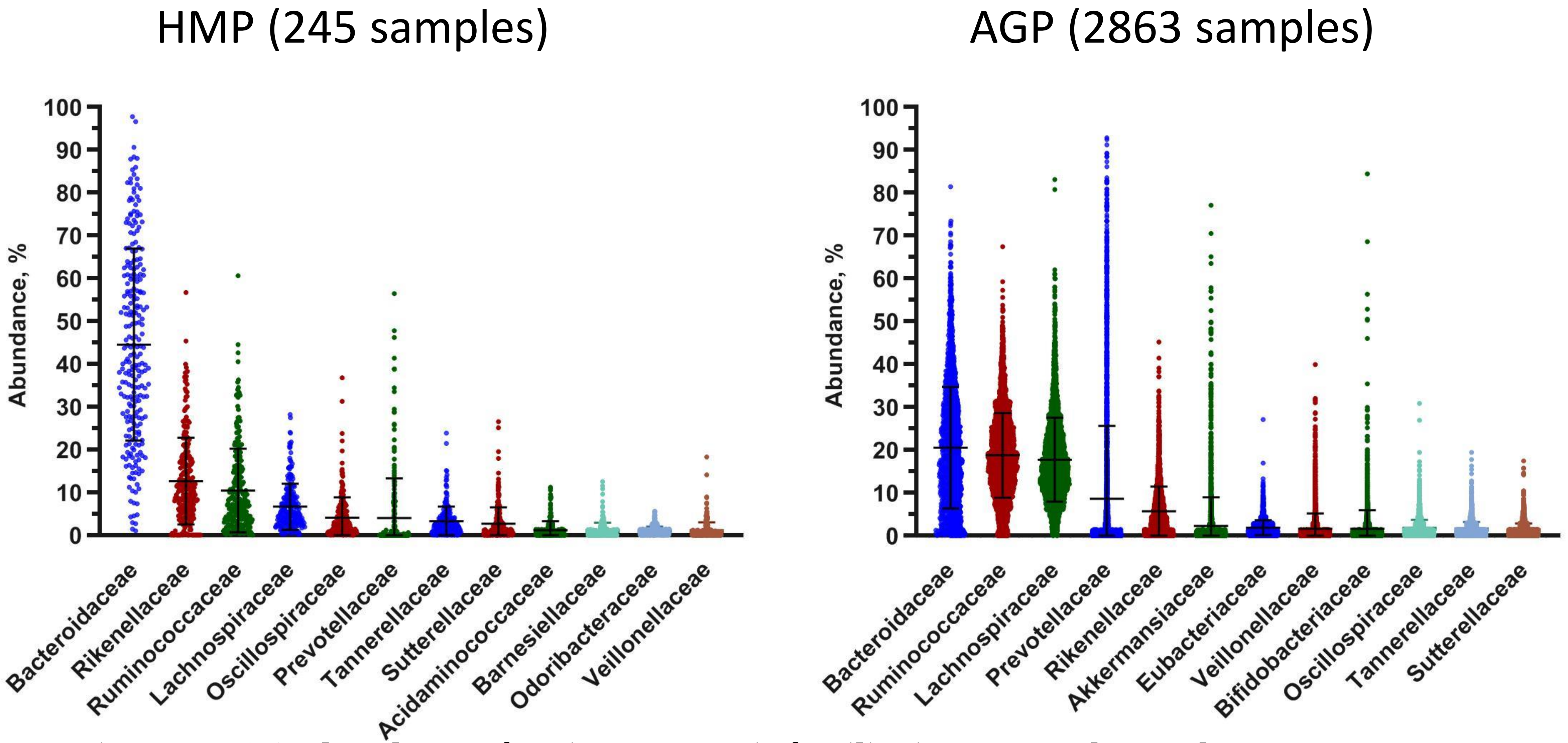

Figure S4. (A) Abundance of major taxonomic families in HMP and AGP datasets.
The obtained taxonomic profiles for HMP and AGP datasets differ substantially in abundance of top contributing families. The HMP dataset is characterized by elevated average abundance of two families, Bacteroidaceae (*Bacteroides* spp.) and Rikenellaceae (*Alistipes* spp.). In contrast, three major contributing families in the AGP dataset are Bacteroidaceae, Lachnospiraceae (*Blautia*, *Roseburia*, *Lachnoclostridium* spp.) and Ruminococcaceae (*Faecalibacterium*, *Ruminococcus* spp.).

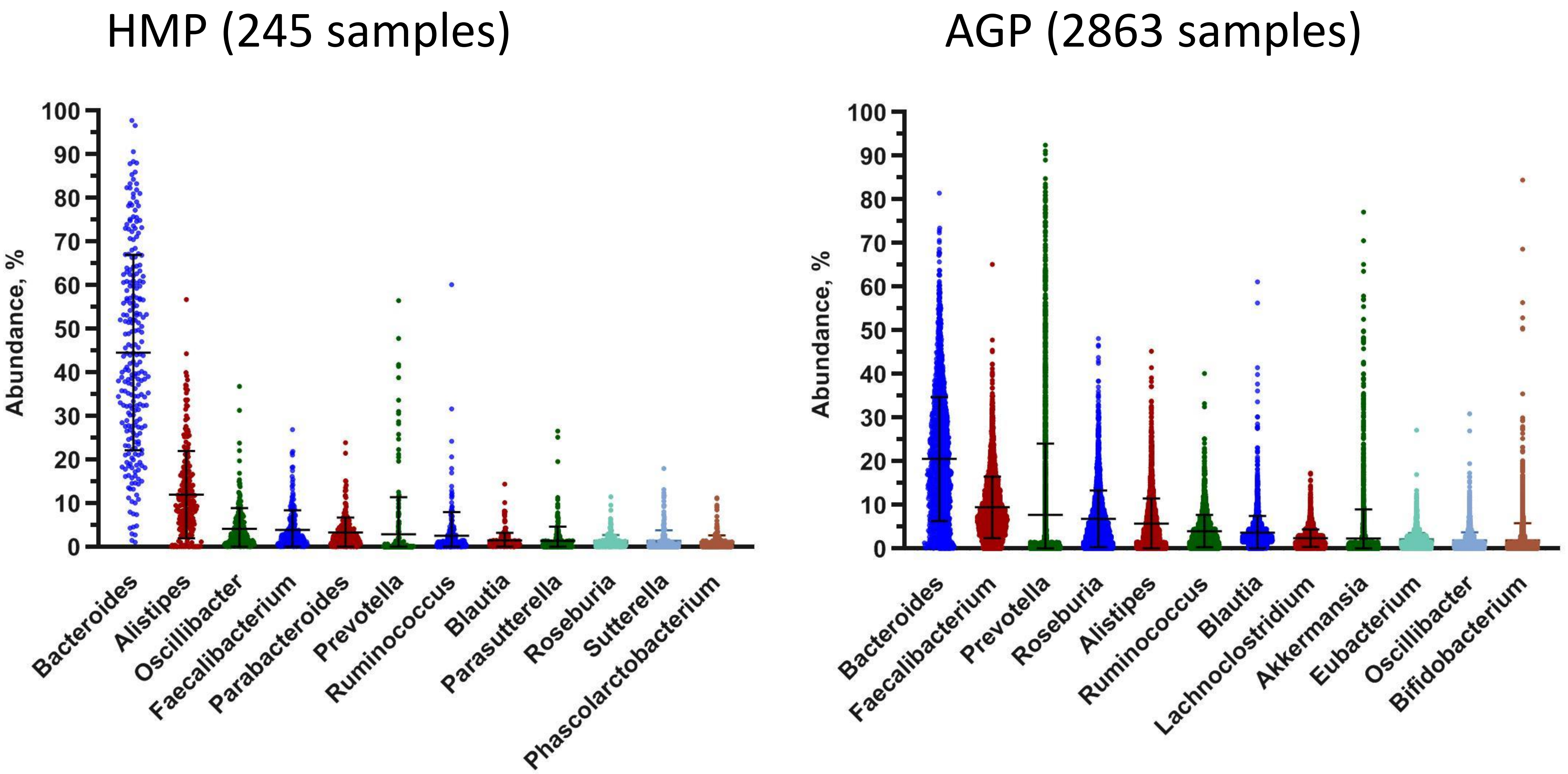

Figure S4. (B) Abundance of major taxonomic genera in HMP and AGP datasets.
The obtained taxonomic profiles for HMP and AGP datasets differ substantially in abundance of top contributing genera.
The HMP dataset is characterized by elevated average abundance of two genera, *Bacteroides* and *Alistipes*.
In contrast, three major contributing genera in the AGP dataset are *Bacteroides*, *Prevotella*, *Blautia*, *Roseburia*, *Lachnoclostridium*, *Faecalibacterium*, and *Ruminococcus*.

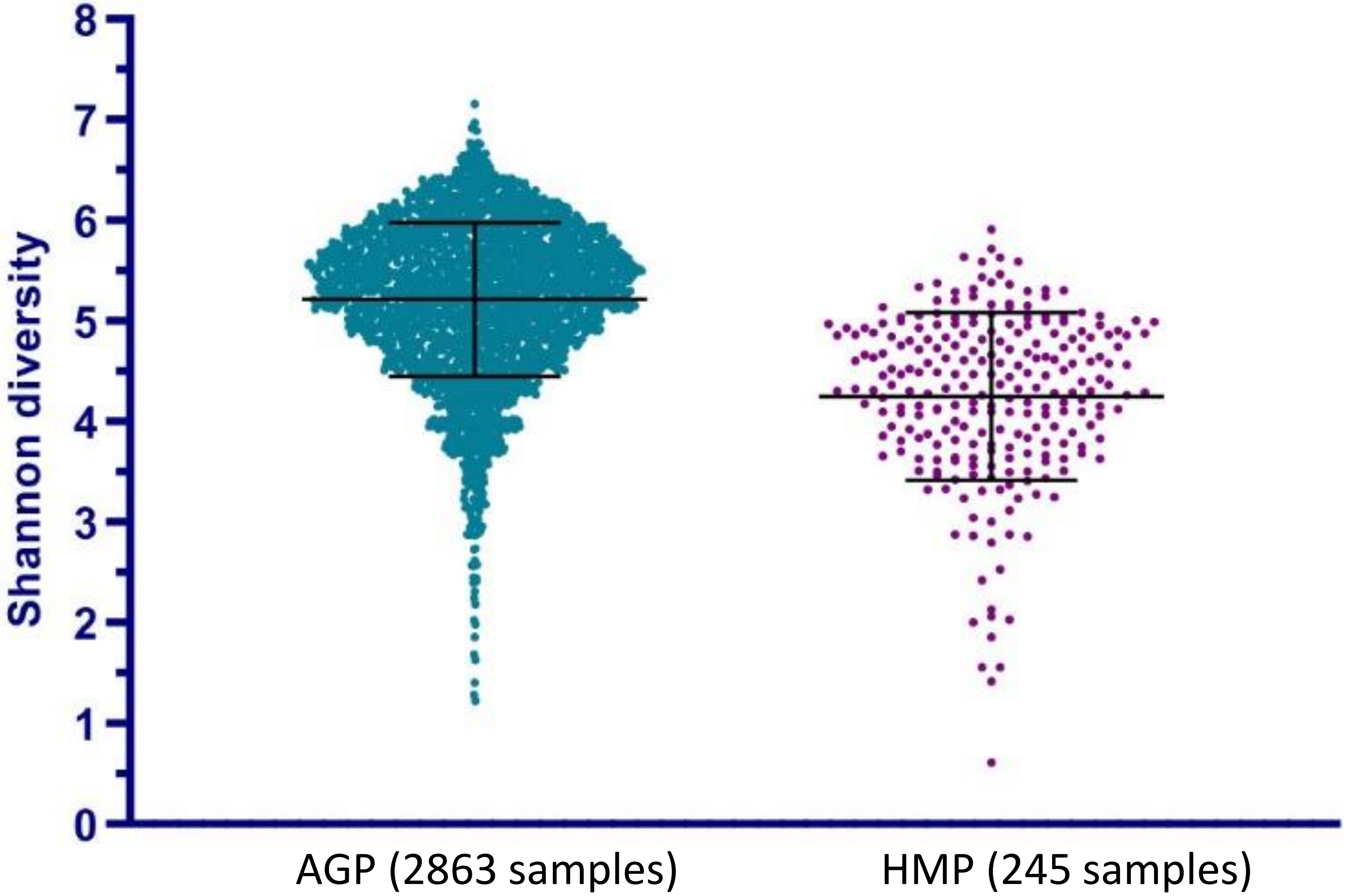

Figure S4. (C) Distribution of the Shannon diversity index in HMP and AGP datasets.
