# Supplementary material for: Micronutrient Requirements and Sharing Capabilities of the Human Gut Microbiome
Source: Front Microbiol. 2019 Jun 12;10:1316. doi: 10.3389/fmicb.2019.01316 (PMC6593275; doi:10.3389/fmicb.2019.01316)
Supplement: Supplementary file 5 [file Image_5.pdf]

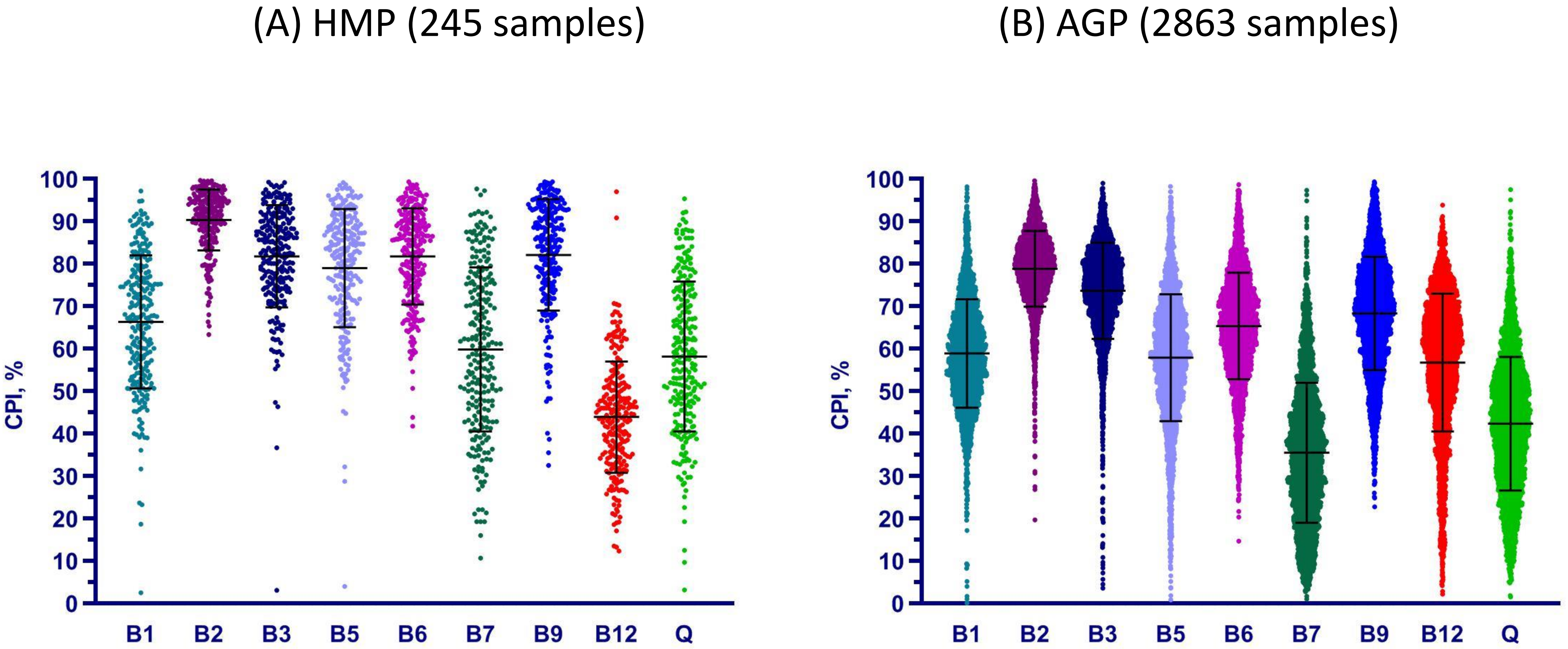

Figure S5. Distribution of Community Phenotype Indices for B and Q vitamins in (A) HMP and (B) AGP datasets after renormalization by 16S gene count numbers.
