# Supplementary material for: Micronutrient Requirements and Sharing Capabilities of the Human Gut Microbiome
Source: Front Microbiol. 2019 Jun 12;10:1316. doi: 10.3389/fmicb.2019.01316 (PMC6593275; doi:10.3389/fmicb.2019.01316)
Supplement: Supplementary file 6 [file Image_6.pdf]

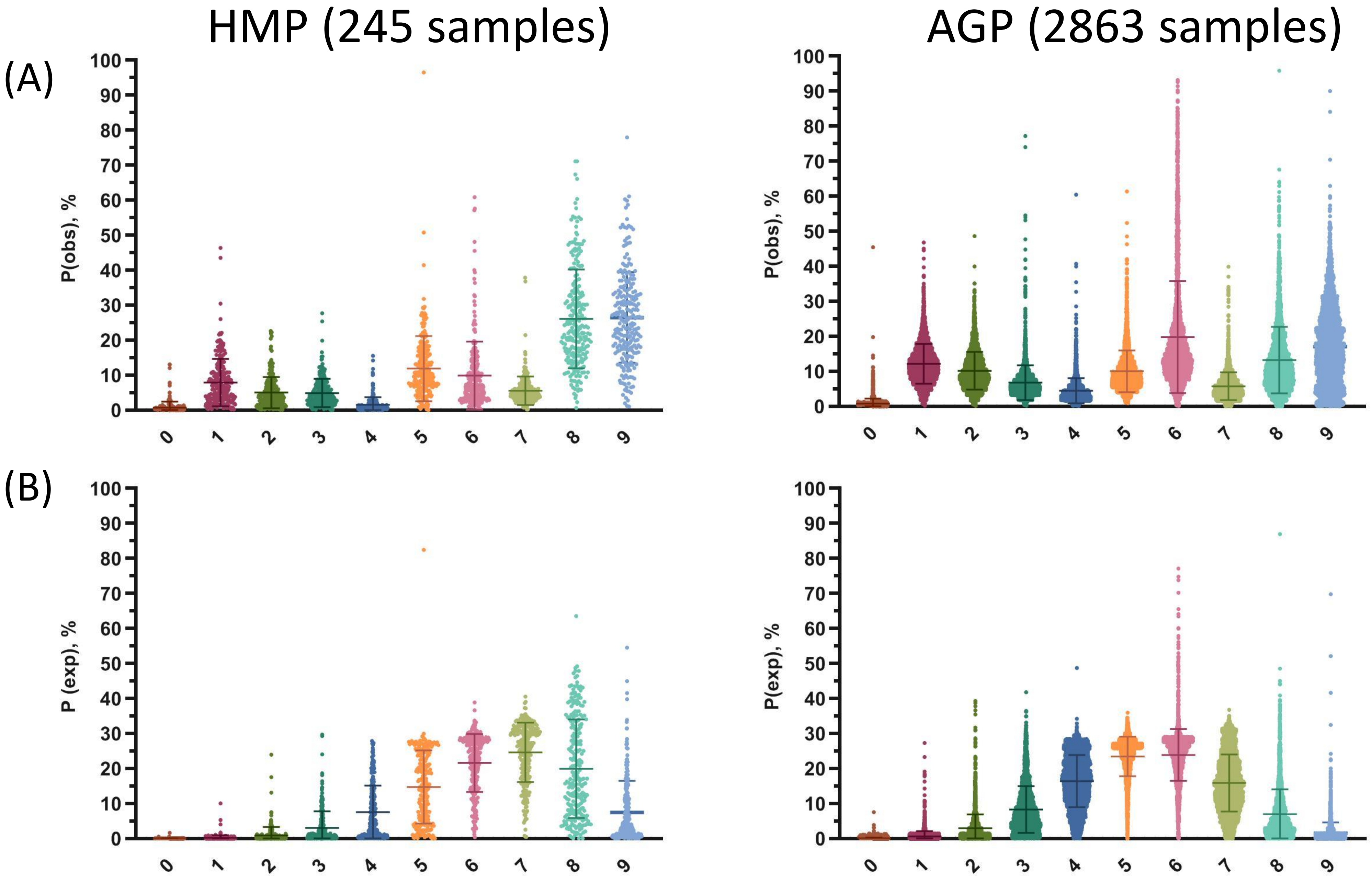

Figure S6. Observed (A) and expected (B) probabilities for Vitamin Prototrophy Ranks (VPR) in HMP and AGP datasets.
