# Supplementary material for: Micronutrient Requirements and Sharing Capabilities of the Human Gut Microbiome
Source: Front Microbiol. 2019 Jun 12;10:1316. doi: 10.3389/fmicb.2019.01316 (PMC6593275; doi:10.3389/fmicb.2019.01316)
Supplement: Supplementary file 7 [file Image_7.pdf]

Supplementary Figure S7. Comparison of vitamin production phenotype abundances (calculated by PICRUSt2 and based on binary phenotypes from BPM) with MetaCyc pathway abundances (obtained by the standard PICRUSt2/MinPath approach) for HMP and AGP datasets.

Linear regression line is shown in blue. The main diagonal (intersect=0 and slope=1) is shown as a dashed line indicating equality in predictions. Correlation coefficients "C" and coefficients of determination "R^2" are shown.

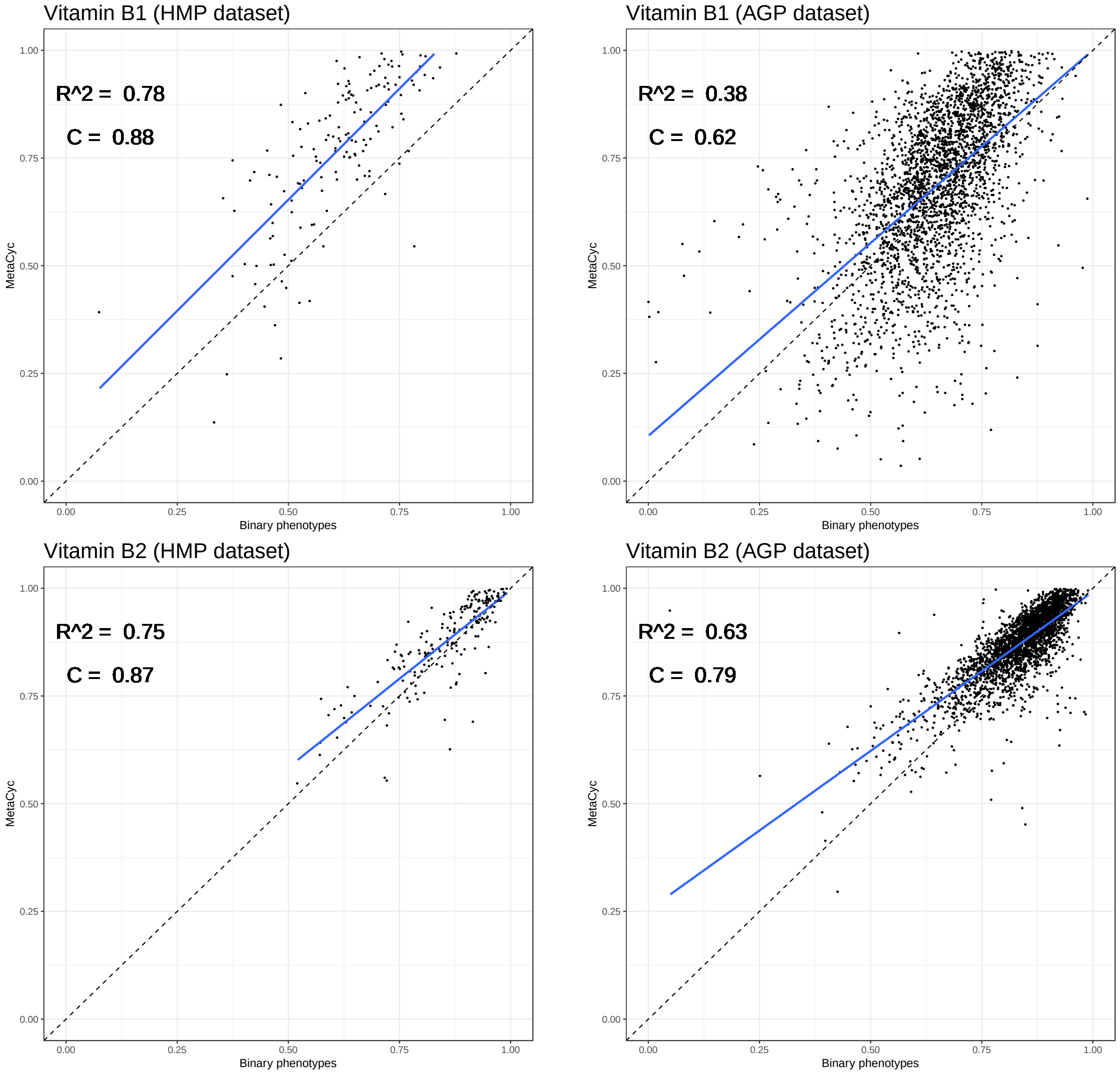

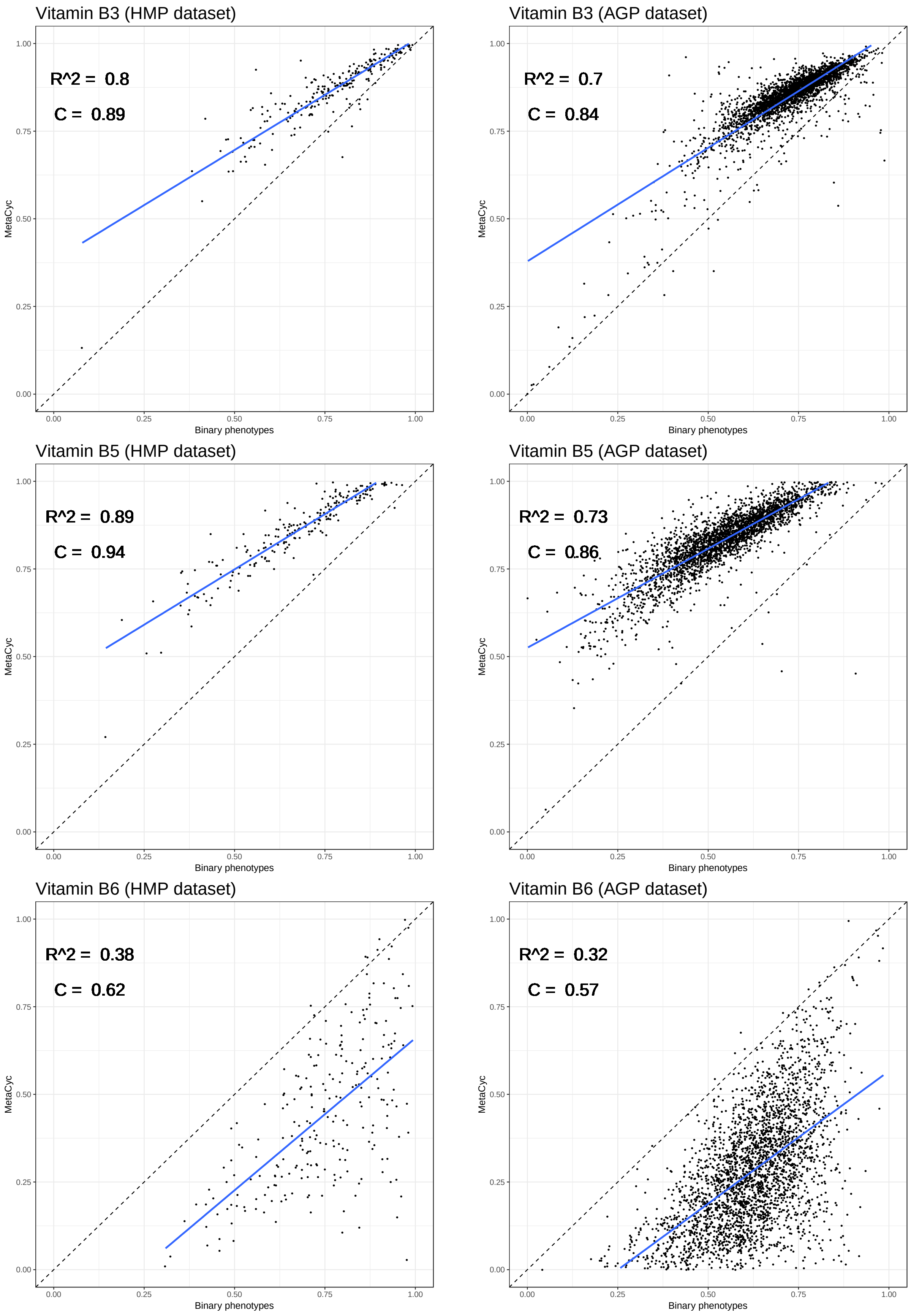
Vitamin B3 (HMP dataset)
R^2 = 0.8
C = 0.89
Vitamin B3 (AGP dataset)
R^2 = 0.7
C = 0.84
Vitamin B5 (HMP dataset)
R^2 = 0.89
C = 0.94
Vitamin B5 (AGP dataset)
R^2 = 0.73
C = 0.86
Vitamin B6 (HMP dataset)
R^2 = 0.38
C = 0.62
Vitamin B6 (AGP dataset)
R^2 = 0.32
C = 0.57
MetaCyc
Binary phenotypes
0.00
0.25
0.50
0.75
1.00

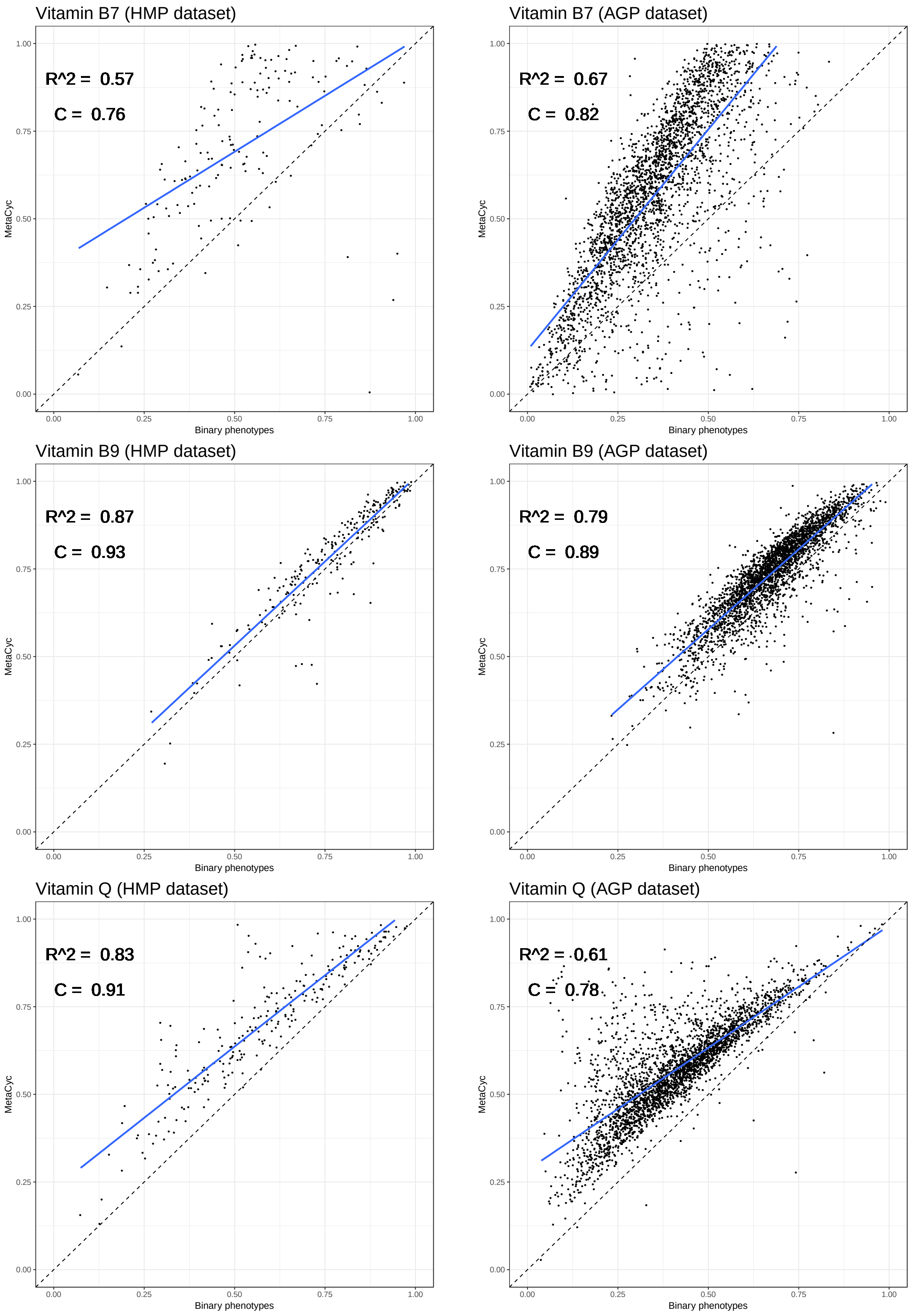
Vitamin B7 (HMP dataset)
R^2 = 0.57
C = 0.76
Vitamin B7 (AGP dataset)
R^2 = 0.67
C = 0.82
Vitamin B9 (HMP dataset)
R^2 = 0.87
C = 0.93
Vitamin B9 (AGP dataset)
R^2 = 0.79
C = 0.89
Vitamin Q (HMP dataset)
R^2 = 0.83
C = 0.91
Vitamin Q (AGP dataset)
R^2 = 0.61
C = 0.78
MetaCyc
Binary phenotypes
0.00
0.25
0.50
0.75
1.00
