# Supplementary material for: Micronutrient Requirements and Sharing Capabilities of the Human Gut Microbiome
Source: Front Microbiol. 2019 Jun 12;10:1316. doi: 10.3389/fmicb.2019.01316 (PMC6593275; doi:10.3389/fmicb.2019.01316)
Supplement: Supplementary file 10 [file Table_3.pdf]

Table S3. A. Experimentally described B-vitamin growth requirements (except B12) of human gut bacteria.

Experimentally studied strains/species [1]	B-vitamin requirements [2]	References
Lactobacillus delbrueckii	B2, B3, B5, B6	(Hébert et al., 2004)
Lactobacillus plantarum WCFS1	B2, B3, B5, B9[3]	(Wegkamp et al., 2010)
Campylobacter jejuni NCTC 11168	B3	(Alazzam et al., 2011)
Helicobacter pylori 26695	B1	(Testerman et al., 2006)
Neisseria cinerea ATCC 14685	B7	(McDonald and Johnson, 1975)
Propionibacterium freudenreichii subsp. *shermanii* CIRM-BIA1	B1, B5, B7	(Falentin et al., 2010)
Propionibacterium freudenreichii subsp. *freudenreichii*	B5, B7	(Delwiche, 1949)
Propionibacterium jensenii	B5, B7	(Delwiche, 1949)
Listeria monocytogenes serotype 1/2a	B1, B2, B7	(Tsai and Hodgson, 2003)
Staphylococcus aureus	B1, B3	(Cove et al., 1980)
Staphylococcus capitis	B1, B3, B7	(Cove et al., 1980)
Staphylococcus cohnii	B1, B3, B5, B7	(Cove et al., 1983)
Staphylococcus epidermidis	B1, B3, B7	(Cove et al., 1980)
Staphylococcus haemolyticus	B1, B3, B7	(Cove et al., 1980)
Staphylococcus hominis	B1, B3, B7	(Cove et al., 1980)
Staphylococcus saprophyticus	B1, B3, B7	(Cove et al., 1980)
Staphylococcus simulans	B1, B3, B7	(Cove et al., 1980)
Staphylococcus warneri	B1, B3, B7	(Cove et al., 1980)
Streptococcus thermophilus	B1, B2, B3, B5, B6	(Letort and Juillard, 2001)
Clostridium botulinum A str. Hall	B1, B7, B9[3]	(Whitmer and Johnson, 1988)
Clostridium botulinum E3 str. Alaska	B1, B7, B9	(Whitmer and Johnson, 1988)
Clostridium butyricum	B7	(Himmi et al., 1999)
Clostridium difficile	B5[4], B6, B7	(Karasawa et al., 1995)
Clostridium perfringens	B3, B5, B6, B7	(Fuchs and Bonde, 1957; Goldner et al., 1985)
Clostridium sporogenes	B7, B9[3]	(Grossowicz et al., 1956)
Clostridium tyrobutyricum	B7, B9[3]	(Storari et al., 2016)
Faecalibacterium prausnitzii A2-165	B1, B2, B3, B5, B6, B7, B9	(Khan et al., 2012; Heinken et al., 2014)
Ruminococcus bromii	B2, B5[5], B6, B7, B9	(Herbeck and Bryant, 1974)
Megasphaera elsdenii	B5[4], B6, B7	(Forsberg, 1978)
Veillonella parvula	B1, B6, B7, B9[3]	(Rogosa and Bishop, 1964)

1Strain name is indicated only if (a) it was included in the original publication, and (b) it is present in the reference collection of genomes analyzed in this work; in all other cases only a species name is included.

2 Vitamin requirements that are consistent with the predicted in this work auxotrophic (A) phenotypes are shown in black, while the inconsistent phenotypes are in red.

B9[3] indicates that organism requires pABA, a precursor of B9, which is consistent with the absence of pABA synthesis pathway, while all other enzymes of B9 biosynthesis pathway are present (B9 prototroph with missing pABA pathway).

B5[4] indicates that organism may require β-alanine, a precursor of B5, since the β-alanine synthesis pathway is missing, while all other enzymes of B5 biosynthesis pathway are present (B5 prototroph with missing β-alanine pathway).

B5[5] indicates that organism is predicted to require pantetheine, a precursor of Coenzyme A which is synthesized from pantothenate, since only the pantetheine salvage pathway was identified in its genome.

Table S3. B. Experimentally described growth requirements for vitamin B12 in human gut bacteria.

Experimentally studied strains / species [1]	B12 requirements [2]	References
Bacteroides caccae ATCC 43185	RG	(Goodman et al., 2009)
Bacteroides fragilis NCTC 9343	RG	(Varel and Bryant, 1974)
Bacteroides ovatus ATCC 8483	RG	(Goodman et al., 2009)
Bacteroides vulgatus ATCC 8482	RG	(Varel and Bryant, 1974)
Bacteroides thetaiotaomicron VPI-5482	RG	(Degnan et al., 2014)
Parabacteroides distasonis ATCC 8503	RG	(Goodman et al., 2009; Varel and Bryant, 1974)
Porphyromonas gingivalis W83	RG	(Roper et al., 2000)
Lactobacillus delbrueckii	RG	(Rao et al., 1984; Hébert et al., 2004)
Lactobacillus sakei	RG	(Masuda et al., 2012)
Lactobacillus plantarum WCFS1	NEFG	(Wegkamp et al., 2010)
Akkermansia muciniphila ATCC BAA-835	NEFG	(Derrien et al., 2004)
Escherichia coli K-12	RG	(Giannella et al., 1971; Lawrence and Roth, 1996)
Escherichia fergusonii ATCC 35469	RG	(Lawrence and Roth, 1996)
Shigella dysenteriae	RG	(Lawrence and Roth, 1996)
Shigella flexneri	RG	(Giannella et al., 1971; Lawrence and Roth, 1996)
Shigella sonnei A	RG	(Giannella et al., 1971; Lawrence and Roth, 1996)
Serratia fonticola	RG	Lawrence and Roth, 1996
Serratia marcescens	RG	Lawrence and Roth, 1996
Campylobacter jejuni subsp. *jejuni* NCTC 11168	NEFG	(Alazzam et al., 2011)
Helicobacter pylori 26695	NEFG	(Testerman et al., 2006)
Neisseria cinerea ATCC 14685	NEFG	(McDonald and Johnson, 1975)
Neisseria flavescens ATCC 13120	NEFG	(McDonald and Johnson, 1975)
Collinsella aerofaciens ATCC 25986	RG	(Goodman et al., 2009)
Bacillus cereus ATCC 14579	NEFG	(Abbas et al., 2014)
Staphylococcus aureus, S. capitis, S. cohnii, S. epidermidis, S. haemolyticus, S. hominis, S. saprophyticus, S. simulans, S. warneri	NEFG	(Cove et al., 1980; Cove et al., 1983)
Streptococcus thermophilus	NEFG	(Letort and Juillard, 2001)
Clostridium scindens ATCC 35704	RG	(Goodman et al., 2009)
Clostridium spiroforme DSM 1552	RG	(Goodman et al., 2009)
Ruminococcus bromii	RG	(Herbeck and Bryant, 1974)

[1] Strain name is indicated only if (a) it was included in the original publication, and (b) it is present in the reference collection of genomes analyzed in this work; in all other cases only a species name is included.

[2] Experimental phenotypes: RG, requires B12 for growth; NEFG, B12 is not essential for growth. The RG phenotypes that are consistent with the predicted B12 auxotrophic phenotypes are shown in black, while the inconsistent phenotypes are in red.

Table S3. C. Experimentally described B-vitamin production capabilities of human gut bacteria.

Experimentally studied strains / species [1]	B-vitamin production[2]	References
Bacteroides vulgatus	B1, B2, B3, B7	(Burkholder and McVeigh, 1942)
Bacteroides thetaiotaomicron VPI-5482	B1	(Costliow and Degnan, 2017)
Lactobacillus acidopbilus	B9[3]	(Rao et al., 1984)
Lactobacillus coryniformis	B9[3], B12	(Masuda et al., 2012)
Lactobacillus fermentum	B9[3]	(Hugenschmidt et al., 2010)
Lactobacillus plantarum	B9[3]	(Hugenschmidt et al., 2010)
Lactobacillus sakei	B9[3]	(Masuda et al., 2012)
Lactobacillus reuteri JCM 1112	B9[3], B12	(Santos et al., 2008)
Escherichia coli	B1, B2, B3, B7	(Burkholder and McVeigh, 1942)
Proteus vulgaris	B1, B2, B3, B7	(Burkholder and McVeigh, 1942)
Salmonella enterica subsp. *enterica* serovar Typhimurium str. Lt2	B12	(Giannella et al., 1971; Lawrence and Roth, 1996)
Citrobacter freundii	B12	(Keuth and Bisping, 1994; Lawrence and Roth, 1996)
Klebsiella pneumoniae	B12	(Giannella et al., 1971; Keuth and Bisping, 1994; Lawrence and Roth, 1996)
Bifidobacterium adolescentis ATCC 15703&DSM 20087	B6, B9	(Deguchi et al., 1985; SUGAHARA et al., 2015; Kopp et al., 2017)
Bifidobacterium bifidum ATCC 29521	B1, B3, B6, B9	(Deguchi et al., 1985; SUGAHARA et al., 2015)
Bifidobacterium breve MCC 0121&1114&1604	B1, B3, B6, B9[3]	(Deguchi et al., 1985; SUGAHARA et al., 2015)
Bifidobacterium longum subsp. *infantis* ATCC 15697	B1, B3, B6, B9	(Deguchi et al., 1985; Pompei et al., 2007; SUGAHARA et al., 2015)
Bifidobacterium longum subsp. *longum*	B1, B3, B6, B9	(Deguchi et al., 1985)
Bifidobacterium pseudocatenulatum DSM 20438	B9[3]	(Kopp et al., 2017)
Propionibacterium freudenreichii	B12	(Hugenschmidt et al., 2010)
Bacillus clausii	B2	(Salvetti et al., 2003)
Bacillus megaterium DSM319	B12	(Moore et al., 2014)
Bacillus pumilus	B1, B2, B3	(Burkholder and McVeigh, 1942)
Ruminococcus obeum ATCC 29174	B12	(Goodman et al., 2009)

[1] Strain name is indicated only if (a) it was included in the original publication, and (b) it is present in the reference collection of genomes analyzed in this work; in all other cases only a species name is included.

[2]All experimental vitamin production phenotypes are consistent with the predicted in this work prototrophic (P) phenotypes. B9[3] indicates that organism requires pABA, a precursor of B9, which is consistent with the absence of pABA synthesis pathway, while all other enzymes of B9 biosynthesis pathway are present (B9 prototroph with missing pABA pathway).

References

Abbas, A. A., Planchon, S., Jobin, M., and Schmitt, P. (2014). A new chemically defined medium for the growth and sporulation of Bacillus cereus strains in anaerobiosis. *J. Microbiol. Methods* 105, 54–58. doi:10.1016/j.mimet.2014.07.006.

Alazzam, B., Bonnassie-Rouxin, S., Dufour, V., and Ermel, G. (2011). MCLMAN, a new minimal medium for Campylobacter jejuni NCTC 11168. *Res. Microbiol.* 162, 173–179. doi:10.1016/j.resmic.2010.09.024.

Burkholder, P. R., and McVeigh, I. (1942). Synthesis of Vitamins by Intestinal Bacteria. *Proc. Natl. Acad. Sci. U. S. A.* 28, 285–289.

Costliow, Z. A., and Degnan, P. H. (2017). Thiamine Acquisition Strategies Impact Metabolism and Competition in the Gut Microbe Bacteroides thetaiotaomicron. *mSystems* 2. doi:10.1128/mSystems.00116-17.

Cove, J. H., Holland, K. T., and Cunliffe, W. J. (1980). The vitamin requirements of staphylococci isolated from human skin. *J. Appl. Bacteriol.* 49, 29–37.

Cove, J. H., Kearney, J. N., Holland, K. T., and Cunliffe, W. J. (1983). The vitamin requirements of Staphylococcus cohnii. *J. Appl. Bacteriol.* 54, 203–208.

Degnan, P. H., Barry, N. A., Mok, K. C., Taga, M. E., and Goodman, A. L. (2014). Human gut microbes use multiple transporters to distinguish vitamin B12 analogs and compete in the gut. *Cell Host Microbe* 15, 47–57. doi:10.1016/j.chom.2013.12.007.

Deguchi, Y., Morishita, T., and Mutai, M. (1985). Comparative Studies on Synthesis of Water-soluble Vitamins among Human Species of Bifidobacteria. *Agric. Biol. Chem.* 49, 13–19. doi:10.1080/00021369.1985.10866683.

Delwiche, E. A. (1949). VITAMIN REQUIREMENTS OF THE GENUS PROPIONIBACTERIUM. *J. Bacteriol.* 58, 395–398.

Derrien, M., Vaughan, E. E., Plugge, C. M., and de Vos, W. M. (2004). Akkermansia muciniphila gen. nov., sp. nov., a human intestinal mucin-degrading bacterium. *Int. J. Syst. Evol. Microbiol.* 54, 1469–1476. doi:10.1099/ijs.0.02873-0.

Falentin, H., Deutsch, S.-M., Jan, G., Loux, V., Thierry, A., Parayre, S., et al. (2010). The Complete Genome of Propionibacterium freudenreichii CIRM-BIA1T, a Hardy Actinobacterium with Food and Probiotic Applications. *PLoS ONE* 5. doi:10.1371/journal.pone.0011748.

Forsberg, C. W. (1978). Nutritional characteristics of Megasphaera elsdenii. *Can. J. Microbiol.* 24, 981–985.

Fuchs, A. R., and Bonde, G. J. (1957). The nutritional requirements of Clostridium perfringens. *J. Gen. Microbiol.* 16, 317–329. doi:10.1099/00221287-16-2-317.

Giannella, R. A., Broitman, S. A., and Zamcheck, N. (1971). Vitamin B12 uptake by intestinal microorganisms: mechanism and relevance to syndromes of intestinal bacterial overgrowth. *J. Clin. Invest.* 50, 1100–1107.

Goldner, S. B., Solberg, M., and Post, L. S. (1985). Development of a minimal medium for Clostridium perfringens by using an anaerobic chemostat. *Appl. Environ. Microbiol.* 50, 202–206.

Goodman, A. L., McNulty, N. P., Zhao, Y., Leip, D., Mitra, R. D., Lozupone, C. A., et al. (2009). Identifying genetic determinants needed to establish a human gut symbiont in its habitat. *Cell Host Microbe* 6, 279–289. doi:10.1016/j.chom.2009.08.003.

Grossowicz, N., Kindler, S. H., and Mager, J. (1956). Nutritional studies with the Clostridium botulinum group. *J. Gen. Microbiol.* 15, 386–393. doi:10.1099/00221287-15-2-386.

Hébert, E. M., Raya, R. R., and de Giori, G. S. (2004). Nutritional requirements of Lactobacillus delbrueckii subsp. lactis in a chemically defined medium. *Curr. Microbiol.* 49, 341–345. doi:10.1007/s00284-004-4357-9.

Heinken, A., Khan, M. T., Paglia, G., Rodionov, D. A., Harmsen, H. J. M., and Thiele, I. (2014). Functional Metabolic Map of Faecalibacterium prausnitzii, a Beneficial Human Gut Microbe. *J. Bacteriol.* 196, 3289–3302. doi:10.1128/JB.01780-14.

Herbeck, J. L., and Bryant, M. P. (1974). Nutritional Features of the Intestinal Anaerobe Ruminococcus bromii. *Appl. Microbiol.* 28, 1018–1022.

Himmi, E. H., Bories, A., and Barbirato, F. (1999). Nutrient requirements for glycerol conversion to 1,3-propanediol by Clostridium butyricum. *Bioresour. Technol.* 67, 123–128. doi:10.1016/S0960-8524(98)00109-6.

Hugenschmidt, S., Schwenninger, S. M., Gnehm, N., and Lacroix, C. (2010). Screening of a natural biodiversity of lactic and propionic acid bacteria for folate and vitamin B12 production in supplemented whey permeate. *Int. Dairy J.* 20, 852–857. doi:10.1016/j.idairyj.2010.05.005.

Karasawa, T., Ikoma, S., Yamakawa, K., and Nakamura, S. (1995). A defined growth medium for Clostridium difficile. *Microbiol. Read. Engl.* 141 (Pt 2), 371–375. doi:10.1099/13500872-141-2-371.

Keuth, S., and Bisping, B. (1994). Vitamin B12 production by Citrobacter freundii or Klebsiella pneumoniae during tempeh fermentation and proof of enterotoxin absence by PCR. *Appl. Environ. Microbiol.* 60, 1495–1499.

Khan, M. T., Duncan, S. H., Stams, A. J. M., van Dijl, J. M., Flint, H. J., and Harmsen, H. J. M. (2012). The gut anaerobe Faecalibacterium prausnitzii uses an extracellular electron shuttle to grow at oxic–anoxic interphases. *ISME J.* 6, 1578–1585. doi:10.1038/ismej.2012.5.

Kopp, M., Dürr, K., Steigleder, M., Clavel, T., and Rychlik, M. (2017). Measurements of Intra- and Extra-Cellular 5-Methyltetrahydrofolate Indicate that Bifidobacterium Adolescentis DSM 20083T and Bifidobacterium Pseudocatenulatum DSM 20438T Do Not Actively Excrete 5-Methyltetrahydrofolate In vitro. *Front. Microbiol.* 8. doi:10.3389/fmicb.2017.00445.

Lawrence, J. G., and Roth, J. R. (1996). Evolution of Coenzyme B(12) Synthesis among Enteric Bacteria: Evidence for Loss and Reacquisition of a Multigene Complex. *Genetics* 142, 11–24.

Letort, C., and Juillard, V. (2001). Development of a minimal chemically-defined medium for the exponential growth of Streptococcus thermophilus. *J. Appl. Microbiol.* 91, 1023–1029.

Masuda, M., Ide, M., Utsumi, H., Niiro, T., Shimamura, Y., and Murata, M. (2012). Production potency of folate, vitamin B(12), and thiamine by lactic acid bacteria isolated from Japanese pickles. *Biosci. Biotechnol. Biochem.* 76, 2061–2067. doi:10.1271/bbb.120414.

McDonald, I. J., and Johnson, K. G. (1975). Nutritional requirements of some non-pathogenic Neisseria grown in simple synthetic media. *Can. J. Microbiol.* 21, 1198–1204.

Moore, S. J., Mayer, M. J., Biedendieck, R., Deery, E., and Warren, M. J. (2014). Towards a cell factory for vitamin B12 production in Bacillus megaterium: bypassing of the cobalamin riboswitch control elements. *New Biotechnol.* 31, 553–561. doi:10.1016/j.nbt.2014.03.003.

Pompei, A., Cordisco, L., Amaretti, A., Zanoni, S., Matteuzzi, D., and Rossi, M. (2007). Folate Production by Bifidobacteria as a Potential Probiotic Property. *Appl. Environ. Microbiol.* 73, 179–185. doi:10.1128/AEM.01763-06.

Rao, D. R., Reddy, A. V., Pulusani, S. R., and Cornwell, P. E. (1984). Biosynthesis and Utilization of Folic Acid and Vitamin B12 by Lactic Cultures in Skim Milk. *J. Dairy Sci.* 67, 1169–1174. doi:10.3168/jds.S0022-0302(84)81419-8.

Rogosa, M., and Bishop, F. S. (1964). THE GENUS VEILLONELLA II. *J. Bacteriol.* 87, 574–580.

Roper, J. M., Raux, E., Brindley, A. A., Schubert, H. L., Gharbia, S. E., Shah, H. N., et al. (2000). The Enigma of Cobalamin (Vitamin B12) Biosynthesis in Porphyromonas gingivalis. Identification and characterization of a functional corrin pathway. *J. Biol. Chem.* 275, 40316–40323. doi:10.1074/jbc.M007146200.

Salvetti, S., Celandroni, F., Ghelardi, E., Baggiani, A., and Senesi, S. (2003). Rapid determination of vitamin B2 secretion by bacteria growing on solid media. *J. Appl. Microbiol.* 95, 1255–1260.

Santos, F., Wegkamp, A., de Vos, W. M., Smid, E. J., and Hugenholtz, J. (2008). High-Level Folate Production in Fermented Foods by the B12 Producer Lactobacillus reuteri JCM1112. *Appl. Environ. Microbiol.* 74, 3291–3294. doi:10.1128/AEM.02719-07.

Storari, M., Kulli, S., Wüthrich, D., Bruggmann, R., Berthoud, H., and Arias-Roth, E. (2016). Genomic approach to studying nutritional requirements of Clostridium tyrobutyricum and other Clostridia causing late blowing defects. *Food Microbiol.* 59, 213–223. doi:10.1016/j.fm.2016.05.013.

SUGAHARA, H., ODAMAKI, T., HASHIKURA, N., ABE, F., and XIAO, J. (2015). Differences in folate production by bifidobacteria of different origins. *Biosci. Microbiota Food Health* 34, 87–93. doi:10.12938/bmfh.2015-003.

Testerman, T. L., Conn, P. B., Mobley, H. L. T., and McGee, D. J. (2006). Nutritional Requirements and Antibiotic Resistance Patterns of Helicobacter Species in Chemically Defined Media. *J. Clin. Microbiol.* 44, 1650–1658. doi:10.1128/JCM.44.5.1650-1658.2006.

Tsai, H.-N., and Hodgson, D. A. (2003). Development of a Synthetic Minimal Medium for Listeria monocytogenes. *Appl. Environ. Microbiol.* 69, 6943–6945. doi:10.1128/AEM.69.11.6943-6945.2003.

Varel, V. H., and Bryant, M. P. (1974). Nutritional Features of Bacteroides fragilis subsp. fragilis. *Appl. Microbiol.* 28, 251–257.

Wegkamp, A., Teusink, B., de Vos, W. M., and Smid, E. J. (2010). Development of a minimal growth medium for Lactobacillus plantarum. *Lett. Appl. Microbiol.* 50, 57–64. doi:10.1111/j.1472-765X.2009.02752.x.

Whitmer, M. E., and Johnson, E. A. (1988). Development of improved defined media for Clostridium botulinum serotypes A, B, and E. *Appl. Environ. Microbiol.* 54, 753–759.
